# Spectroscopical and Molecular Studies of Four Manganese(I) PhotoCORMs with Bioinspired Ligands Containing Non-Coordinated Phenol Groups

**DOI:** 10.3390/molecules28083439

**Published:** 2023-04-13

**Authors:** Matheus S. S. Paqui, Vinícius A. Glitz, Daniele C. Durigon, André L. Amorim, Giovanni F. Caramori, Renato L. T. Parreira, Adailton J. Bortoluzzi, Fernando R. Xavier, Rosely A. Peralta

**Affiliations:** 1 Departamento de Química, Universidade Federal de Santa Catarina (*UFSC*), Florianópolis 88040-900, SC, Brazil; matheus.paqui@posgrad.ufsc.br (M.S.S.P.); vinicius.glitz@posgrad.ufsc.br (V.A.G.); daniele.cocco.durigon@gmail.com (D.C.D.); andreamorim.enq@gmail.com (A.L.A.); giovanni.caramori@ufsc.br (G.F.C.); adailton.bortoluzzi@ufsc.br (A.J.B.); 2 Núcleo de Pesquisas em Ciências Exatas e Tecnológicas, Universidade de Franca, Franca 14404-600, SP, Brazil; renato.parreira@unifran.edu.br; 3 Departamento de Quimica CCT, Universidade do Estado de Santa Catarina (*UDESC*), Campus Joinville, Joinville 89219-710, SC, Brazil

**Keywords:** photoCORM, metal carbonyl compounds, manganese complexes

## Abstract

Carbonyl compounds are widely explored in medicinal inorganic chemistry and have drawn attention due to their signaling functions in homeostasis. Carbon-monoxide-releasing molecules (CORMs) were developed with the purpose of keeping the CO inactive until its release in the intracellular environment, considering its biological relevance. However, for therapeutic applications, the mechanisms of photorelease and which electronic and structural variations influence its rates must be fully understood. In this work, four ligands containing a pyridine, a secondary amine, and a phenolic group with different substituents were used to prepare new Mn(I) carbonyl compounds. Structural and physicochemical characterization of these complexes was carried out and confirmed the proposed structures. X-ray diffractometry structures obtained for the four organometallic compounds revealed that the substituents in the phenolic ring promote only negligible distortions in their geometry. Furthermore, UV-Vis and IR kinetics showed the direct dependence of the electron-withdrawing or donating ability of the substituent group, indicating an influence of the phenol ring on the CO release mechanism. These differences in properties were also supported by theoretical studies at the DFT, TD-DFT, and bonding situation analyses (EDA-NOCV). Two methods were used to determine the CO release constants (k_CO,old_ and k_CO,new_), where Mn-HbpaBr (1) had the greatest k_CO_ by both methods (K_co,old_ = 2.36 × 10^−3^ s^−1^ and k_CO,new_ = 2.37 × 10^−3^ s^−1^). Carbon monoxide release was also evaluated using the myoglobin assay, indicating the release of 1.248 to 1.827 carbon monoxides upon light irradiation.

## 1. Introduction

Carbon monoxide signaling functions have been extensively described in the literature [1,2,3]. They can regulate cellular homeostasis by inducing anti-inflammatory [4], anti-apoptotic [5], tumoral antiproliferative [6], vasodilator [7], and lung-disease-mitigating activities [8], which has led to an interest in exploring their therapeutic use. The first therapeutic approach was the use of gaseous carbon monoxide through low-concentration inhalation [9]. Although toxicological effects were not observed in these studies, the safety of this treatment is controversial, resulting in the search for pharmacological alternatives. Compounds capable of releasing carbon monoxide upon different forms of stimuli have been named CORMs (carbon-monoxide-releasing molecules). Dimanganese decacarbonyl [Mn_2_(CO)_10_], which is capable of releasing CO due to an exchange of ligands with a solvent [10], was initially explored with this purpose, and compounds were developed by applying different types of triggers, such as enzymatic [11] and pH variation [12]. However, all these forms of stimulus cannot be controlled externally to the physiological system. Light-activated CORMs are called photoCORMs [13,14]. Light is the most explored inducement for CO release, which allows the process to be initiated noninvasively and enhances the control of the dosage of CO released in the system, while also reducing potential side effects of the treatment in healthy tissues in the case of low selectivity of the drugs to the areas of interest [15]. The first developed photoCORMs required UV light to generate CO, which has low tissue penetration and could cause a series of complications from the treatment itself [16]. The use of lower energies, such as visible and near-infrared (NIR) light, although already achieved, is still a challenge in the development of new photoCORMs [17]. The modulation of the necessary energy to promote CO release depends on the metal-to-ligand charge transfer (MLCT) wavelength, which weakens the π-backbonding in M–C bonds, thus promoting the CO release [18]. In order to achieve drug-like CORMs, it is necessary to combine the effects of the metal center, the coordination sphere, and the outer sphere [19]. The substitution of some CO in carbonyl compounds with ligands capable of lowering the energy of antibonding orbitals results in a bathochromic shift of the MLCT bands [20]. The auxiliary ligands could also promote better specificity for therapeutic applications, influencing the solubility of these compounds and their fluorescence. Several types of ligands could be designed to have these properties, but the auxiliary ligand could modify the mechanisms through which carbon monoxide is released, influencing the CO release rates and the generated byproducts of the photolysis process [21]. Bidentate and tridentate species are the most common types of ligands used in the development of organometallic photoCORMs. Bidentate coordinated tridentate ligands tend to chelate the third coordination site to the metallic center after CO release, a behavior that has been previously described by our group using ligands containing nitrogen [22], selenium, and sulfur as donor atoms [23]. In this work, we present four new manganese(I) photoCORMs containing an N,N,O donor set and investigate the electronic, physicochemical, and molecular properties of these compounds to explore the CO photoliberation and their rate constants. The ligands Hbpa-Br, Hbpa-H, Hbpa-Me, Hbpa-OMe, and their respective organometallic compounds Mn-HbpaBr (**1**), Mn-HbpaH (**2**), Mn-HbpaMe (**3**), and Mn-HbpaOMe (**4**) were synthesized, and the complexes were characterized using a diversity of structural and physicochemical techniques and were supported by theoretical studies.

## 2. Results and Discussion

**Figure 1:**
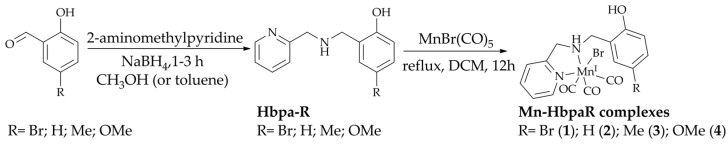
General schematic diagram for the synthetic procedures performed highlighting the ligands and complexes studied in this work.

All compounds studied in this work are presented in Figure 1. The ligands were prepared based on previous literature procedures [24,25]. Once their purity was confirmed, their metal carbonyl compounds were synthesized and single crystals suitable for X-ray diffraction were obtained for all complexes.

Since the ligands are well known and characterized [24,25], this work will focus on the discussion of the organometallic compounds (**1**)–(**4**). IR, UV-Vis, and ^1^H NMR spectra of the organic compounds Hbpa-R (R = Br, H, Me, OMe) are presented and briefly discussed in the Supplementary Materials section (Figures S1–S6).

### 2.1. Solid-State Characterization

Compounds (**1**)–(**4**) were initially characterized through solid-state techniques, confirming their purity (Section 3.5), and then analyzed through X-ray diffractometry and infrared spectroscopy, which are discussed below.

#### 2.1.1. X-ray Diffractometry

**Figure 2:**
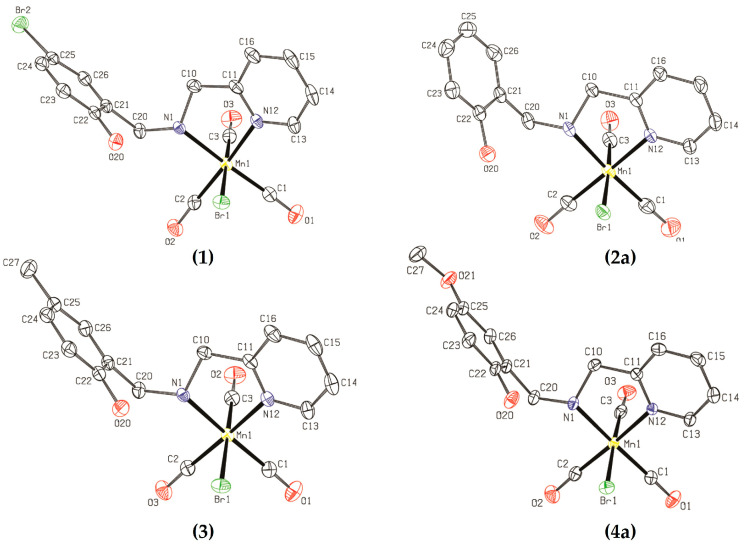
ORTEP plots of organometallic compounds (**1**)–(**4**) with ellipsoids drawn at a 50% probability level. Hydrogen atoms were omitted for clarity. Structures (**2a**) and (**4a**) were selected from the conformers and isomers, respectively. See (**2b**) and (**4b**) in Figure S7; gray = carbon, red = oxygen, green = bromide, blue = nitrogen, and yellow = manganese.

The proposed structures for all organometallic compounds were confirmed through X-ray diffractometry (Figure 2). Selected crystallographic data are available in the Supplementary Materials (Tables S1–S8). As hypothesized, the phenolic ring remains uncoordinated with the manganese(I) center, and the three carbonyl ligands are facially coordinated in all cases, which was also observed through IR spectroscopy (Section 2.1.2).

Mn-HbpaH (**2**) presents a monoclinic crystal system, while (**1**), (**3**)**,** and (**4**) have a triclinic one, which indicates that adding substituent groups to the phenol ring changes the molecular packing. The crystalline structure of complex (**2**) shows the presence of two crystallographically independent molecules (differentiated as (**2a**) and (**2b**)) in the asymmetric unit. The two molecules of (**2**) are conformers, with most differences being in the torsion angles of the phenol ring region. Complex (**4**) also has two independent molecules in the asymmetric unit, which are differentiated as (**4a**) and (**4b**), but, in this case, **4a** and **4b** are isomers in which the position of the carbonyl ligand is inverted with bromide. All molecular structures are presented in the Supplementary Materials section (Figure S7).

The main bond lengths are presented in Table 1. Mn–C(3) has the shortest, due to the π-backbonding, which is strengthened by the bromide in the *trans* position. C–Mn–C bond angles are close to 90°, indicating a negligible distortion of the octahedral geometry for all compounds.

Kunz and coworkers (2011) [26] developed photoCORMs containing di-(2-picolyl)amine (bpma) derivatives. In one compound, both pyridyl rings are coordinated with the manganese(I) center. Mn–C bonds for compound [(bpmea)Mn(CO)_3_]Br (**4**) were 1.820 Å, 1.813 Å, and 1.807 Å, respectively. The bpma ligand coordinates through pyridyl and amino groups in a tridentate mode. Even though bpma has three coordination sites, the electronic influence of both groups on the metal center is similar to that of the Hbpa, resulting in small variations of Mn–C bond lengths. A similar work in our group containing bpma derivatives (called **dpa** in this work) [22] presented similar values.

In a more recent work [27], a manganese(I) photoCORM containing a bpma derivative called NBD-CORM presented a Mn–N (*sp*^3^) bond length of 2.161 Å and a Mn–N (*sp*^2^) of 2.050 Å. Compounds (**1**)**–**(**4**) have an average Mn–N (*sp*^3^) bond length of 2.087 Å and Mn–N (*sp*^2^) of 2.056 Å. NBD-CORM also has a κ^3^ coordination mode and the larger difference from our compounds is associated with the manganese–amine bond, due to the increased rigidity of the ligand by coordination of the two pyridines in that case.

#### 2.1.2. IR Spectroscopy

**Figure 3:**
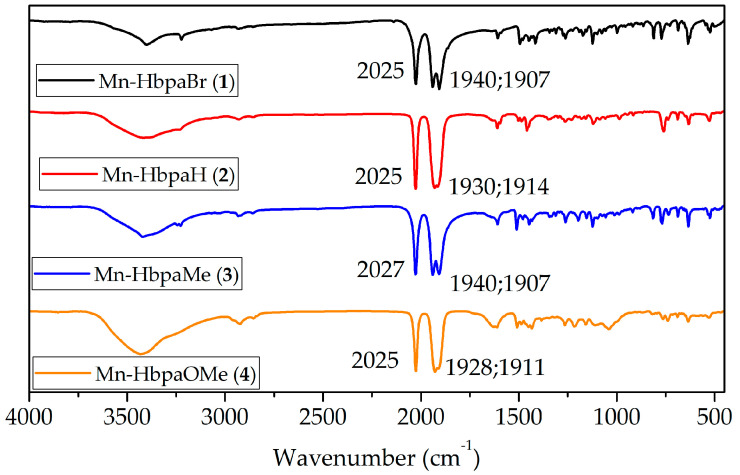
IR spectra of compounds (**1**)–(**4**) at 4.0 cm^−1^ resolution (FTIR, KBr pellets).

IR spectra of compounds (**1**)–(**4**) (Figure 3) were in accordance with the obtained X-ray structures. In the same way that the Mn–C bonds are not affected by the substituent group in the phenolic ring, the energy of the ν_(CO)_ stretching of the carbonyls presents similar values between the complexes (Figure S8).

The facial coordination mode of carbonyl ligands resulted in a local symmetry of a *pseudo*-*C*_3_*v* point group, where two absorption bands are active in IR, stemming from a symmetric stretching of *A*_1_ symmetry and two degenerate symmetric stretching of *E* symmetry [28].

For compounds (**1**)–(**4**), the symmetric stretching appears on 2025 cm^−1^, whereas the asymmetric stretches have maximum bands around 1935 cm^−1^ and 1911 cm^−1^. White and coworkers (2020) [21] developed six manganese(I) photoCORMs containing bipyridine derivatives to study the influence of the ligand set on the photorelease mechanism. They observed that the increase in the π-acceptor character of bipyridine due to substituents results in an increase in CO stretching energies due to a Mn–CO π-backbonding weakening. The substituent groups on compounds (**1**)–(**4**) do not affect CO stretching frequencies or Mn–C bond lengths because the phenolic ring is not coordinated with the metal center, which makes it more difficult to observe the direct electronic influence of the substituents.

The Ford group (2019) [29] developed a water-soluble manganese(I) photoCORM containing the ligand 4′-*p*-*N*,*N*-Bis(2-hydroxyethyl)amino-benzyl-2,2′:6′,2′′-terpyridine (TPYOH), which coordinates through pyridyl rings. For compound ***fac*-**[Mn(CO)_3_(TPYOH)Br] (**1**), CO stretching frequencies were 2017 cm^−1^, 1917 cm^−1^, and 1885 cm^−1^. Such considerable variation, especially on the asymmetric stretches, is a result of the ligand’s nature, indicating that TPYOH has lower π-acidity compared to compounds (**1**)–(**4**) from this work, resulting in an increase in the Mn–CO π-backbonding energy.

### 2.2. In-Solution Characterization

Spectroscopic and electrochemical techniques were employed to determine the behavior of compounds (**1**)–(**4**) in solution. The compounds were analyzed through UV-Vis, cyclic voltammetry, square wave voltammetry (Figures S9 and S10), ESI-MS (Figure S11), and ^1^H NMR (Figures S12–S15).

#### 2.2.1. UV-Vis Spectroscopy

**Figure 4:**
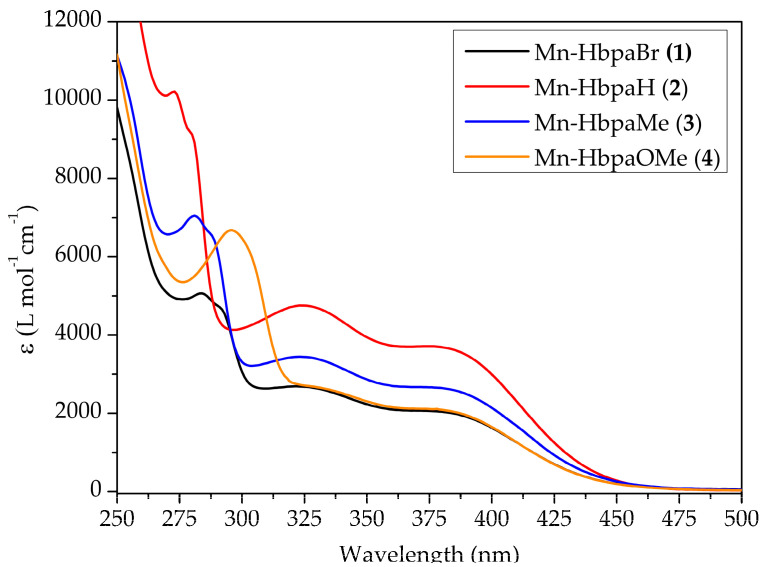
Electronic spectra of compounds (**1**)–(**4**) in dichloromethane. (**1**) = 1.513 × 10^−4^ mol L^−1^; (**2**) = 1.579 × 10^−4^ mol L^−1^; (**3**) = 1.592 × 10^−4^ mol L^−1^; (**4**) = 1.560 × 10^−4^ mol L^−1^.

Electronic spectra of compounds (**1**)–(**4**) are presented in Figure 4, for which three absorption bands are observable. The lowest energy band appears as a shoulder at around 379 nm. This band represents metal-to-ligand charge transfer (MLCT) electronic transitions [30]. Absorption band wavelengths are summarized in Table 2.

The MLCT band has the same absorption wavelength for all complexes, given that the substituent modifications are located on the phenolic ring, where its influence on the metallic center’s electronic density is negligible.

Since the development of the first photoCORM, much effort has been put into the development of visible and near-infrared photoactivated compounds. The CO-releasing mechanism depends on the photoexcitation on the MLCT region, which weakens M–C bonds and promotes CO release. It has been established that this bathochromic shift on similar manganese(I) compounds can be achieved through the combined effect of the π-donor character of the ancillary ligand and the electronic delocalization of the bidentate ligand [18,20,30]. Compounds (**1**)–(**4**) lack the necessary π-acidity coming from high electronic delocalization, making them UV active.

The absorption band at around 323 nm is not affected by the electronic influence of the substituent group, which indicates that it may not be associated with the phenolic ring. Hence, TD-DFT calculations were performed to identify the molecular orbitals involved in that electronic transition (Section 2.3). Those below 295 nm are associated with π*← n electronic transitions on the phenolic ring. [24,25,27].

#### 2.2.2. Electrochemistry

**Figure 5:**
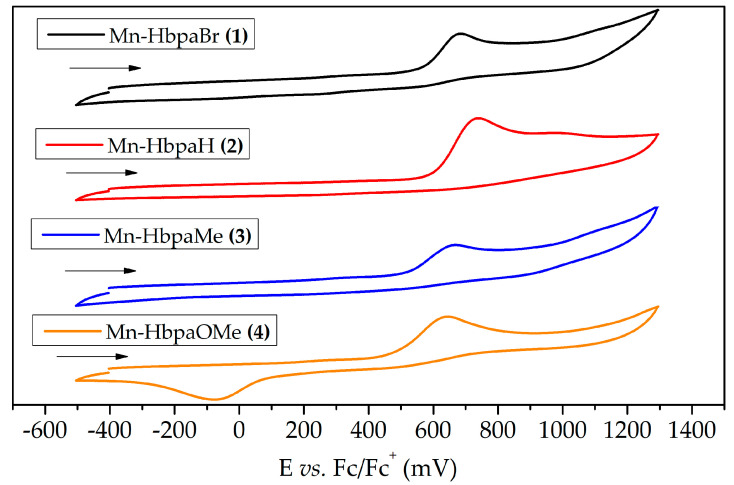
Cyclic voltammograms of compounds (**1**)–(**4**) in dichloromethane under argon atmosphere. Experimental conditions: 0.1 mol L^−1^ TBAPF_6_ as the supporting electrolyte, Ag/AgCl as the reference electrode, and platinum as the work and auxiliary electrodes. Potentials are referenced to the Fc/Fc^+^ couple (E_1/2_ = 0.405 V vs. Ag/AgCl in dichloromethane).

The redox potentials of complexes (**1**)–(**4**) were studied through cyclic voltammetry (CV) and square wave voltammetry (SWV) (Figure S9). Cyclic voltammograms (Figure 5 and Table 2) reveal an anodic Mn^I^ → Mn^II^ process. This oxidation is characteristically irreversible, given that, during Mn^II^ formation, the electron density on the metal center is hampered, weakening the π-backbonding with carbonyl ligands and resulting in their release from the coordination sphere [31,32].

Oxidation potentials share the same tendency with UV-Vis electronic transitions below 295 nm. The necessary potential to reach the anodic peak decreases as the electron-donating ability of the substituent group increases, indicating that the phenolic ring could have an influence over the electron density of the metal center. On the other hand, solid-state characterization and UV-Vis spectroscopy demonstrate that the phenolic ring does not influence the metal center’s electron density, once ν_CO_ stretching bands in IR are identical between the compounds, like MLCT electronic transitions have the same wavelength in UV-Vis. If the phenolic rings were coordinated with the manganese(I) center, the substituent group would have a direct influence over the electronic transitions and stretching bands.

UV-Vis bands below 295 nm, which are associated with the phenolic ring orbitals, have a specific tendency, where (**4**) has the least energetic electronic transition, just like it has the lower oxidation potential in CV. So, this behavior could be associated with the stability of Mn(II) species formed in this process.

We hypothesize that, after the Mn(I) oxidation, carbonyl ligands could be released from the coordination sphere, making the phenolic ring susceptible to coordinate with the electron-deficient Mn(II) center. In this case, the π-donating ability of the substituent group would influence the stability of the formed species, which would make compound (**4**) the most thermodynamically favored during Mn(I) oxidation.

Once again, compound (**1**) deviates from this tendency. Although bromide is characterized as an electron-withdrawing substituent, it can donate electron density through π orbitals, which results in a similar behavior to that of compound (**3**).

White and collaborators (2021) [31] developed a manganese(I) photoCORM containing a BODIPY-based ligand (Mn-bpy-H-BDP), which presented a Mn^I^ → Mn^II^ oxidation potential of 0.75 V vs. Fc/Fc^+^. This potential is similar to potentials encountered for compounds (**1**)–(**4**) (summarized in Table 2). The bipyridine moiety, which is coordinated with the manganese(I) center, results in a similar influence of the ligand over the electron density at the metal center. The high oxidation potentials are mostly influenced by the carbonyl ligands, which can hamper the stability of compounds in higher oxidation states due to its π-acceptor character [32].

Another oxidation process is observable for (**2**) at 0.972 V, as a reduction process is observable for (**4**) at −0.081 V. Both processes can be associated with the organic moiety, given that the phenol ring has its own redox behavior [33]. Cyclic voltammetry of compound (**4**) was also measured with the inversion of the scan direction to investigate if the reduction process was dependent on the manganese(I) oxidation (Figure S10). The same process was observed, indicating that it is associated with the organic moiety and not the metal center.

#### 2.2.3. Mass Spectrometry (ESI-MS)

The mass spectra of compounds (**1**)–(**4**) (Figure S11) presented a similarity: the base peak represents the cationic species formed after bromide removal [Mn(HbpaR)(CO)_3_]^+^. Once the complexes were dissolved in acetonitrile, the bromide was already expected to be exchanged for a solvent molecule (Section 2.4).

Compound (**1**) showed its base peak at 430.86 *m/z*, indicating an unaltered structure besides the bromide withdrawal. The same behavior occurred for compound (**2**), with a prominent signal at 352.93 *m/z*. With the bromide exchange, the complexes already became cationic, and, for compounds (**1**) and (**2**), no other substantial fragmentations were observed.

Compound (**3**) presented three intense peaks: the base peak at 366.96 *m/z* ([Mn(HbpaMe)(CO)_3_]^+^), a fragmentation at 242.18 *m/z*, and another fragmentation at 456.00 *m/z*. In compound (**4**), the base peak appears at 382.96 *m/z* ([Mn(HbpaOMe)(CO)_3_]^+^), and two other fragmentations of lower intensity at 472.00 *m/z* and 763.07 *m/z*. The 16 *m/z* difference between the 456 *m/z* in compound (**3**) and the 472 *m/z* in compound (**4**) indicates that identical species are formed in this fragmentation.

#### 2.2.4. ^1^H NMR Spectroscopy

^1^H NMR spectra of compounds (**1**)–(**4**) in deuterated acetone are shown in Figures S12–S15. The spectra indicated the purity of the complexes in solution. Diastereotopic hydrogens were observed in the methylene groups for all complexes. In comparison with the free ligands, the coordination promotes an increase in the rigidity of the structure, making the two hydrogen atoms distinguishable in each methylene group. Furthermore, different groups near those hydrogens can influence their electronic shielding.

Besides the presence of the methylene hydrogens’ signals, the aromatic hydrogens from the pyridine and the phenol group were present and the integration of the signals corresponded with the number of aromatic hydrogens. The NH signal was also observed in all cases above 9.0 ppm, with minimal shift between the complexes, while the OH signal presented greater variation, once the substituent groups on the phenol ring had a direct impact on the electronic shielding of the hydroxyl group.

### 2.3. Computational Studies

To perform the computational study, the structures obtained through X-ray were used as the starting point, considering the obtained isomers. Initially, the structures were optimized in a vacuum and the analytical frequencies were obtained, showing a good correspondence between the calculated and experimental results, validating the parameters used in the calculations (Section 3.5). In Figures S16–S19, a comparison between the calculated and experimental results is presented, where a very good correlation of CO stretching values can be observed (Figure S20). Regarding the conformational structures of compound (**2**) and the isomers of compound (**4**) observed in X-ray diffraction, the relative difference in the final Gibbs free energy was 0.11 kcal mol^−1^ and 0.02 kcal mol^−1^ for (**2**) and (**4**), respectively. In the solid-state and solution characterizations as well, they showed no significant variation.

**Figure 6:**
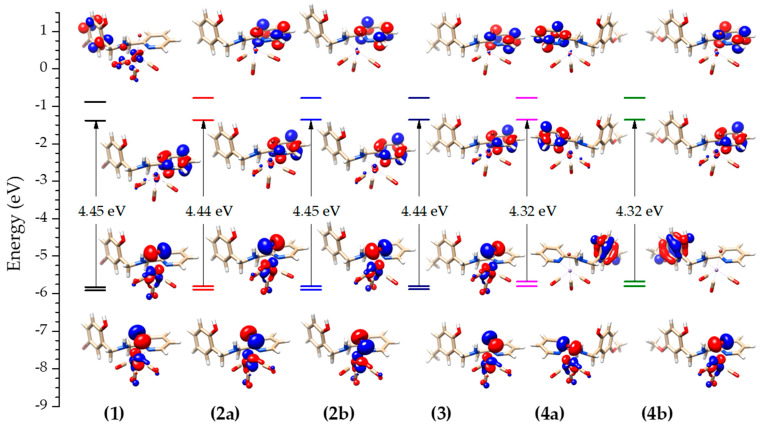
Molecular orbital diagram with the obtained frontier orbitals for all compounds at level B3LYP/def2-TZVP for Mn and Br, and B3LYP/def2-TZVP(−f) for the other atoms in Orca software version 5.0.3.

A new optimization was carried out considering the solvent dichloromethane, used in the characterizations and CO photorelease, to obtain the electronic spectra and the frontier orbitals of the studied compounds (Figure 6). As observed using the other experimental techniques, the frontier orbitals of the conformers of compound (**2**) and isomers of compound (**4**) do not show any substantial difference. The molecular orbitals HOMO-1 of all compounds have the same profile, with contributions from the *p_z_* and *p_y_* orbitals of the bromide, *d_z_^2^* and *d_xy_* of the Mn(I) and a small contribution from the π orbitals of the carbonyls. The HOMO orbitals from the isomers (**4**) have the electronic density on the uncoordinated phenol. Compounds (**1**)–(**3**) have contributions from the *p_x_* and *p_y_* orbitals of the bromide, *d_x_*^2^*_−y_*^2^ and *d_yz_* of the Mn(I), and π orbitals of the CO. The LUMO orbitals are composed of the π orbitals of the pyridyl group coordinated to the manganese(I), as well as the LUMO+1 orbitals of compounds (**2**), (**3**), and (**4**), which, in turn, are composed of the π* orbitals of the pyridyl group. Differently, compound (**1**) has its LUMO + 1 centered on the phenolic group with a small contribution from the manganese(I) and carbonyls.

Electronic spectra of all complexes were calculated considering a TD-DFT approach. Table S9 presents the values for the main electronic transitions involved, and Figures S21–S24 show the overlap between the experimental and calculated electronic spectra. The lower energy bands are attributed to the metal–ligand charge transfer (MLCT), being a transition of the electron density composed of the Br–Mn–CO orbitals to the π* orbitals of the pyridyl group (HOMO − 1 → LUMO and HOMO → LUMO of compounds (**1**)–(**4**). It is interesting to note that compounds (**2**), (**3**), and (**4**) also show a contribution of a charge transfer between the HOMO − 1 orbitals located on the Br–Mn–CO to the LUMO + 2 orbitals located on the π* orbitals of the phenolic moiety, which does not occur in compound (**1**)**,** where this orbital is the LUMO + 1. The transition located in the region of ≈327 nm is characteristic of an MLCT, being a transition from the HOMO → LUMO orbitals of compounds (**1**) to (**3**) and HOMO − 1 → LUMO of (**4**). At higher energies, a transition from the π orbitals of the phenolic moiety to the π* orbital is observed with a small contribution from the Mn–CO orbitals.

Calculations regarding the energy decomposition analysis (EDA) were carried out to obtain information about the bonding strength between each carbonyl and the rest of the molecule, in addition to the effects of substituent groups used in the phenolic portion in this interaction. The results are summarized in Table 3. The 1-CO fragment is the carbonyl *trans* to bromide, 2-CO is *trans* to pyridyl group, and 3-CO is *trans* to the amino group, as shown in Figure S25. The interaction energy (ΔE_int_) for the 1-CO fragment is the most stable (≈45 kcal mol^−1^) compared to the 2-CO and 3-CO fragments (≈36 kcal mol^−1^ and ≈37 kcal mol^−1^, respectively), indicating that the 1-CO interacts more strongly with the metal center than the other two fragments. This is related to the fact that the bromide is *trans* to this fragment, as it is a σ-donor and a better donor than the pyridyl and amino groups. Consequently, it will donate a greater electron density to the metal center, making the *trans* Mn–CO bond more stable compared to the other two carbonyl groups. Comparing the 2-CO and 3-CO fragments shows that the one *trans* to the amino group is slightly more stable than the CO *trans* to the pyridyl group.

The destabilization of the Mn–CO interaction is represented by the Pauli repulsion (ΔE_Pauli_), which showed a small variation between the three analyzed fragments (minimum of 166.11 kcal mol^−1^ and maximum of 169.66 kcal mol^−1^). The stabilization of the Mn–CO interactions is composed of the attractive forces, which are the sum of the electrostatic interaction (ΔE_elstat_), the orbital interaction (ΔE_oi_), and the dispersion energy (ΔE_disp_). On average, the ΔE_elstat_ contribution is 54.7%, the ΔE_oi_ is 44.2%, and the ΔE_disp_ is 1.1%, showing a variation of ±1.1%, ±1.0%, and ±0.1%, respectively. Therefore, the substituent groups present in the phenolic portion do not directly influence the energy values between the three carbonyl groups and the metal center. The difference between the values is directly influenced by the groups/atom present in the position *trans* to the carbonyl ligand, as can be observed for the values of ΔE_Pauli_, ΔE_elstat_, and ΔE_oi_, which were higher (ΔE_Pauli_) and lower (ΔE_elestat_ and ΔE_oi_) for the fragment *trans* to the bromide (1-CO) compared to the 2-CO and 3-CO fragments *trans* to the N_py_ and N_aliph_, respectively.

**Figure 7:**
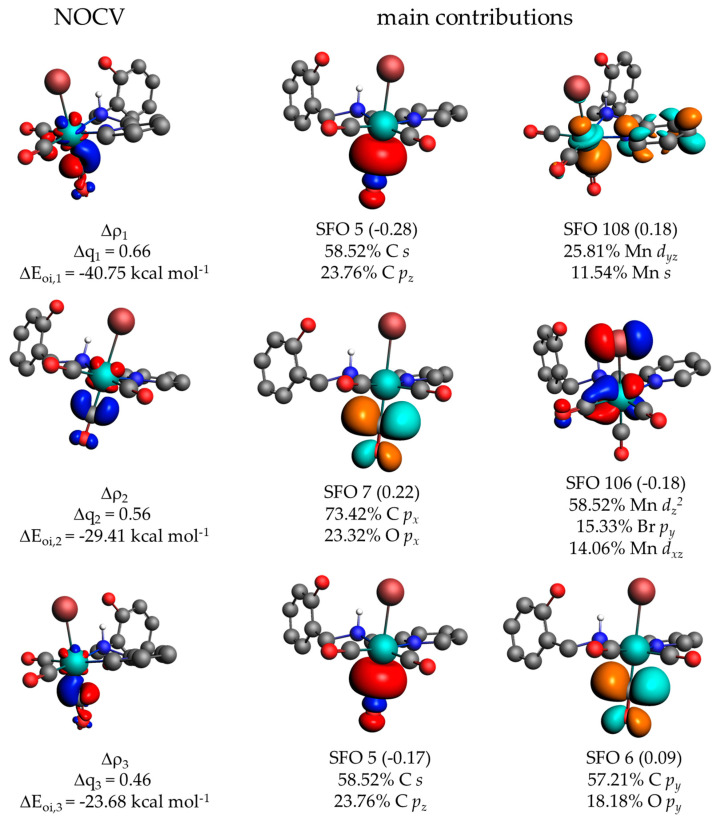
Plot of the most relevant density flow channel (Δρ) contributing more than 2.0 kcal mol^−1^ with their respective energies (ΔE_oi_) and charge transfer estimation (Δq) values for fragment 1-CO (*trans* to bromide) of compound (**2a**) and the associated orbitals of the fragments with the greatest contribution for each density flow channel. The direction of the charge flow is red to blue, where red means density depletion, and blue means density increment. Hydrogens attached to carbon are omitted for clarity. The isovalue for the NOCV is 0.005, and for the SFO it is 0.05.

The orbital interactions can be separated into the contributions σ and π (ΔE_oi-σ_ and ΔE_oi-π_ in Table 4), where the π interaction contributes about 11% more than the σ interaction. By performing the energy decomposition analysis–natural orbitals for chemical valence (EDA-NOCV), these contributions can be visualized, leading to the identification of individual deformation density channels accounting for contributions from each set of interacting orbitals. The EDA-NOCV shows a direct separation of the contributions to the deformation density from the metal-to-ligand and ligand-to-metal electron transfer process, as shown in Figure 7 and Figures S26 and 27 for compound (**2a**), 1-CO, 2-CO, and 3-CO, respectively. The other manganese carbonyl compounds showed the same behavior, as observed in Table S10. The three main orbitals involved in the Mn–CO interaction can be identified. The first flow channel (Δρ_1_) describes the formation of the σ-bond between the manganese and the carbonyl, with a charge transfer (Δq_1_) estimated at 0.66, 0.62, and 0.62 for 1-CO, 2-CO, and 3-CO fragments, respectively. The second and third density flow channels describe a π-bond interaction, with Δq_2_ = 0.56, 0.52, and 0.52 and Δq_3_ = 0.46, 0.48, and 0.47 for 1-CO, 2-CO, and 3-CO, respectively. The values of ΔE_oi_ show that the energy of the π-bond for the 1-CO fragment is more stable compared to the 2-CO and 3-CO, a consequence of the *trans* effect, as the bromide will further strengthen the π-backbonding compared to the pyridyl and amino group. The main associated symmetrized fragment orbitals (SFO) that contribute to the formation of the three density flow channels are shown in Figure 7 and Figures S26 and S27, which show the participation of carbon *s* and *p* orbitals and manganese *d* orbitals.

### 2.4. Determination of Active Species in Solution

Stability tests of compounds (**1**)–(**4**) in solution were performed through UV-Vis, IR spectroscopy, and molar conductivity measurements. To characterize the solvent effect on the manganese coordination sphere, studies were performed in non-coordinating (dichloromethane) and coordinating (acetonitrile) solvents.

The spectral profile of the compounds herein studied indicated that the dichloromethane does not promote ligand exchange or oxidation of the metal center, given that MLCT transitions remain unaltered (Figure S28). A small hyperchromic shift is observable in all cases, associated with solvent evaporation, due to its high volatility.

**Figure 8:**
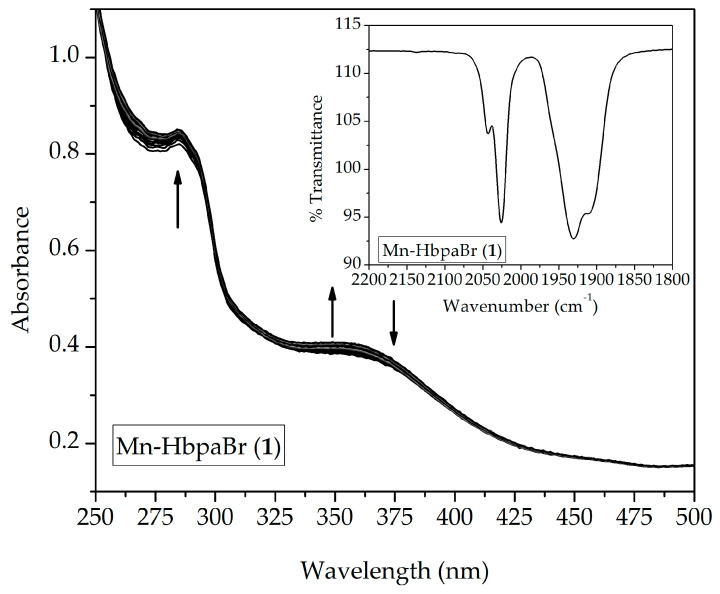
Spectral changes of (**1**) acquired through UV-Vis spectroscopy. Spectra were collected in a 1 h interval in CH_3_CN. Sealed quartz cuvettes were kept in the dark during the whole experiment. [(**1**)] = 2.05 × 10^−4^ mol L^−1^. Inset: IR spectra of (**1**) (KBr pellets) performed with 100 µL of stock solution (1.00 × 10^−3^ mol L^−1^) after 24 h in the dark.

A different behavior was observed when compounds were dissolved in CH_3_CN. The spectral analyses of the compounds (Figure 8 and Figures S29–S31) demonstrate a hypsochromic shift of MLCT transitions. This behavior is associated with the substitution of the bromide ligand for a solvent molecule [23,30]. Acetonitrile has a lower π-donator character, weakening the π-backbonding with carbonyl ligands, but not enough to promote CO release, given that no hypochromic shift is observable.

**Figure 9:**
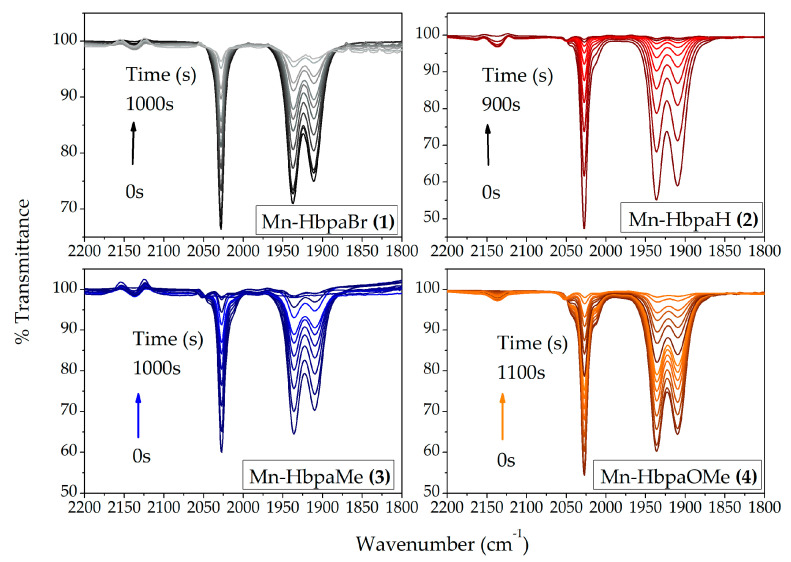
Changes in IR spectra of compounds (**1**)–(**4**) during light irradiation (λ_em_ = 395 ± 5 nm). Compounds were dissolved in dichloromethane with final concentrations of 1.00 × 10^−3^ mol L^−1^.

For compound (**4**), the energy band shift is evident, making an isosbestic point observable (Figure S31). The presence of the isosbestic point indicates equilibrium of the species in solution. The inset graphs of Figure 9 and Figures S29–S31 present IR spectra of solutions kept in the dark for 24 h. Beyond the original ν(_CO_) stretching bands, a shoulder of lower intensity can be observed at a 20 cm^−1^ higher energy. This indicates that there is a mixture between the original species, [Mn(κ^2^-Hbpa-R)(CO)_3_Br], and the solvent substituted species, [Mn(κ^2^-Hbpa-R)(CO)_3_(CH_3_CN)]^+^. IR measurements corroborate the idea that CO is not released from the coordination sphere after the bromide substitution.

The formation of cationic species in acetonitrile would result in variations in the conductivity of the solutions. In this sense, molar conductivity measurements were performed for compounds (**1**)–(**4**) in acetonitrile and dichloromethane at 0 h and 24 h relative to the preparation of the solutions. Results corroborate UV-Vis and IR implications (Table S11). Compounds are highly stable in dichloromethane, while a partial substitution of bromide ligand occurs during dissolution of the compounds in acetonitrile (increasing their molar conductivity), which tends to reach an equilibrium between original and solvent-substituted species within 24 h.

### 2.5. CO Release Assays

After the characterization of the formed species in solution, CO release studies of compounds (**1**)–(**4**) were performed qualitatively via IR spectroscopy and quantitatively via UV-Vis spectroscopy. In acetonitrile, the original species reach equilibrium states with solvent-substituted species, which could have a direct impact on the CO release mechanism. To characterize the effect of the phenolic ring, we opted to perform the studies on dichloromethane, which does not alter compound structures.

A qualitative analysis of CO release is presented in Figure 9. All compounds presented the same spectroscopic behavior. During irradiation using violet light (λ_em_ = 395 ± 5 nm) over the solutions, ν_(CO)_ stretching bands gradually decreased until they completely disappeared.

Interestingly, no new stretching bands arose at this IR region. Similar works were able to identify the presence of biscarbonyl intermediate species [27,34,35]. After the first carbonyl release, the remaining carbonyl groups would adopt a *pseudo*-*C*_2*v*_ group point, which results in two stretching bands of *A*_1_ and *B*_1_ symmetry. The *B*_1_ stretching band usually appears at around 1850 cm^−1^. The absence of biscarbonyl stretching bands indicates that this species is unstable, and the other two carbonyl ligands should be quickly released from the coordination sphere, making this intermediate undetectable [34].

All compounds were capable of releasing CO upon light exposure. Beyond the decrease in carbonyl stretching bands, a free carbon monoxide stretching band appears at 2140 cm^−1^, confirming the CO release [28].

**Figure 10:**
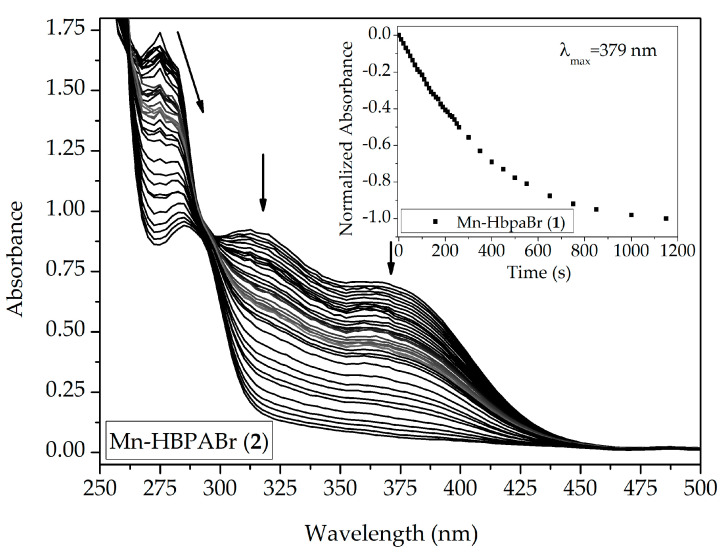
Changes in UV-Vis spectrum of (**1**) in dichloromethane (4.12 × 10^−4^ mol L^−1^) during UV light irradiation (λ_em_ = 395 ± 5 nm). Inset: Normalized absorbance decay of (**1**) at 379 nm as a function of time (s) during light exposure.

The photorelease ability of compounds (**1**)–(**4**) was also observed through UV-Vis spectroscopy. Compounds were diluted in dichloromethane to final solutions of 2.0 × 10^−4^ mol L^−1^ and irradiated with the same set of light-emitting diodes (LEDs) used in the IR experiments. The solutions were irradiated until no further spectral variations were observed. The photorelease experiment for (**1**) is presented in Figure 10. Compounds (**2**)–(**4**) are presented in the Supplementary Materials (Figures S32–S34).

All compounds presented in this work showed the same behavior upon light exposure, in which the MLCT band decreased until completely it disappeared. This is another confirmation that carbonyl is released upon violet light irradiation. The absorption band at 323 nm underwent the same influence. This is in accordance with the TD-DFT calculations, which indicate that this band is characterized as another CT process. For both cases, π-antibonding orbitals from carbonyl ligands are associated with the electronic transition. After the CO release, no absorption bands are expected in this region.

The absorption band below 295 nm suffers a bathochromic shift upon light exposure. This band is associated with electronic transitions on the phenol ring. UV-Vis characterization of **Hbpa-R** ligands (Figure S2) indicates that the free ligands have blue-shifted absorption bands when compared to their respective photoCORMs, so the phenolic ring should be coordinating with the metal center upon CO release, which is in accordance with electrochemical experiments.

The UV-Vis photorelease experiment was used to determine CO release rate constants (k_CO_), process quantum yield (Φ), and the half-life time (Table 4). CO release rates were obtained through two different mathematical approaches. In order to compare our results with the literature, a pseudo-first-order kinetic model through the linearization of the normalized absorbance as a function of time was calculated (here described as kco,_old_). The k_CO_ was also determined using an exponential decay fit (k_CO,new_). Additionally, absorbances were normalized to promote a direct comparison between the compounds. The complete equations are described in the Supplementary Materials.

Compound (**1**) presented the greatest CO release constant using both methods. The evidence gathered so far should be considered to discuss these results. IR photorelease experiments indicate that, if a biscarbonyl species is formed after the release of the first carbonyl, it is highly unstable (as it could not be observed) and quickly undergoes consequent reactions, including phenol (or phenolate) coordination and the release of the remaining carbonyl groups. UV-Vis spectra also indicate the coordination of the phenol group. Compound (**1**) possesses a bromide substituent on the phenol ring, which has the greatest electron-withdrawing ability of this series. In consequence, the [Mn(κ^3^-L)CO_2_Br] species formed by (**1**) would be the least stable among all compounds, resulting in a faster release of the remaining carbonyl.

The coordination of the phenol group would be facilitated if the metal center were oxidated. Electrochemical experiments indicate that Mn(I) → Mn(II) oxidation potentials are directly influenced by the substituent groups. The dependency of the basicity of the phenol ring, as the coordination of the hydroxyl group itself, could be associated with an oxidation of the manganese(I) center after the release of the first carbonyl group, resulting in the coordination of the phenolic ring in order to decrease the electron deficiency of the manganese(II) center, and the consequent release of the remaining carbonyl groups due to π-backbonding weakening. Evidence of the spontaneous oxidation of the metal center was found in similar works [27,34,35].

The carbon monoxide releasing constants obtained from the pseudo-first-order kinetics (k_CO,old_) have a direct association with the Hammett sigma parameters (σ_p_). This is another indication that the phenol group could be coordinating the metal center after the CO release, once the greater the electron-withdrawing ability of the substituent group, the greater the CO release constant, because the coordination of the phenol group would directly influence the release of the other carbonyl ligands. 

On the other hand, a different tendency is observed when a new approach to determine CO release is used. It is observable that (**1**) remains as the compound with the highest k_CO,new_, while the other compounds deviate from the Hammett parameters. Compound (**2**) shows a lower k_CO_ than expected, which could be associated with the behavior of the compound during the whole CO release process. 

It is important to note that the k_CO,old_, being based in a pseudo-first-order kinetics, uses only the linear data from the absorbance exponential decay as a function of time. This indicates that, after the first CO release and the coordination of the phenol group, the intermediate species might be more stable when compared with the other analogous species from other compounds and, and due to that, have a lower rate constant, an effect that would not be observable using the old method alone.

Furthermore, the k_CO,new_ was expected to have a direct correlation with the absorption coefficient (ε) and with the quantum yield (Φ), since the CO release occurs in the excited state. It is important to note that MLCT transitions appear as shoulders in the UV-Vis, which means that **ε** is more of a range than a precise value itself. Regarding the quantum yield, it could be observed that the direct association is valid only for (**1**), while the other compounds presented a deviation from the tendency, indicating the possibility of side reactions and different photoproducts.

### 2.6. Myoglobin Assay

**Figure 11:**
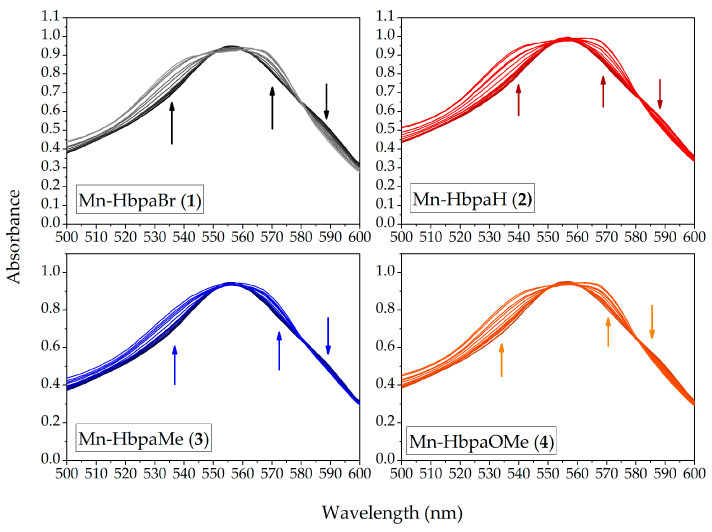
UV-Vis spectrum variation of deoxymyoglobin (Mb) upon CO release promoted by compounds (**1**)**–**(**4**) in PBS (0.1 mol L^−1^, pH = 7.4) during UV light excitation (λ_em_ = 395 ± 5 nm). (**1**)–(**4**) = 2.00 × 10^−5^ mol L^−1^.

CO release was also confirmed through the myoglobin interaction assay. Compounds (**1**)–(**4**) were diluted in PBS buffer solutions of hemoglobin in its reduced form (Mb). Upon exposure to light, the formation of MbCO was observed at 540 nm and 570 nm. The solutions were exposed to light for 210 min, until no further variation was observed. The spectral variation of myoglobin solutions is presented in Figure 11.

Table 5 presents the results obtained for CO released per molecule of photoCORM. The values indicate that around half of the carbonyl available was released from the manganese coordination sphere during the experiment. The necessary irradiation times are also longer due to the system’s conditions, in comparison with UV-Vis and IR CO release kinetics, once Mb is highly chromophore and hampers photon absorption by the metal carbonyl compounds. Furthermore, although the compounds have a similar range of CO release per mol, a direct tendency of the obtained values is not in accordance with UV-Vis and IR kinetic experiments, due to the presence of more variable parameters on the myoglobin assay.

## 3. Materials and Methods

All the reagents were acquired from commercial sources and used without previous purification, unless specified. The solvents used in all the syntheses and analyses were dried with molecular sieves (4 Å) for 72 h and degassed by argon flux or freeze pump using the Schlenk technique. The solutions containing the organometallic compounds were kept in the dark and wrapped in aluminum foil whenever possible.

Infrared spectra (FTIR, KBr pellets) were recorded on a PerkinElmer Spectrum 100 FT-IR in the range from 4000 cm^−1^ to 450 cm^−1^. UV-Vis spectra were recorded on a PerkinElmer Lambda 750 in quartz cuvettes, using spectroscopic-grade dichloromethane as solvent for the compounds. Molar conductivity of the organometallic compounds was performed on an MS Tecnopon mCA150 in spectroscopic-grade dichloromethane and acetonitrile. Cyclic voltammetry and square wave voltammetry were investigated on a BAS Epsilon potentiostat, using spectroscopic-grade dichloromethane as solvent under argon atmosphere. Tetrabutylammonium hexafluorophosphate (TBAPF_6_) with a concentration of 0.1 mol L^−1^ was used as supporting electrolyte, and the electrodes were Ag/Ag^+^ (reference) and platinum (work and auxiliary). The electrochemical potentials were referenced versus the ferrocene/ferrocenium (Fc/Fc^+^) redox pair. Elemental analysis was performed on a PerkinElmer 2400 Series II. ESI-MS positive ion mode measurements were performed on an Amazon spectrometer Ion Trap MS in spectroscopic-grade acetonitrile with an approximate concentration of 500 ppb and estimated flow of 180 μL min^−1^, with a capillary voltage of −400 V. ^1^H NMR spectra of the ligands were obtained on a Bruker AC-200 NMR spectrometer in CDCl_3_ solutions. For the organometallic compounds, ^1^H NMR spectra were carried out on a Bruker Ascend400 Varian FT-NMR 400 MHz using acetone-d_6_ (CD_3_)_2_CO as the solvent, at 25 °C. Chemical shifts were referenced to tetramethylsilane (TMS = 0.00 ppm), and *J* values are given in Hz. Stability of the complexes in solution was observed by UV-Vis and IR in spectroscopic dichloromethane and acetonitrile in the absence of light, where UV-Vis spectra were acquired at each hour for 24 h, and IR spectra were collected at the end of the experiment.

### 3.1. Single Crystal X-ray Diffraction 

Crystallographic data were collected on a Bruker APEX II Duo diffractometer with graphite monochromated Mo-Kα radiation (l = 0.71073 Å), at 150(2) K. Crystal structures were solved through direct methods and partially refined by the full-matrix least-squares on F^2^ [36]. Full crystallographic tables have been deposited with the Cambridge Crystallographic Data Centre at www.ccdc.cam.ac.uk accessed on 19 March 2023, with publication numbers CCDC 2246085-2246088.

### 3.2. CO Release Assay

Metal carbonyl compounds were dissolved in spectroscopic dichloromethane to final concentrations near 2.50 × 10^−4^ mol L^−1^ in quartz cuvettes. The quantum yield of CO release (Φ) and the CO photorelease constant *k_CO_* were calculated by monitoring the decrease in MLCT absorption bands through UV-Vis spectroscopy, at around 390 nm. Violet light-emitting diodes (LEDs) (λ_395_ = 395 ± 10 nm (photon flux = 1.13 × 10^−8^ Einstein s^−1^)) were employed to perform the CO release, where samples were perpendicularly irradiated 3.0 cm away from the light source. Ferrioxalate actinometry assay [37] was used to determine the photon flow of the light source. CO photorelease was also qualitatively observed through IR spectroscopy, where 100 µL of the compounds with the same concentration used on the UV-Vis experiment was added to a sealed solution cell and irradiated using the same light source. FTIR spectra were collected between determined intervals of light radiation until the disappearance of carbonyl bands between 2200 cm^−1^ and 1800 cm^−1^.

### 3.3. Myoglobin Assay

The myoglobin interaction assay was conducted based on a previous report [38]. A stock solution of myoglobin with a concentration of 6.33 × 10^−5^ mol L^−1^ (ε_540_ = 11,600 L mol^−1^ cm^−1^) was prepared in argon degassed Protein Buffer Saline (PBS) (0.1 mol L^−1^, pH 7.4) with an excess of sodium dithionite (85 mg). Compounds (**1**)–(**4**) were dissolved in spectroscopic-grade dichloromethane, resulting in concentrations of 1.00 × 10^−3^ mol L^−1^. 20 µL of the compound solutions was added to 980 µL of the hemoglobin solutions. Cuvettes were degassed and sealed with a Nujol layer and a Teflon cap to prevent carbon monoxide escape. Samples were irradiated identically to the CO release assay. Formation of MbCO was carried out until no significant spectral changes were observed.

### 3.4. Computational Studies

Geometry optimizations were carried out in a vacuum using the Orca 5.0.3 software package [39,40] at the density functional theory (DFT) level using the B3LYP functional [41,42] and the def2-TZVP basis set for Mn and Br. The def2-TZVP(-f) [43] with Grimme’s dispersion correction (D4) was used for the other atoms [44,45]. No imaginary frequencies were obtained, indicating that the optimized geometries correspond to the energy minima. Further structure details are provided in the Supporting Information section. The time-dependent density functional theory (TD-DFT) was used to simulate the electronic spectrum with the first 30 excitations using the same calculation protocol as before, including solvent (dichloromethane) correction using the conductor-like polarizable continuum model (CPCM) method with the SMD module [46]. Images were rendered using the UCSF Chimera Program [47].

Energy decomposition analysis (EDA) and natural orbitals for chemical valence (EDA-NOCV) [48] were performed using the ADF2021 software [49] with the B3LYP functional, the TZ2P basis set [50,51], and dispersion Grimme3 BJDAMP [52,53]. In all cases, the two fragments were considered neutral without residual charges. The EDA scheme decomposes the interaction energy (ΔE_int_) into the sum of four physical significant terms: Pauli repulsion (ΔE_Pauli_), electrostatic interaction (ΔE_elstat_), orbital interaction (ΔE_oi_), and the dispersion (ΔE_disp_), according to Equation (1).

ΔE_int_ = ΔE_Pauli_ + ΔE_elstat_ + ΔE_oi_ + ΔE_disp_ (1)

The term ΔE_Pauli_ is responsible for steric repulsion, consisting of the destabilizing interactions between occupied orbitals of the fragments. The ΔE_elstat_ refers to the classical electrostatic interaction between the undisturbed charge distributions of the prepared fragments. The dispersion contributions are considered by the term ΔE_disp_. Orbital interactions represent the polarization and the charge transfer between occupied orbitals on one fragment and the empty orbitals of another. They can be separated into the sum of different compositions (Equation (2)). In this work, two interactions represent around 96% of the ΔE_oi_, which is the sum of the σ interaction (ΔE_oi-σ_) and the π interactions (ΔE_oi-π_). The rest (ΔE_oi-rest_) is composed of interactions that have small contributions (less than −2.0 kcal mol^−1^ each, with a maximum sum of −3.9 kcal mol^−1^).

ΔE_oi_ = ΔE_oi-σ_ + ΔE_oi-π_ + ΔE_oi-rest_ (2)

### 3.5. Synthesis of the Compounds

The general synthesis of the ligands **Hbpa-R** (R = H, Me and OMe) was based on previous reports [23,24]. In a methanolic solution of the respective salicylaldehyde (30 mmol), 2-aminomethylpyridine was added (30 mmol, 108 14 g mol^−1^, 1.049 g mL^−1^) under ice bath and stirring. The reaction mixture was kept under stirring for 3 h, and then sodium borohydride (30 mmol, 37.82 g mol^−1^) was slowly added to the reaction mixture over an hour. The pH was adjusted to 6.0 with diluted HCl (3.5 mol L^−1^), and the solvent was removed under low pressure. The reminiscent oil was dissolved in 25 mL of CH_2_Cl_2_ and washed with a saturated solution of sodium bicarbonate (8 × 40 mL). The organic phase was dried with anhydrous sodium sulfate and filtered, and then the solvent was removed using a rotary evaporator, forming a yellow oil.

#### 3.5.1. N-(2-Pyridylmethyl)(2-hydroxy-5-bromobenzyl)amine—HbpaBr

The synthesis of HbpaBr was based on a previous report [54]. Methanol was substituted for toluene. A Dean–Stark apparatus was coupled to the reaction system and the reaction was kept under reflux for 1 h, and then overnight at room temperature. After obtaining a yellow oil, it was dissolved in dichloromethane and recrystallized, resulting in greenish-yellow crystals, 6.24 g, 71%, 293.16 g mol^−1^; IR (FTIR, KBr) ν/cm^−1^ 3292 (NH), 3058–2840 (CH_ar_ and CH_aliphatic_), 1594-1479 (CC and CN), 1266 (CO), 755 (CH_ar_); UV-Vis (CH_2_Cl_2_) λ_max_/nm (ε/L mol^−1^ cm^−1^) 261 (2254), ~289 (1457); ^1^H NMR δH/ppm (200 MHz; CDCl_3_; TMS): 4.01 (s, 2H_CH2_), 4.11 (s, 2H_CH2_), 6.92 (d, *J* = 9.09 Hz, 1H_ar_), 7.25–7.30 (m, 2H_ar_), 7.71 (t, *J* = 7.42 Hz, 1H_ar_), 7.92–8.01 (m, 1H_ar_), 8.10 (d, *J* = 9.09 Hz, 1H_ar_), 8.60 (d, *J* = 4.24 Hz, 1H_py_).

#### 3.5.2. N-(2-Pyridylmethyl)(2-hydroxybenzyl)amine—HbpaH

After the formation of the yellow oil, it was dissolved in dichloromethane and recrystallized, resulting in a white solid (4.28 g, 67%, 214.27 g mol^−1^); IR (FTIR, KBr) ν/cm^−1^ 3428 (OH), 3264 (NH), 3042–2860 (CH_ar_ and CH_aliphatic_), 1594–1430 (CC and CN), 1257 (CO), 748 (CH_ar_); UV-Vis (CH_2_Cl_2_) λ_max_/nm (ε/L mol^−1^ cm^−1^) 263 (2018), ~280 (1250); ^1^H NMR δH/ppm (200 MHz; CDCl_3_; TMS): 3.93 (s, 2H_CH2_), 4.01 (s, 2H_CH2_), 6.75-6.89 (m, 2H_ar_), 6.98 (d, *J* = 7.10 Hz, 1H_ar_), 7.15–7.25 (m, 3H_ar_), 7.67 (dt, *J* = 1.84 Hz; 7.84 Hz; 1H_ar_), 8.59 (d, *J* = 4.02 Hz, 1H_py_).

#### 3.5.3. N-(2-Pyridylmethyl)(2-hydroxy-5-methylbenzyl)amine—HbpaMe

Yellow oil, 6.66 g, 96%, 231.27 g mol^−1^; IR (FTIR, KBr) ν/cm^−1^ 3290 (NH), 3053–2859 (CH_ar_ and CH_alif_), 1594–1435 (CC and CN), 1261 (CO), 769 (CH_ar_); UV-Vis (CH_2_Cl_2_) λ_max_/nm (ε/L mol^−1^ cm^−1^) 262 (2481), ~284 (1917); ^1^H NMR δH/ppm (200 MHz; CDCl_3_; TMS): 2.24 (s, 3H_CH3_), 3.95 (s, 2H_CH2_), 4.00 (s, 2H_CH2_), 6.76-6.80 (m, 2H_ar_), 6.98 (d, *J* = 8.16 Hz; 1H_ar_), 7.18–7.28 (m, 2H_ar_), 7.67 (dt, *J* = 1.35 Hz; 7.60 Hz; 1H_ar_), 8,57 (d, 4.02 Hz, 1H_py_).

#### 3.5.4. N-(2-Pyridylmethyl)(2-hydroxy-5-methoxybenzyl)amine—HbpaOMe

Orange oil, 6.08 g, 82%, 247.27 g mol^−1^; IR (FTIR, KBr) ν/cm^−1^ 3295 (NH), 3053–2833 (CH_ar_ and CH_aliphatic_), 1593–1434 (CC and CN), 1253 (CO), 763 (CH_ar_); UV-Vis (CH_2_Cl_2_) λ_max_/nm (ε/L mol^−1^ cm^−1^) 262 (3143), ~298 (3467); ^1^H NMR δH/ppm (200 MHz; CDCl_3_; TMS): 3.74 (s, 3H_CH3_), 3.92 (s, 2H_CH2_), 3.97 (s, 2H_CH2_), 6.54–6.58 (m, 1H_ar_), 6.71–6.82 (m, 2H_ar_), 7.16–7.26 (m, 2H_ar_), 7.66 (dt, *J* = 1.54 Hz; 7.65 Hz; 1H_ar_), 8.58 (d; *J* = 3.54 Hz; 1H_py_).

The photoCORMs (**1**)–(**4**) were synthesized using Schlenk techniques. A frozen solution of CH_2_Cl_2_ (15 mL) containing the respective ligand (0.4 mmol) was degassed repeatedly before the addition of [MnBr(CO)_5_] (109.96 mg, 0.4 mmol). After reaching room temperature, the solution was refluxed for 12 h under argon atmosphere in the dark. The four compounds synthesized resulted in a yellow solution. The solution was then dried at 30 °C in a laboratory kiln, leading to the formation of yellow precipitates. The precipitates were then washed with cold hexane three times and recrystallized in a 1:1 CH_2_Cl_2_/hexane, resulting in yellow crystals.

#### 3.5.5. [MnBr(HbpaBr)(CO)_3_] (**1**)

Yellow crystals, 159.44 mg, 78%, 511.03 g mol^−1^; IR (FTIR, KBr) ν/cm^−1^ 3400 (OH), 3222 (NH), 2025 (CO_sym_), 1940 (CO_asym_), 1907 (CO_asym_), 1607–1495 (CC and CN), 1261 (CO), 771 (CH_ar_); UV-Vis (CH_2_Cl_2_) λ_max_/nm (ε/L mol^−1^ cm^−1^) 284 (4127), 321sh (2128), 379sh (1600); ESI-MS *m/z* 430.86 (100, M-Br^-^); Elemental Analysis Found: C, 42.23; H, 3.59; N, 5.70%. Calc. for C_16_H_12_Br_2_MnN_2_O_4_·1 CH_2_Cl_2_: C, 42.21; H, 3.63; N, 5.79%. ^1^H NMR δH/ppm (400 MHz; (CD_3_)_2_CO; TMS): 3.90 (b, 1H_OH_), 4.10-4.26 (m, 2H_CH2_), 4.31 (dd, *J* = 12.80 Hz, 1H_CH2_), 4.78 (d, *J* = 12.80 Hz, 1H_CH2_), 6.96 (d, *J* = 6.10 Hz, 1H_ar_), 7.37–7.67 (m, 4H_ar_), 7.93 (s, 1H_ar_), 8.99 (s, 1H_ar_), 9.65 (1H_NH_). Crystal data for (**2**) (M = 512.04 g mol^−1^): triclinic, space group P-1, a = 7.8714(7) Å, b = 8.5609(7) Å, c = 14.1438(12) Å, α = 97.177(2)°, β = 105.699(2)°, γ = 97.734(2)°, V = 896.10(13) Å^3^, Z = 2, D_calc_ = 1.898 Mg/m^3^, 15003 reflections measured (1.517° ≤ 2Θ ≤ 30.577°), 5509 unique (R_int_ = 0.0258). The final R1 was 0.0279 (I > 2σ(I)), and wR2 was 0.0587 (all data).

#### 3.5.6. [MnBr(Hbpa)(CO)_3_] (**2**)

Yellow crystals, 153.83 mg, 89%, 432.13 g mol^−1^; IR (FTIR, KBr) ν/cm^−1^ 3413 (OH), 3231 (NH), 2025 (CO_sym_), 1930 (CO_asym_), 1914 (CO_asym_), 1610–1458 (CC and CN), 1260 (CO), 760 (CH_ar_); UV-Vis (CH_2_Cl_2_) λ_max_/nm (ε/L mol^−1^ cm^−1^) 273 (6524), 323sh (3716), 379sh (2900); ESI-MS *m/z* 352.93 (100, M-Br^-^); Elemental Analysis Found: C, 44.43; H, 3.33; N, 6.32%. Calc. For C_16_H_13_BrMnN_2_O_4_: C44.47; H, 3.03; N, 6.48%. ^1^H NMR δH/ppm (400 MHz; (CD_3_)_2_CO; TMS): 4.07–4.15 (m, 2H_CH2_), 4.26 (dd, *J* = 5.39 Hz, 1H_CH2_), 4.76–4.82 (m, 1H_CH2_), 6.91 (dt, *J* = 1.05 Hz, 1H_ar_), 6.99 (d, *J* = 8.08 Hz, 1H_ar_), 7.27 (dt, *J* = 1.61 Hz; *J* = 7.78 Hz, 1H_ar_), 7.40 (dd, *J* = 1.61 Hz, 1H_ar_), 7.50–7.54 (m, 2H_ar_), 7.92 (dt, *J* = 1.61 Hz, 1H_ar_), 8.98 (dd, *J* = 1.61 Hz, 1H_ar_), 9.25 (1H_NH_); Crystal data for (**1**) (M = 433.14 g mol^−1^): monoclinic, space group P21/n, a = 13.5543(8) Å, b = 9.7453(6) Å, c = 26.0146(16) Å, β = 93.5850(10)°, V = 3429.6(4) Å^3^, Z = 8, D_calc_ = 1.678 Mg/m^3^, 38127 reflections measured (1.569° ≤ 2Θ ≤ 30.145°), 10119 unique (R_int_ = 0.0186). The final R1 was 0.0272 (I > 2σ(I)), and wR2 was 0.0624 (all data).

#### 3.5.7. [MnBr(HBPAMe)(CO)_3_] (**3**)

Yellow crystals, 162.40 mg, 91%, 446.16 g mol^−1^; IR (FTIR, KBr) ν/cm^−1^ 3420 (OH), 3224 (NH), 2027 (CO_sym_), 1940 (CO_asym_), 1907 (Coa_sym_), 1608–1447 (CC and CN), 1261 (CO), 768 (CH_ar_); UV-Vis (CH_2_Cl_2_) λ_max_/nm (ε/L mol^−1^ cm^−1^) 281 (6021), 323sh (3030), 379sh (2400); ESI-MS *m/z* 366.96 (100, M-Br^-^); Elemental Analysis Found: C, 44.33; H, 3.64; N, 5.95%. Calc. for C_17_H_15_BrMnN_2_O_4_·0.8 H_2_O: C, 44.33; H, 3.63; N, 6.08%. ^1^H NMR δH/ppm (400 MHz; (CD_3_)_2_CO; TMS): 2.27 (s, 3H_CH3_), 4.0 (b, 1H_OH_), 4.02–4.14 (m, 2H_CH2_), 4.23–4.30 (m, 1H_CH2_), 4.75 (d, *J* = 13.30 Hz, 1H_CH2_), 4.82 (b, 1H_NH_), 6.87 (d, *J* = 8.03 Hz, 1H_ar_), 7.07 (m, 1H_ar_), 7.20 (m, 1H_ar_), 7.49–7.54 (m, 2H_ar_), 7.92 (t, *J* = 7.18 Hz, 1H_ar_), 8.91–9.00 (m, 1H_ar_, 1H_NH_). Crystal data for (**3**) (M = 447.17 g mol^−1^): triclinic, space group P-1, a = 7.7748(10) Å, b = 8.4882(11) Å, c = 14.1772(18) Å, α = 77.426(2)°, β = 75.239(2)°, γ = 84.486(2)°, V = 882.2(2) Å3, Z = 2, D_calc_ = 1.683 Mg/m^3^, 20422 reflections measured (2.461° ≤ 2Θ ≤ 33.229°), 6734 unique (R_int_ = 0.0205). The final R1 was 0.0299 (I > 2σ(I)), and wR2 was 0.0735 (all data).

#### 3.5.8. [MnBr(HBPAOMe)(CO)_3_] (**4**)

Yellow crystals, 164.52 mg, 89%, 462.16 g mol^−1^; IR (FTIR, KBr) ν/cm^−1^ 3434 (OH), 3222 (NH), 2025 (CO_sym_), 1928 (CO_asym_), 1911 (CO_asym_), 1611–1434 (CC and CN), 1265 (C-O), 764 (C-Har); UV-Vis (CH_2_Cl_2_) λ_max_/nm (ε/L mol^−1^ cm^−1^) 295 (5926), 329sh (~2300), 379sh (~1800); ESI-MS *m/z* 382.96 (100, M-Br^-^); Elemental Analysis Found: C, 42.23; H, 3.59; N, 5.70%. Calc. for C_17_H_15_BrMnN_2_O_5_·1.2 H_2_O: C, 42.21; H, 3.63; N, 5.79%. ^1^H NMR δH/ppm (400 MHz; (CD_3_)_2_CO; TMS): 3.76 (s, 3H_CH3_), 4.04–4.19 (m, 2H_CH2_), 4.23–4.33 (m, 1H_CH2_), 4.75 (d, *J* = 12.80 Hz, 1H_CH2_), 6.05 (b, 1H_OH_), 6.80–6.94 (m, 2H_ar_), 7.04 (s, 1H_ar_), 7.53 (s, 1H_ar_), 7.92 (s, 1H_ar_), 8.82 (1H_NH_), 8.98 (s, 1H_ar_). Crystal data for (**4**) (M = 463.17 g mol^−1^): triclinic, space group P-1, a = 7.5412(9) Å, b = 13.8743(16) Å, c = 18.566(2) Å, α = 79.549(2)°, β = 87.930(2)°, γ = 77.108(2)°, V = 1862.1(4) Å3, Z = 4, D_calc_ = 1.652 Mg/m^3^, 40852 reflections measured (1.726° ≤ 2Θ ≤ 32.822°), 13728 unique (R_int_ = 0.0470). The final R1 was 0.0491 (I > 2σ(I)), and wR2 was 0.1187 (all data).

## 4. Conclusions

A series of mononuclear Mn(I) complexes containing bioinspired tridentate ligands with a *N,O*-donor set and *p*-substituted phenols were synthesized and fully characterized through structural and physicochemical methods. All complexes presented an octahedral geometry where the carbonyl ligands were coordinated in a *facial* arrangement.

A thorough description of the electronic structure of all complexes were carried out considering the DFT approach through which frontier orbitals were calculated, as well as the energy decomposition analysis through natural orbitals for chemical valence (EDA-NOCV), which allowed us to rationalize all the contributions of each moiety present (amines, pyridines, CO, and bromide) in the formed chemical bonds.

The stability of these compounds was observed using UV-Vis and molar conductivity in dichloromethane, showing a partial ligand substitution (Br^-^ by solvent) when acetonitrile was employed. CO release rates were quantitatively determined through UV-Vis, in which complex [MnBr(HbpaBr)(CO)_3_] (**1**) presented the highest value (2.36 ± 0.11 × 10^−3^ s^−1^) and quantum yield (0.031 ± 0.0002). The CO release was also confirmed using myoglobin assays, with all complexes presenting similar results.

Finally, a common viable CO-release mechanism for all complexes was proposed, according to which, after light irradiation, a CO group was promptly released, followed by the metal center’s oxidation, suggesting the consequent coordination of the phenol group. Further studies in vitro will be performed with compounds (**1**)–(**4**) to assess their cytotoxicity.

## Figures and Tables

**Table 1:** Selected bond lengths (Å) for compounds (**1**)–(**4**).

Bond	Compound			
	(1)	(2a)	(3)	(4a)
Mn–C(1)	1.815(2)	1.816(2)	1.8214(17)	1.814(3)
Mn–C(2)	1.811(2)	1.8119(19)	1.8154(17)	1.816(3)
Mn–C(3)	1.802(2)	1.7948(19)	1.7973(17)	1.798(3)
Mn–N(*sp*^3^)	2.0856(16)	2.0886(15)	2.0860(14)	2.089(2)
Mn–N(*sp*^2^)	2.0590(15)	2.0525(14)	2.0610(14)	2.052(2)
Mn–Br	2.5254(4)	2.5273(3)	2.5258(3)	2.5320(5)

**Table 2:** Summarized Mn**^I/II^** oxidation potentials for compounds (**1**)–(**4**) in dichloromethane under argon atmosphere and λ_max_ of absorption bands in UV-Vis for compounds (**1**)–(**4**) in dichloromethane.

Compound	E_p_^MnI/II^ (V) vs. Fc/Fc^+^	UV-Vis λ_max_, nm (ε, L mol^−1^ cm^−1^)
(**1**)	0.681	284 (4127), 321sh (~2128), 379sh (~1600)
(**2**)	0.741	273 (6524), 323sh (~3716), 379sh (~2900)
(**3**)	0.667	281 (6021), 323sh (~3030), 379sh (~2400)
(**4**)	0.643	295 (5926), 329sh (~2300), 379sh (~1800)

CV experimental conditions: 0.1 mol L^−1^ TBAPF_6_ as the supporting electrolyte, Ag/AgCl as the reference electrode, and platinum as the work and auxiliary electrodes. Potentials are referenced to the Fc/Fc^+^ couple (E_1/2_ = 0.405 V vs. Ag/AgCl in dichloromethane). sh = shoulder.

**Table 3:** Energy decomposition analysis in kcal mol^−1^ and, in parentheses, the percentage for the contribution of the attractive interactions² and orbital interactions³, where 1-CO is *trans* to bromide, 2-CO is *trans* to pyridyl group, and 3-CO *trans* to amino group.

	**^1^ ΔEint**	**ΔEPauli**	**^2^ ΔEelstat**	**^2^ ΔEdisp**	**^2^ ΔEoi**	**^3^ ΔEoi-σ**	**^3^ ΔEoi-π**	**^3^ ΔEoi-rest**
(**1**)								
1-CO	−44.93	168.27	−114.24 (53.6)	−2.59 (1.2)	−96.38 (45.2)	−40.42 (41.9)	−52.98 (55.0)	−2.98 (3.1)
2-CO	−36.64	166.95	−112.41 (55.2)	−2.38 (1.2)	−88.80 (43.6)	−37.74 (42.5)	−47.61 (53.6)	−3.45 (3.9)
3-CO	−37.15	167.67	−112.96 (55.2)	−2.07 (1.0)	−89.78 (43.8)	−38.31 (42.7)	−48.09 (53.6)	−3.38 (3.8)
(**2a**)								
1-CO	−45.16	169.01	−114.78 (53.6)	−2.55 (1.2)	−96.83 (45.2)	−40.75 (42.1)	−53.09 (54.8)	−2.99 (3.1)
2-CO	−36.50	168.41	−113.24 (55.3)	−2.37 (1.2)	−89.31 (43.6)	−38.16 (42.7)	−47.69 (53.4)	−3.46 (3.9)
3-CO	−37.10	167.72	−112.99 (55.2)	−2.10 (1.0)	−89.73 (43.8)	−38.11 (42.5)	−48.20 (53.7)	−3.42 (3.8)
(**2b**)								
1-CO	−45.03	167.86	−113.99 (53.5)	−2.60 (1.2)	−96.31 (45.2)	−40.25 (41.8)	−53.08 (55.1)	−2.98 (3.1)
2-CO	−36.58	166.79	−112.34 (55.2)	−2.36 (1.2)	−88.67 (43.6)	−37.78 (42.6)	−47.46 (53.5)	−3.43 (3.9)
3-CO	−37.28	167.45	−112.90 (55.1)	−2.07 (1.0)	−89.75 (43.8)	−38.05 (42.4)	−48.37 (53.9)	−3.33 (3.7)
(**3**)								
1-CO	−45.06	167.73	−114.64 (53.6)	−2.58 (1.2)	−96.73 (45.2)	−40.64 (42.0)	−53.10 (54.9)	−2.99 (3.1)
2-CO	−36.58	166.11	−112.81 (55.3)	−2.39 (1.2)	−89.07 (43.6)	−37.84 (42.5)	−47.75 (53.6)	−3.48 (3.9)
3-CO	−37.14	166.72	−113.01 (55.1)	−2.07 (1.0)	−89.76 (43.8)	−38.18 (42.5)	−48.13 (53.6)	−3.45 (3.8)
(**4a**)								
1-CO	−45.06	169.66	−113.91 (53.5)	−2.61 (1.2)	-96.27 (45.2)	−40.94 (42.5)	−53.09 (55.1)	−2.24 (3.8)
2-CO	−36.57	167.39	−112.03 (55.3)	−2.33 (1.1)	-88.32 (43.6)	−37.95 (43.0)	−47.40 (53.7)	−2.97 (4.3)
3-CO	−37.43	167.46	−112.46 (55.1)	−2.08 (1.0)	-89.61 (43.9)	−38.05 (42.5)	−48.33 (53.9)	−3.23 (3.9)
(**4b**)								
1-CO	−45.01	168.89	−115.05 (53.6)	−2.61 (1.2)	-97.01 (45.2)	−40.22 (41.5)	−53.06 (54.7)	−3.73 (2.3)
2-CO	−36.44	167.69	−112.70 (55.3)	−2.37 (1.2)	-88.75 (43.5)	−37.74 (42.5)	−47.17 (53.1)	−3.84 (3.4)
3-CO	−37.28	167.7	−112.89 (55.1)	−2.09 (1.0)	-89.76 (43.8)	−37.96 (42.3)	−48.30 (53.8)	−3.50 (3.6)

^1^ ΔE_int_ = ΔE_Pauli_ + ΔE_elstat_ + ΔE_oi_ + ΔE_disp._^2^ Contribution for total attractive interactions in parentheses (ΔE_elstat_ + ΔE_oi_ + ΔE_disp_). ^3^ Contribution for total orbital interactions in parentheses (ΔE_oi_ = ΔE_oi-σ_ + ΔE_oi-π_ + ΔE_oi-rest_).

**Table 4:** Summarized CO release constant rates (k_CO_), Hammett sigma constants (σ_p_), MLCT absorption coefficient (ε), half-life times (t_1__/2_), and quantum yield (Φ) for compounds (**1**)–(**4**).

Compound	k_CO,old_ (10^−3^ s^−1^)	σ_p_	k_CO,new_ (10^−3^ s^−1^)	ε (L mol^−1^ cm^−1^)	t_1/2_ (s)	Φ
(**1**)	2.36 ± 0.11	0.23	2.37 ± 0.09	~1600	264.85 ± 9.71	0.031 ± 0.0002
(**2**)	1.77 ± 0.02	0.00	1.90 ± 0.001	~2900	243.63 ± 10.98	0.019 ± 0.0002
(**3**)	1.60 ± 0.02	−0.17	2.03 ± 0.04	~2400	266.60 ± 3.78	0.011 ± 0.0002
(**4**)	1.24 ± 0.04	−0.27	2.07 ± 0.04	~1800	265.22 ± 1.18	0.017 ± 0.0001

**Table 5:** Equivalent amount of CO released per mol of the compounds (**1**)–(**4**) during UV light irradiation (λ_em_ = 395 ± 5 nm).

Compound	Amount of CO Released
(**1**)	1.544 ± 0.033
(**2**)	1.827 ± 0.093
(**3**)	1.248 ± 0.068
(**4**)	1.591 ± 0.042

## Data Availability

No applicable.

## Supplementary Materials

The following supporting information can be downloaded at: https://www.mdpi.com/article/10.3390/molecules28083439/s1, Figures S1–S6: IR, UV-Vis, and ^1^H NMR spectra of **Hbpa-R** ligands; Figure S7: Crystal structures obtained for compounds (**2**) and (**4**); Tables S1–S8: Selected crystallographic data for compounds (**1**)–(**4**); Figures S8–S9: IR spectra and SWV of compounds (**1**)–(**4**); Figure S10: CV of (**4**) measured in both scan directions; Figures S11–S15: ESI-MS(+) and ^1^H NMR spectra of compounds (**1**)–(**4**); Figures S16–S24: Comparison and relation between the calculated and experimental IR and UV-Vis spectra of compounds (**1**)–(**4**); Table S9: Data for transitions (TD-DFT) of compounds (**1**)–(**4**); Figure S25: Representation of the CO fragments used in the energy decomposition analysis (EDA) of compounds (**1**)–(**4**); Table S10: The most relevant density flow channel with their respective energies and CT estimation of compounds (**1**)–(**4**); Figures S26–S27: Representation of the density flow channel for the 2-CO and 3-CO fragments of compound **2a** and of the orbitals of the fragments contributions; Figures S28–S31: Spectral accompaniment of compounds (**1**)–(**4**) in DCM and MeCN by UV-Vis at each hour over 24 h; Figures S32–S34: Changes in UV-Vis spectra of compounds (**2**)–(**4**) during UV light irradiation; Equations used to determine k_CO_^new^, t_1/2_, and k_CO_^old^; Cartesian coordinates of optimized structures.
