# Supplementary material for: Spectroscopical and Molecular Studies of Four Manganese(I) PhotoCORMs with Bioinspired Ligands Containing Non-Coordinated Phenol Groups
Source: Molecules. 2023 Apr 13;28(8):3439. doi: 10.3390/molecules28083439 (PMC10144837; doi:10.3390/molecules28083439)
Supplement: Supplementary file 1 [file molecules-28-03439-s001.zip › molecules-2325453-supplementary.pdf]

Supplementary Materials

Spectroscopical and molecular studies of four manganese(I) photoCORMs with bioinspired ligands containing non-coordinated phenol groups

Matheus S. S. Paqui [1], Vinícius A. Glitz [1], Daniele C. Durigon [1], André L. Amorim [1], Giovanni F. Caramori [1], Renato L. T. Parreira [2], Adailton J. Bortoluzzi [1], Fernando R. Xavier [3,*] and Rosely A. Peralta [1,*]

1 Departamento de Química, Universidade Federal de Santa Catarina (*UFSC*), 88040-900 Florianópolis-SC, Brazil
2 Núcleo de Pesquisas em Ciências Exatas e Tecnológicas, Universidade de Franca, 14404-600 Franca-SP, Brazil
3 Universidade do Estado de Santa Catarina (*UDESC*), Campus Joinville, 89219-710 Joinville-SC, Brazil
* Correspondence: fernando.xavier@udesc.br; Tel.: +55(47)3481-7654

Contents

Figure S1: IR spectra of **Hbpa-R** ligands.
Figure S2: UV-Vis spectra of **Hbpa-R** ligands.
Figures S3-S6: ^{1}H NMR spectra of **Hbpa-R** ligands.
Tables S1-S8: Selected crystallographic data for compounds **(1)-(4)**.
Figure S7: Isomers and conformers crystal structures obtained for compounds **(2)** and **(4)**.
Figure S8: IR spectra of compounds **(1)-(4)**.
Figure S9: Square Wave Voltammetry of compounds **(1)-(4).**
Figure S10: Cyclic Voltammetry of **(4)** measured in both scan directions.
Figure S11: ESI-MS(+) spectra of compounds **(1)-(4)**.
Figures S12-S15: ^{1}H NMR spectra of compounds **(1)-(4).**
Figures S16-S19: Comparison between the calculated and experimental IR spectra of compounds **(1)-(4).**
Figures S20: Relation between experimental and calculated CO stretching values of compounds **(1)-(4).**
Figures S21-S24: Comparison between the calculated and experimental UV-Vis spectra of compounds **(1)-(4).**
Table S9: Data for transitions (TD-DFT) of compounds **(1)-(4).**
Figure S25: Representation of the 1-CO, 2-CO and 3-CO fragments used in the energy decomposition analysis (EDA) of compounds **(1)-(4).**
Table S10: The most relevant density flow channel with their respective energies and charge transfer estimation of compounds **(1)-(4).**
Figures S26-S27: Representation of the density flow channel for the 2-CO and 3-CO fragments of compound **2a** and also of the orbitals of the fragments that more contribute to its formation.
Figure S28: Spectral accompaniment of compounds **(1)-(4)** in DCM by UV-Vis at each hour over 24h.
Figures S29-S31: Spectral accompaniment of compounds **(2)-(4)** in CH_3CN by UV-Vis at each hour over 24h.
Figures S32-S34: Changes in UV-Vis spectra of compounds **(2)-(4)** during UV light irradiation.
Equations used to determine k_{CO}, $t_{1/2}$ and k_{CO}^{old}.
Cartesian coordinates of optimized structures

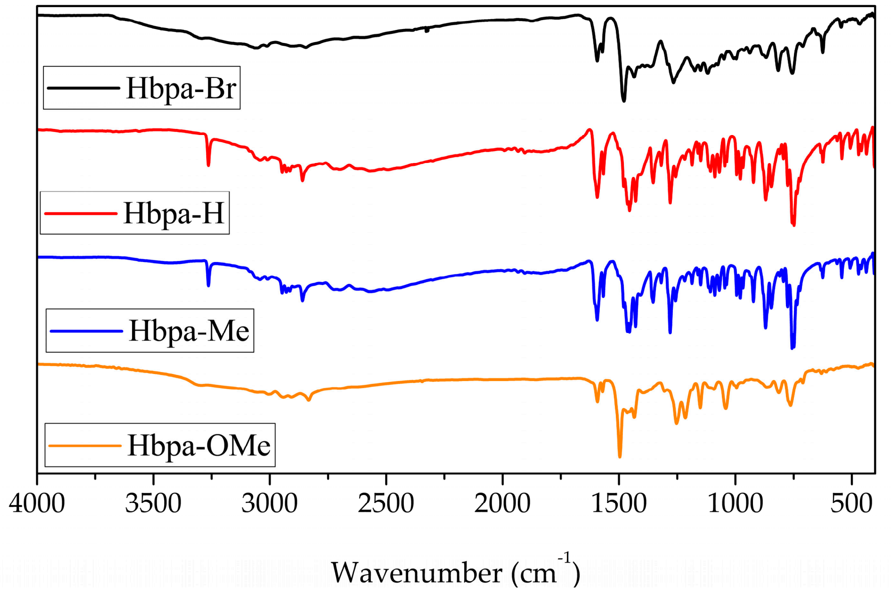

Figure S1. IR spectra of **Hbpa-R** ligands (FTIR, KBr pellets) in the range of 4000 to 450 cm^{-1}.

IR spectra of **Hbpa-R** ligands is in direct accordance with literature [23,24]. Given the homologous structure of the compounds, IR absorption bands are also very similar. The absence of carbonyl stretching bands around 1750 cm^{-1} indicates that the aldehyde precursor was completely consumed or successfully purified.

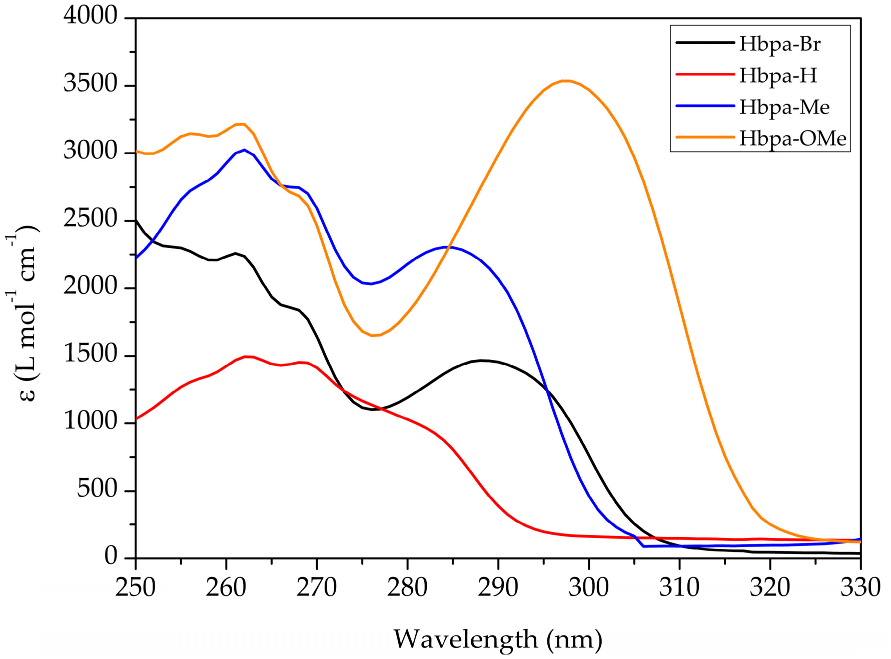

Figure S2. UV-Vis spectra of **Hbpa-R** ligands in dichloromethane.

The most energetic absorption band around 260 nm is associated with pπ*← pπ electronic transitions on the pyridine, while the absorption band above 280 nm corresponds to π*← n electronic transitions on the phenolic ring [23]. The ligands electronic spectra already present the tendency involving the electronic influence of the substituent group, where **Hbpa-Br** has a more similar behavior to **Hbpa-Me**.

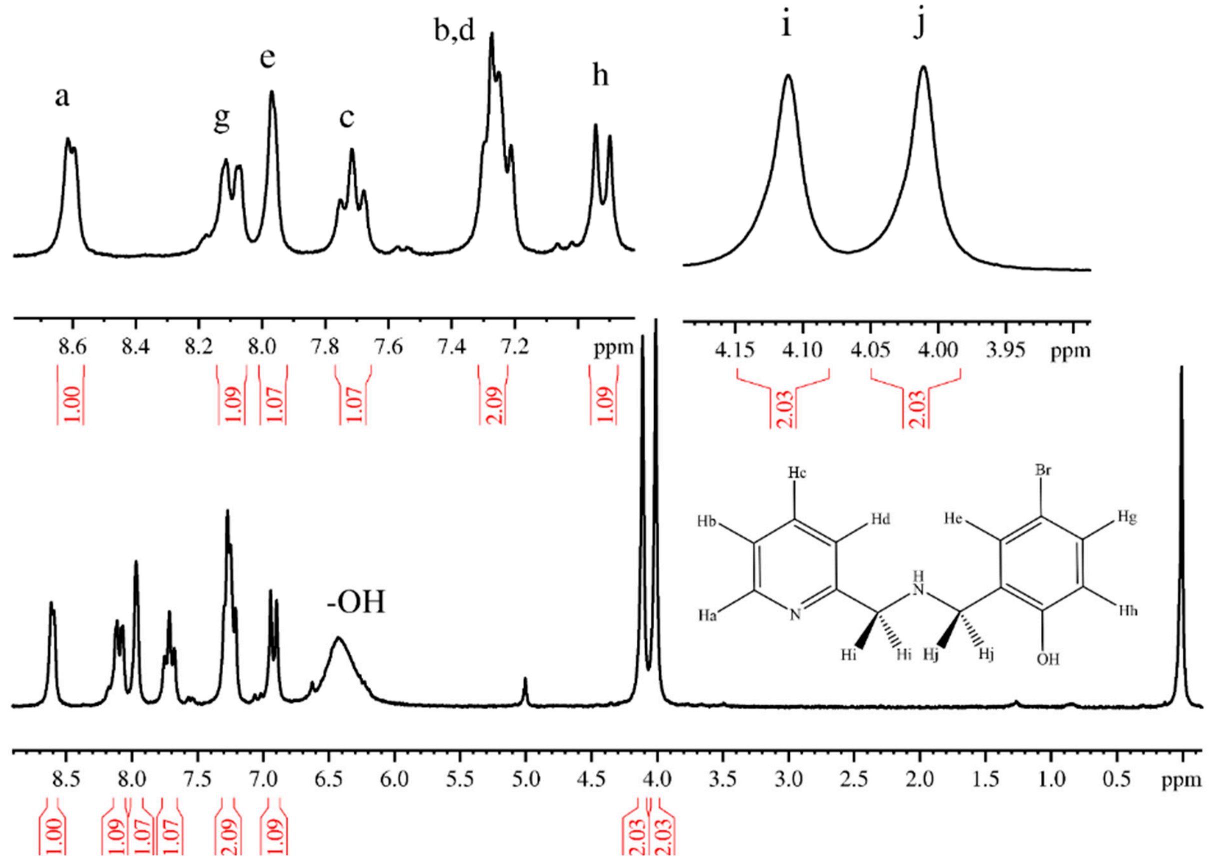

Figure S3. ^{1}H NMR (200 MHz, TMS= 0.00 ppm) spectrum of **Hbpa-Br** in $CDCl_3$.

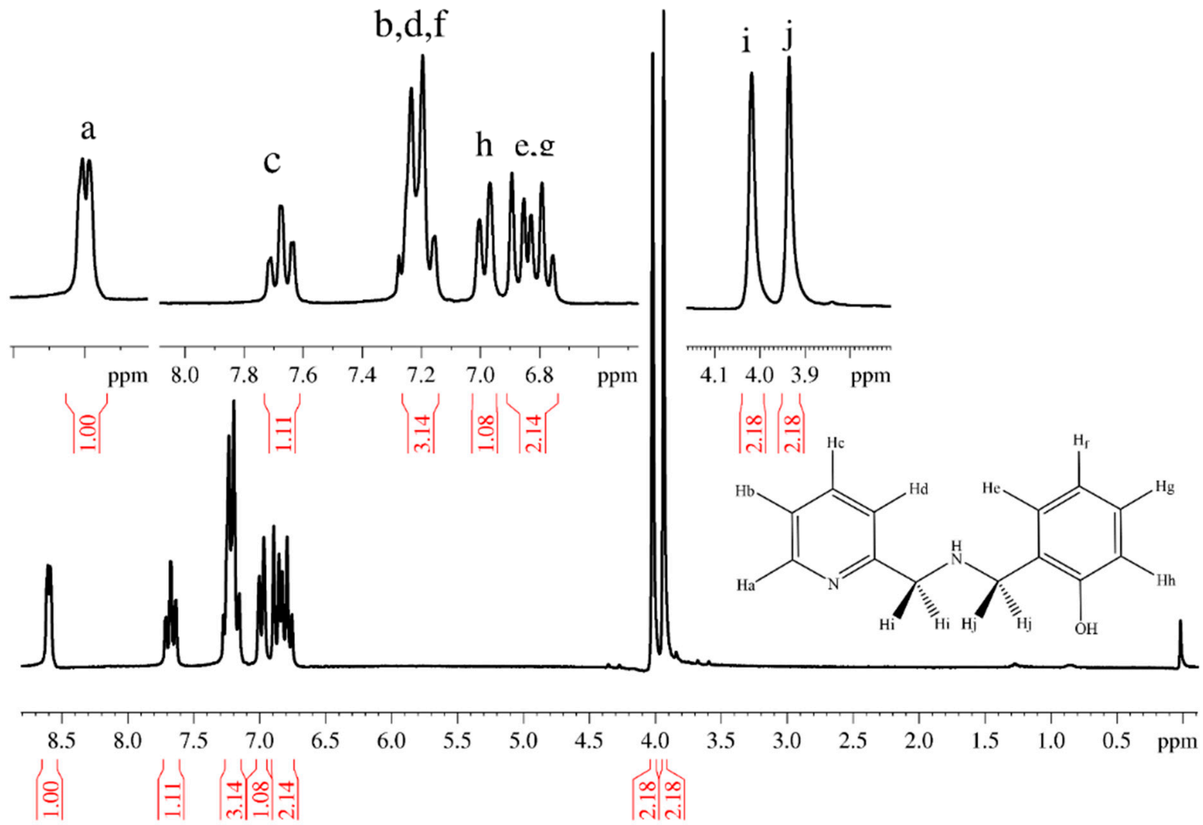

.

Figure S4. ^{1}H NMR (200 MHz, TMS= 0.00 ppm) spectrum of **Hbpa-H** in $CDCl_3$.

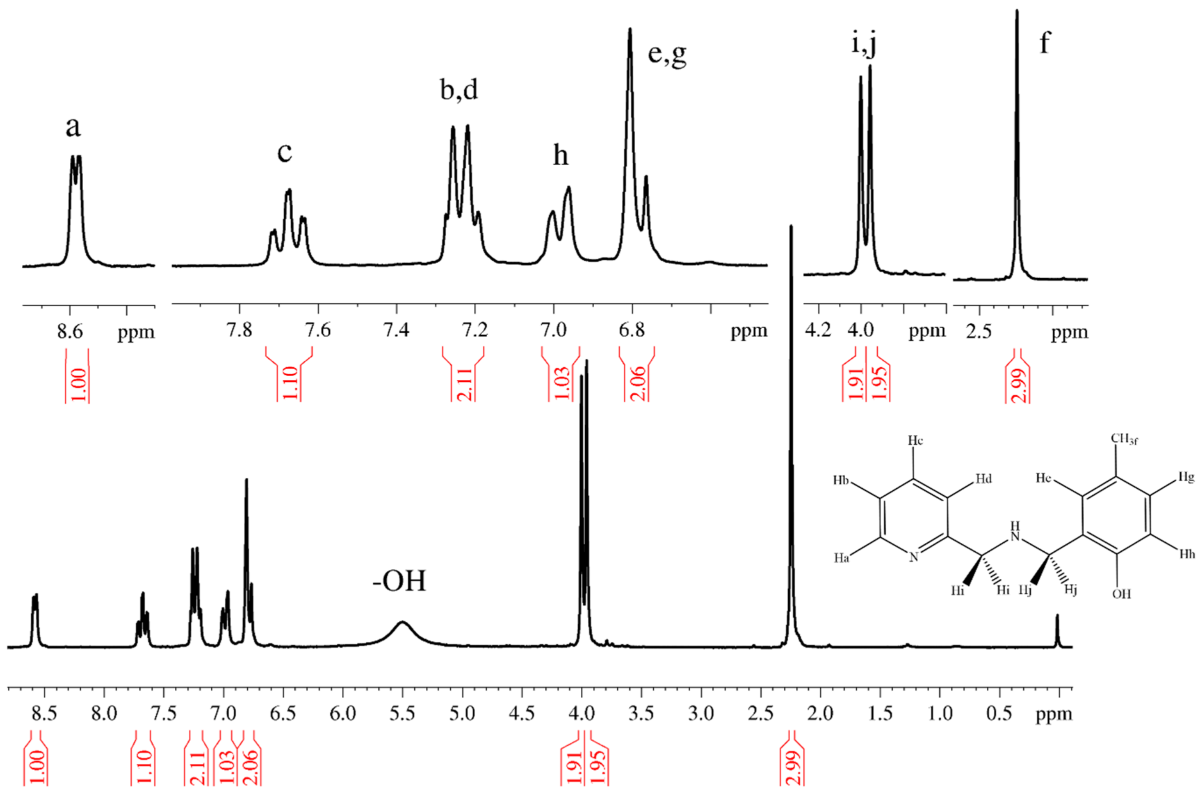

Figure S5. ^{1}H NMR (200 MHz, TMS= 0.00 ppm) spectrum of **Hbpa-Me** in $CDCl_3$.

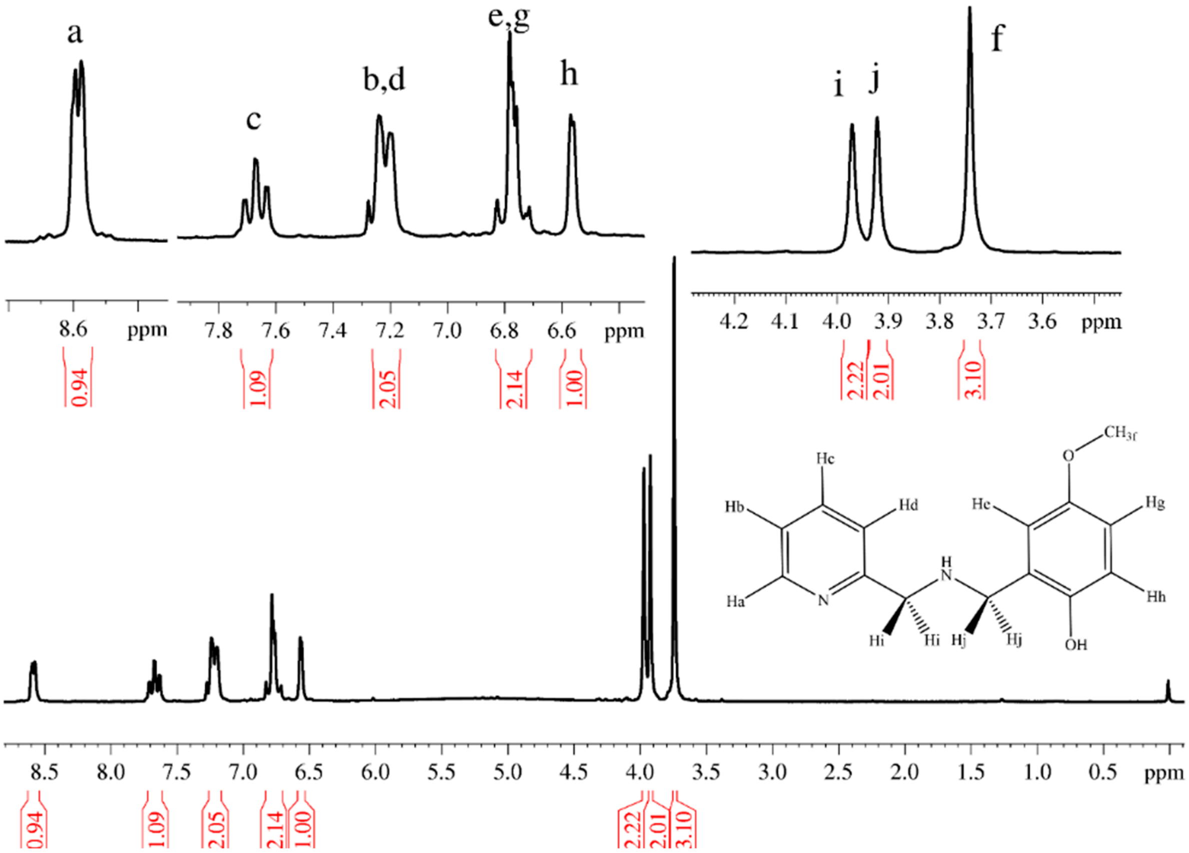

Figure S6. ^{1}H NMR (200 MHz, TMS= 0.00 ppm) spectrum of **Hbpa-OMe** in $CDCl_3$.

Table S1. Crystal data and structure refinement of compound **(1)**.

Empirical formula	$C_{16}H_{13}Br_2MnN_2O_4$	
Formula weight	512.04	
Temperature	150(2) K	
Wavelength	0.71073 Å	
Crystal system	Triclinic	
Space group	P -1	
Unit cell dimensions	a = 7.8714(7) Å	α= 97.177(2)°.
	b = 8.5609(7) Å	β= 105.699(2)°.
	c = 14.1438(12) Å	γ = 97.734(2)°.
Volume	896.10(13) $Å^3$	
Z	2	
Density (calculated)	1.898 Mg/m^3	
Absorption coefficient	5.218 mm^{-1}	
F(000)	500	
Crystal size	0.200 x 0.160 x 0.060 mm^3	
Theta range for data collection	1.517 to 30.577°.	
Index ranges	-11<=h<=11, -12<=k<=12, -20<=l<=18	
Reflections collected	15003	
Independent reflections	5509 [R(int) = 0.0258]	
Completeness to theta = 25.242°	100.0 %	
Absorption correction	Semi-empirical from equivalents	
Max. and min. transmission	0.7461 and 0.5574	
Refinement method	Full-matrix least-squares on F^2	
Data / restraints / parameters	5509 / 0 / 226	
Goodness-of-fit on F^2	1.021	
Final R indices [I>2sigma(I)]	R1 = 0.0279, wR2 = 0.0537	
R indices (all data)	R1 = 0.0482, wR2 = 0.0587	
Extinction coefficient	n/a	
Largest diff. peak and hole	0.605 and -0.577 e.$Å^{-3}$	

Table S2. Selected bond lengths (Å) and angles (°) for **(1)**.

**Bond Lengths (Å)**		**Bond Angles (°)**			
Mn(1)-C(3)	1.802(2)	C(3)-Mn(1)-C(2)	92.33(9)	N(12)-Mn(1)-N(1)	78.27(6)
Mn(1)-C(2)	1.811(2)	C(3)-Mn(1)-C(1)	90.69(9)	C(3)-Mn(1)-Br(1)	179.33(6)
Mn(1)-C(1)	1.815(2)	C(2)-Mn(1)-C(1)	87.96(9)	C(2)-Mn(1)-Br(1)	88.10(6)
Mn(1)-N(12)	2.0590(15)	C(3)-Mn(1)-N(12)	93.95(7)	C(1)-Mn(1)-Br(1)	89.84(6)
Mn(1)-N(1)	2.0856(16)	C(2)-Mn(1)-N(12)	171.78(7)	N(12)-Mn(1)-Br(1)	85.57(4)
Mn(1)-Br(1)	2.5254(4)	C(1)-Mn(1)-N(12)	97.25(8)	N(1)-Mn(1)-Br(1)	87.33(4)
C(1)-O(1)	1.142(3)	C(3)-Mn(1)-N(1)	92.11(8)	O(1)-C(1)-Mn(1)	176.58(17)
C(2)-O(2)	1.148(2)	C(2)-Mn(1)-N(1)	96.22(8)	O(2)-C(2)-Mn(1)	177.71(19)
C(3)-O(3)	1.134(2)	C(1)-Mn(1)-N(1)	174.86(7)	O(3)-C(3)-Mn(1)	179.56(18)

Symmetry transformations were used to generate equivalent atoms.

Table S3. Crystal data and structure refinement of compound **(2)**.

Empirical formula	$C_{16}H_{14}BrMnN_2O_4$	
Formula weight	433.14	
Temperature	150(2) K	
Wavelength	0.71073 Å	
Crystal system	Monoclinic	
Space group	P 21/n	
Unit cell dimensions	a = 13.5543(8) Å	α= 90°.
	b = 9.7453(6) Å	β= 93.5850(10)°.
	c = 26.0146(16) Å	γ = 90°.
Volume	3429.6(4) $Å^3$	
Z	8	
Density (calculated)	1.678 Mg/m^3	
Absorption coefficient	3.122 mm^{-1}	
F(000)	1728	
Crystal size	0.400 x 0.340 x 0.180 mm^3	
Theta range for data collection	1.569 to 30.145°.	
Index ranges	-18<=h<=19, -13<=k<=13, -36<=l<=36	
Reflections collected	38127	
Independent reflections	10119 [R(int) = 0.0186]	
Completeness to theta = 25.242°	100.0 %	
Absorption correction	Semi-empirical from equivalents	
Max. and min. transmission	0.7460 and 0.6301	
Refinement method	Full-matrix least-squares on F^2	
Data / restraints / parameters	10119 / 0 / 433	
Goodness-of-fit on F^2	1.085	
Final R indices [I>2sigma(I)]	R1 = 0.0272, wR2 = 0.0595	
R indices (all data)	R1 = 0.0359, wR2 = 0.0624	
Extinction coefficient	n/a	
Largest diff. peak and hole	0.937 and -0.467 e.$Å^{-3}$	

Table S4. Selected bond lengths (Å) and angles (°) for **(2)**.

**Bond Lengths (Å)**		**Bond Angles (°)**			
Mn(1)-C(3)	1.7948(19)	C(3)-Mn(1)-C(2)	90.62(8)	C(6)-Mn(2)-C(5)	88.82(8)
Mn(1)-C(2)	1.8119(19)	C(3)-Mn(1)-C(1)	90.85(9)	C(6)-Mn(2)-C(4)	90.47(8)
Mn(1)-C(1)	1.816(2)	C(2)-Mn(1)-C(1)	88.68(9)	C(5)-Mn(2)-C(4)	87.99(8)
Mn(1)-N(12)	2.0525(14)	C(3)-Mn(1)-N(12)	95.08(7)	C(6)-Mn(2)-N(32)	92.72(7)
Mn(1)-N(1)	2.0886(15)	C(2)-Mn(1)-N(12)	173.10(7)	C(5)-Mn(2)-N(32)	175.39(7)
Mn(1)-Br(1)	2.5273(3)	C(1)-Mn(1)-N(12)	95.11(7)	C(4)-Mn(2)-N(32)	96.34(7)
C(1)-O(1)	1.144(2)	C(3)-Mn(1)-N(1)	91.55(7)	C(6)-Mn(2)-N(2)	96.24(7)
C(2)-O(2)	1.145(2)	C(2)-Mn(1)-N(1)	96.62(7)	C(5)-Mn(2)-N(2)	96.94(7)
C(3)-O(3)	1.148(2)	C(1)-Mn(1)-N(1)	174.15(7)	C(4)-Mn(2)-N(2)	171.73(7)
Mn(2)-C(6)	1.7966(18)	N(12)-Mn(1)-N(1)	79.37(5)	N(32)-Mn(2)-N(2)	78.57(6)
Mn(2)-C(5)	1.8054(19)	C(3)-Mn(1)-Br(1)	176.63(6)	C(6)-Mn(2)-Br(2)	177.50(6)
Mn(2)-C(4)	1.8136(18)	C(2)-Mn(1)-Br(1)	88.71(6)	C(5)-Mn(2)-Br(2)	88.97(6)
Mn(2)-N(32)	2.0556(15)	C(1)-Mn(1)-Br(1)	92.43(7)	C(4)-Mn(2)-Br(2)	88.30(6)
Mn(2)-N(2)	2.0884(14)	N(12)-Mn(1)-Br(1)	85.38(4)	N(32)-Mn(2)-Br(2)	89.57(4)
Mn(2)-Br(2)	2.5517(3)	N(1)-Mn(1)-Br(1)	85.25(4)	N(2)-Mn(2)-Br(2)	85.17(4)
C(4)-O(4)	1.144(2)	O(1)-C(1)-Mn(1)	178.2(2)	O(4)-C(4)-Mn(2)	178.23(17)
C(5)-O(5)	1.142(2)	O(2)-C(2)-Mn(1)	178.26(19)	O(5)-C(5)-Mn(2)	177.18(15)
C(6)-O(6)	1.146(2)	O(3)-C(3)-Mn(1)	178.40(17)	O(6)-C(6)-Mn(2)	176.06(18)

Symmetry transformations were used to generate equivalent atoms.

Table S5. Crystal data and structure refinement of compound **(3)**.

Empirical formula	$C_{17}H_{16}BrMnN_2O_4$	
Formula weight	447.17	
Temperature	150(2) K	
Wavelength	0.71073 Å	
Crystal system	Triclinic	
Space group	P -1	
Unit cell dimensions	a = 7.7748(10) Å	α= 77.426(2)°.
	b = 8.4882(11) Å	β= 75.239(2)°.
	c = 14.1772(18) Å	γ = 84.486(2)°.
Volume	882.2(2) $Å^3$	
Z	2	
Density (calculated)	1.683 Mg/m^3	
Absorption coefficient	3.037 mm^{-1}	
F(000)	448	
Crystal size	0.240 x 0.180 x 0.120 mm^3	
Theta range for data collection	2.461 to 33.229°.	
Index ranges	-11<=h<=11, -13<=k<=13, -21<=l<=21	
Reflections collected	20422	
Independent reflections	6734 [R(int) = 0.0205]	
Completeness to theta = 25.242°	100.0 %	
Absorption correction	Semi-empirical from equivalents	
Max. and min. transmission	0.7465 and 0.6385	
Refinement method	Full-matrix least-squares on F^2	
Data / restraints / parameters	6734 / 0 / 227	
Goodness-of-fit on F^2	1.054	
Final R indices [I>2sigma(I)]	R1 = 0.0299, wR2 = 0.0685	
R indices (all data)	R1 = 0.0442, wR2 = 0.0735	
Extinction coefficient	n/a	
Largest diff. peak and hole	0.621 and -0.861 e.$Å^{-3}$	

Table S6. Selected bond lengths (Å) and angles (°) for **(3)**.

**Bond Lengths (Å)**		**Bond Angles (°)**			
Mn(1)-C(3)	1.7973(17)	C(3)-Mn(1)-C(2)	92.06(8)	N(12)-Mn(1)-N(1)	78.22(5)
Mn(1)-C(2)	1.8154(17)	C(3)-Mn(1)-C(1)	89.92(7)	C(3)-Mn(1)-Br(1)	179.34(5)
Mn(1)-C(1)	1.8214(17)	C(2)-Mn(1)-C(1)	87.42(7)	C(2)-Mn(1)-Br(1)	88.33(5)
Mn(1)-N(12)	2.0610(14)	C(3)-Mn(1)-N(12)	94.14(7)	C(1)-Mn(1)-Br(1)	90.63(5)
Mn(1)-N(1)	2.0860(14)	C(2)-Mn(1)-N(12)	171.94(6)	N(12)-Mn(1)-Br(1)	85.43(4)
Mn(1)-Br(1)	2.5258(3)	C(1)-Mn(1)-N(12)	97.73(7)	N(1)-Mn(1)-Br(1)	86.93(4)
C(1)-O(1)	1.142(2)	C(3)-Mn(1)-N(1)	92.49(7)	O(1)-C(1)-Mn(1)	176.09(15)
C(2)-O(3)	1.145(2)	C(2)-Mn(1)-N(1)	96.38(6)	O(3)-C(2)-Mn(1)	177.25(16)
O(2)-C(3)	1.141(2)	C(1)-Mn(1)-N(1)	175.42(6)	O(2)-C(3)-Mn(1)	179.64(16)

Symmetry transformations were used to generate equivalent atoms.

Table S7. Crystal data and structure refinement of compound **(4)**.

Empirical formula	$C_{17}H_{16}BrMnN_2O_5$	
Formula weight	463.17	
Temperature	150(2) K	
Wavelength	0.71073 Å	
Crystal system	Triclinic	
Space group	P -1	
Unit cell dimensions	a = 7.5412(9) Å	α= 79.549(2)°.
	b = 13.8743(16) Å	β= 87.930(2)°.
	c = 18.566(2) Å	γ = 77.108(2)°.
Volume	1862.1(4) $Å^3$	
Z	4	
Density (calculated)	1.652 Mg/m^3	
Absorption coefficient	2.884 mm^{-1}	
F(000)	928	
Crystal size	0.340 x 0.180 x 0.120 mm^3	
Theta range for data collection	1.726 to 32.822°.	
Index ranges	-11<=h<=11, -21<=k<=20, -28<=l<=27	
Reflections collected	40852	
Independent reflections	13728 [R(int) = 0.0470]	
Completeness to theta = 25.242°	100.0 %	
Absorption correction	Semi-empirical from equivalents	
Max. and min. transmission	0.7464 and 0.5408	
Refinement method	Full-matrix least-squares on F^2	
Data / restraints / parameters	13728 / 0 / 471	
Goodness-of-fit on F^2	1.069	
Final R indices [I>2sigma(I)]	R1 = 0.0491, wR2 = 0.1038	
R indices (all data)	R1 = 0.0862, wR2 = 0.1187	
Extinction coefficient	n/a	
Largest diff. peak and hole	1.543 and -1.166 e.$Å^{-3}$	

Table S8. Selected bond lengths (Å) and angles (°) for **(4)**.

**Bond Lengths (Å)**		**Bond Angles (°)**			
Mn(1)-C(3)	1.798(3)	C(3)-Mn(1)-C(1)	88.51(11)	C(6)-Mn(2)-C(4)	88.81(14)
Mn(1)-C(1)	1.814(3)	C(3)-Mn(1)-C(2)	93.08(12)	C(6)-Mn(2)-C(5)	86.60(14)
Mn(1)-C(2)	1.816(3)	C(1)-Mn(1)-C(2)	87.66(11)	C(4)-Mn(2)-C(5)	87.51(16)
Mn(1)-N(12)	2.052(2)	C(3)-Mn(1)-N(12)	92.39(10)	C(6)-Mn(2)-N(32)	93.25(11)
Mn(1)-N(1)	2.089(2)	C(1)-Mn(1)-N(12)	97.44(10)	C(4)-Mn(2)-N(32)	96.60(14)
Mn(1)-Br(1)	2.5320(5)	C(2)-Mn(1)-N(12)	172.61(10)	C(5)-Mn(2)-N(32)	175.88(12)
C(1)-O(1)	1.142(3)	C(3)-Mn(1)-N(1)	93.75(10)	C(6)-Mn(2)-N(2)	94.08(11)
C(2)-O(2)	1.145(3)	C(1)-Mn(1)-N(1)	175.99(10)	C(4)-Mn(2)-N(2)	174.43(13)
C(3)-O(3)	1.133(3)	C(2)-Mn(1)-N(1)	95.52(10)	C(5)-Mn(2)-N(2)	97.40(12)
Mn(2)-C(6)	1.775(3)	N(12)-Mn(1)-N(1)	79.17(8)	N(32)-Mn(2)-N(2)	78.50(9)
Mn(2)-C(4)	1.805(3)	C(3)-Mn(1)-Br(1)	177.70(8)	C(6)-Mn(2)-Br(2)	179.00(10)
Mn(2)-C(5)	1.815(4)	C(1)-Mn(1)-Br(1)	89.92(8)	C(4)-Mn(2)-Br(2)	92.13(11)
Mn(2)-N(32)	2.052(2)	C(2)-Mn(1)-Br(1)	88.53(8)	C(5)-Mn(2)-Br(2)	93.78(10)
Mn(2)-N(2)	2.086(2)	N(12)-Mn(1)-Br(1)	86.14(6)	N(32)-Mn(2)-Br(2)	86.31(6)
Mn(2)-Br(2)	2.5349(5)	N(1)-Mn(1)-Br(1)	87.72(6)	N(2)-Mn(2)-Br(2)	84.95(6)
C(4)-O(4)	1.142(4)	O(1)-C(1)-Mn(1)	176.5(2)	O(4)-C(4)-Mn(2)	176.5(3)
C(5)-O(5)	1.149(4)	O(2)-C(2)-Mn(1)	177.3(2)	O(5)-C(5)-Mn(2)	174.1(3)
C(6)-O(6)	1.150(4)	O(3)-C(3)-Mn(1)	176.9(2)	O(6)-C(6)-Mn(2)	176.9(3)

Symmetry transformations were used to generate equivalent atoms.

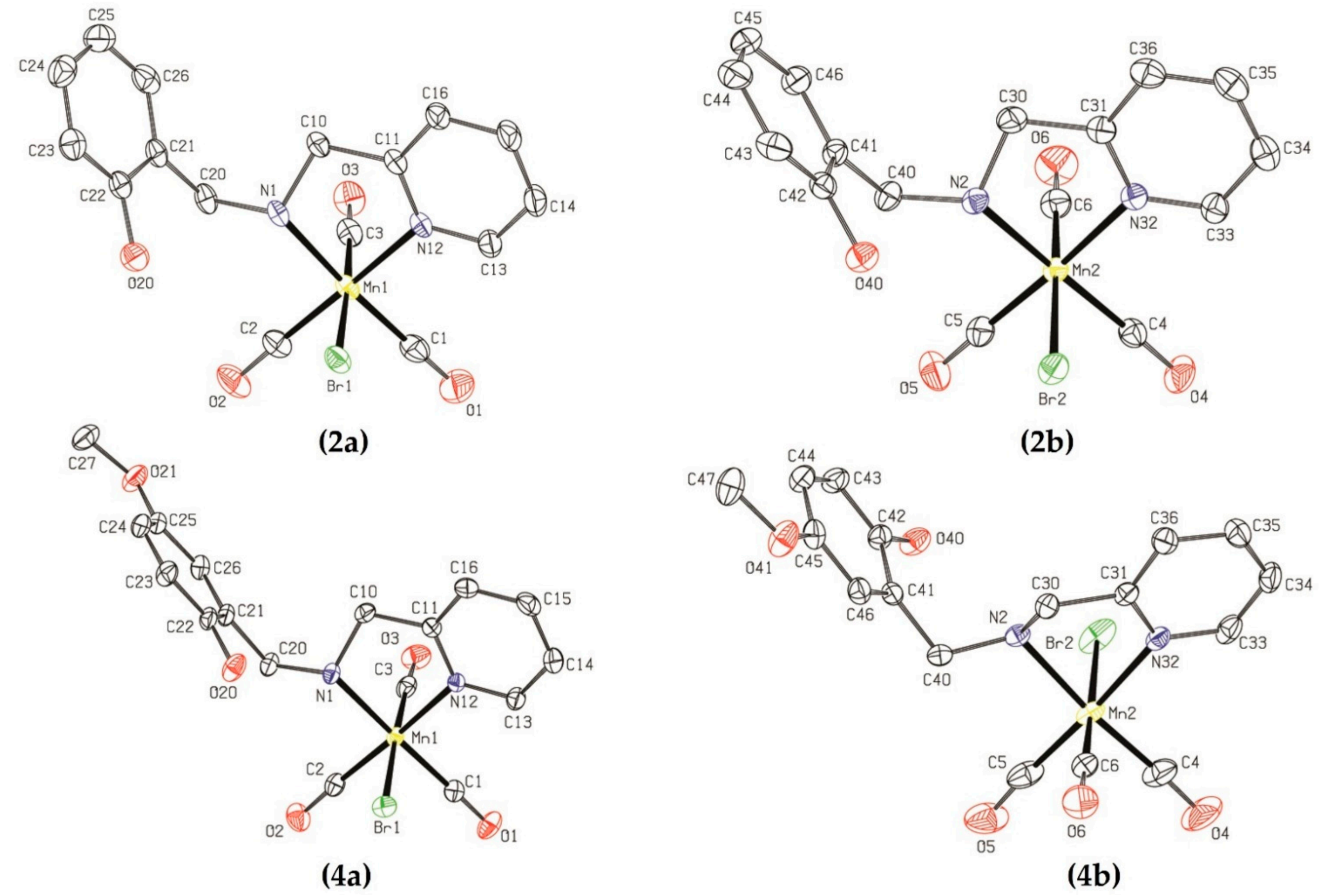

Figure S7. Isomers and conformers crystal structures obtained for compounds **(2a)** (upper left), **(2b)** (upper right), **(4a)** (lower left) and **(4b)** (lower right). Ellipsoids drawn at 40% probability level. Hydrogen atoms were omitted for clarity. Colors: Gray = carbon, red = oxygen, green = bromine, blue = nitrogen, yellow = manganese.

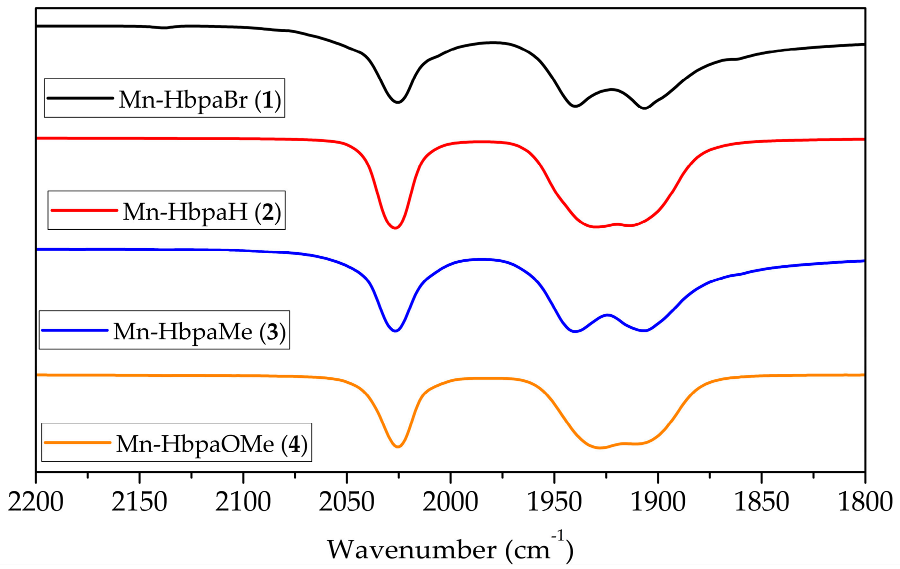

Figure S8. IR spectra (FTIR, KBr pellets) of compounds **(1)-(4)** in the range of 2200-1800 cm^{-1}, focusing on the carbonyl absorption region.

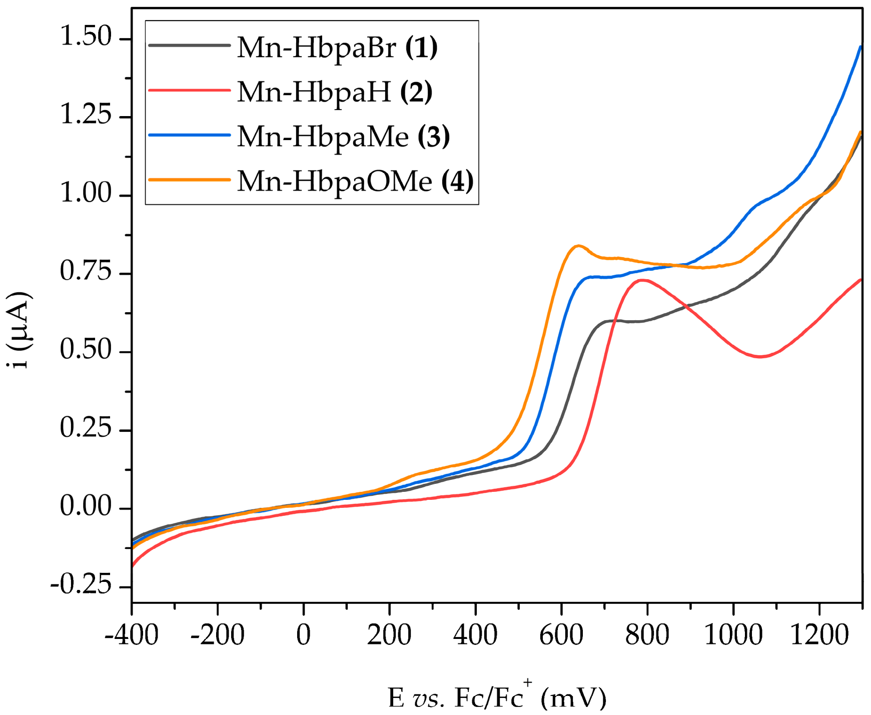

Figure S9. Square Wave voltammograms of compounds **(1)-(4)** in dichloromethane under argon atmosphere. Experimental conditions: 0.1 mol L^{-1} $TBAPF_6$ as the supporting electrolyte, Ag/AgCl as the reference electrode and platinum as the work and auxiliary electrodes. Potentials are referenced to the Fc/Fc^+ redox couple ($E_{1/2}$= 0.405 V *vs* Ag/AgCl in dichloromethane). Step: 4 mV, and square wave frequency: 15 Hz.

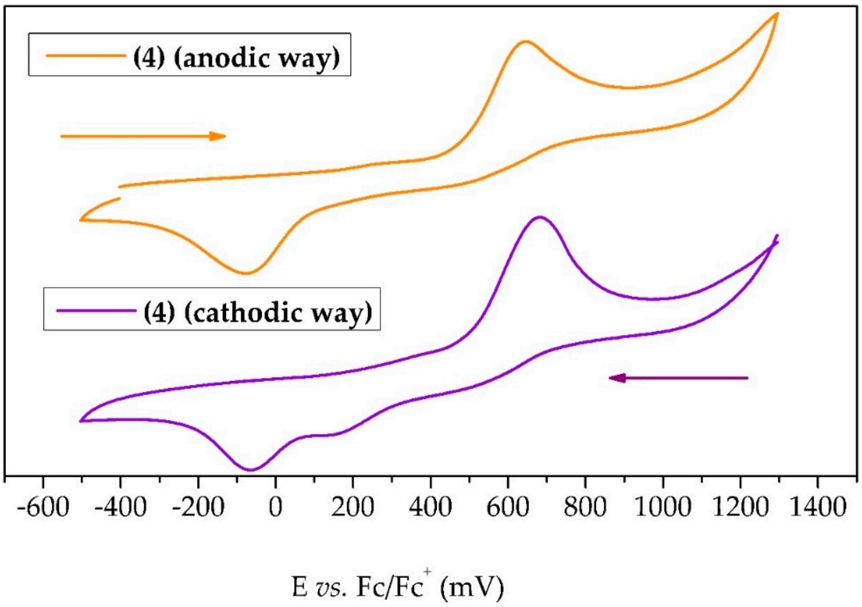

Figure S10. Cyclic voltammogram of compound **(4)** measured in both scan directions (125 mV/s) in dichloromethane under argon atmosphere. Experimental conditions: 0.1 mol L^{-1} $TBAPF_6$ as the supporting electrolyte, Ag/AgCl as the reference electrode and platinum as the work and auxiliary electrodes. Potentials are referenced to the Fc/Fc^+ redox couple ($E_{1/2}$= 0.405 V *vs* Ag/AgCl in dichloromethane).

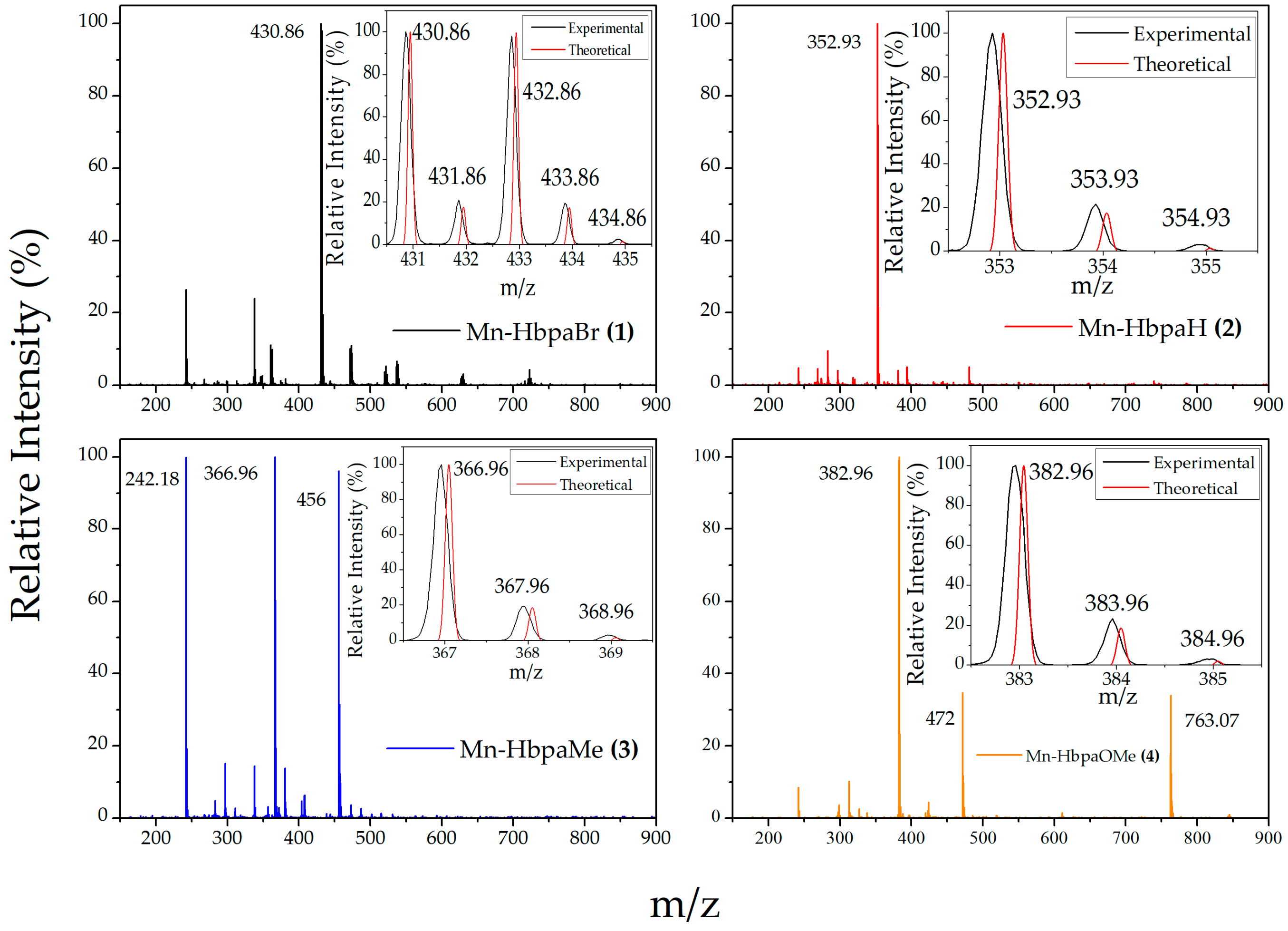

Figure S11. ESI-MS (+) spectra of compounds **(1)-(4)** in acetonitrile.

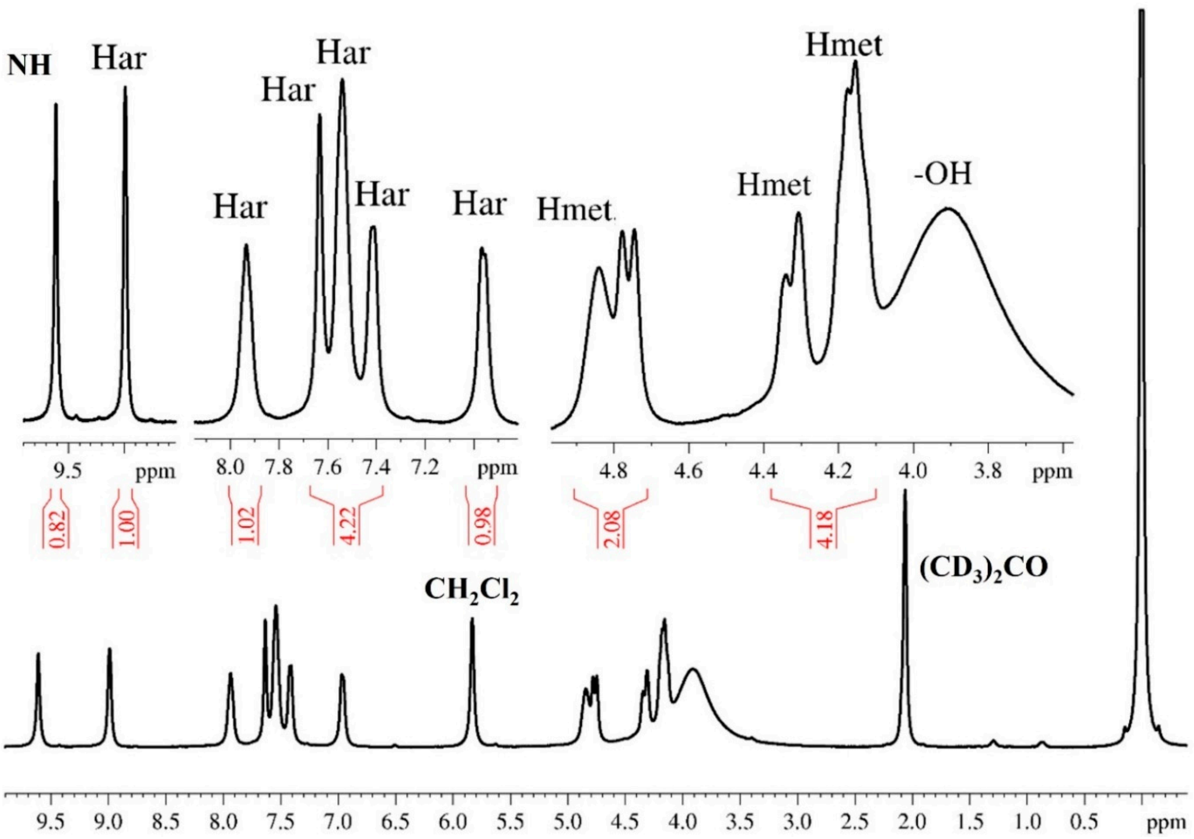

Figure S12. ^{1}H NMR (400 MHz, TMS= 0.00 ppm) of compound **(1)** in $(CD_3)_2CO$.

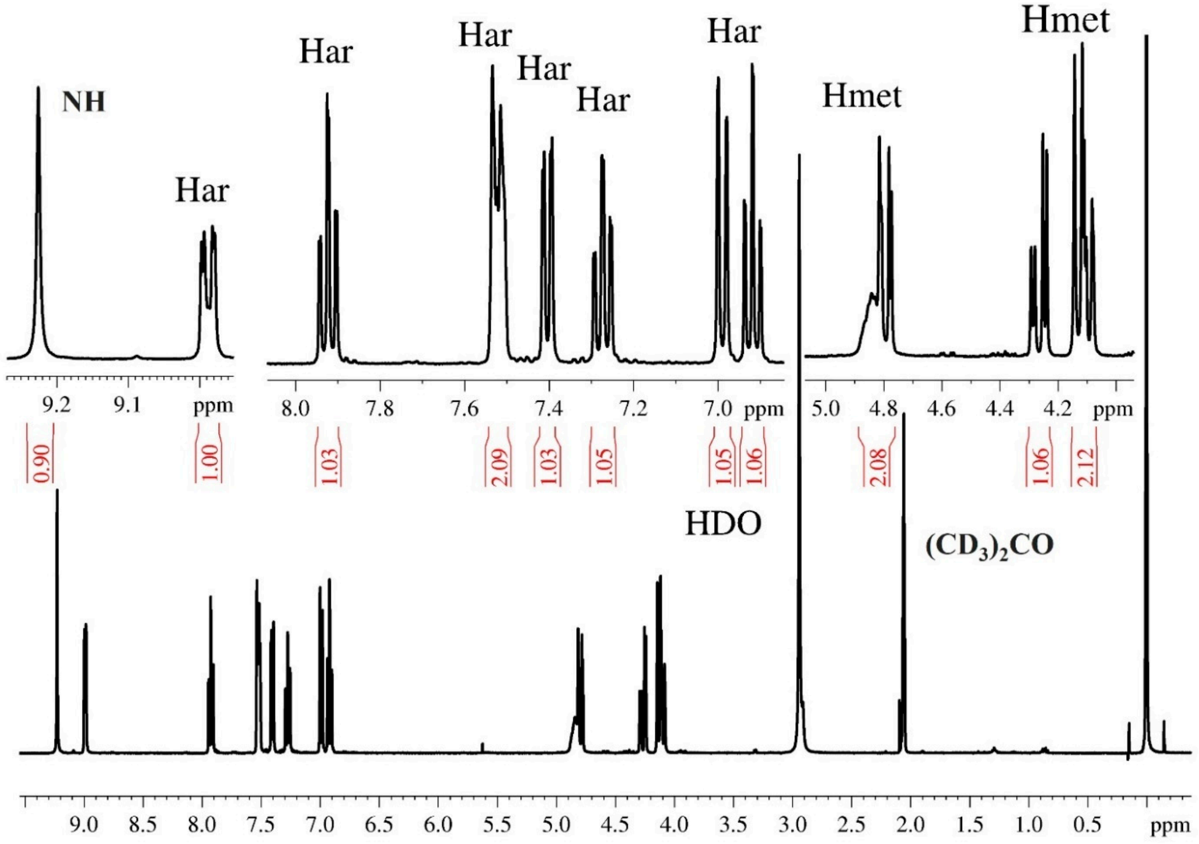

Figure S13. ^{1}H NMR (400 MHz, TMS= 0.00 ppm) of compound **(2)** in $(CD_3)_2CO$.

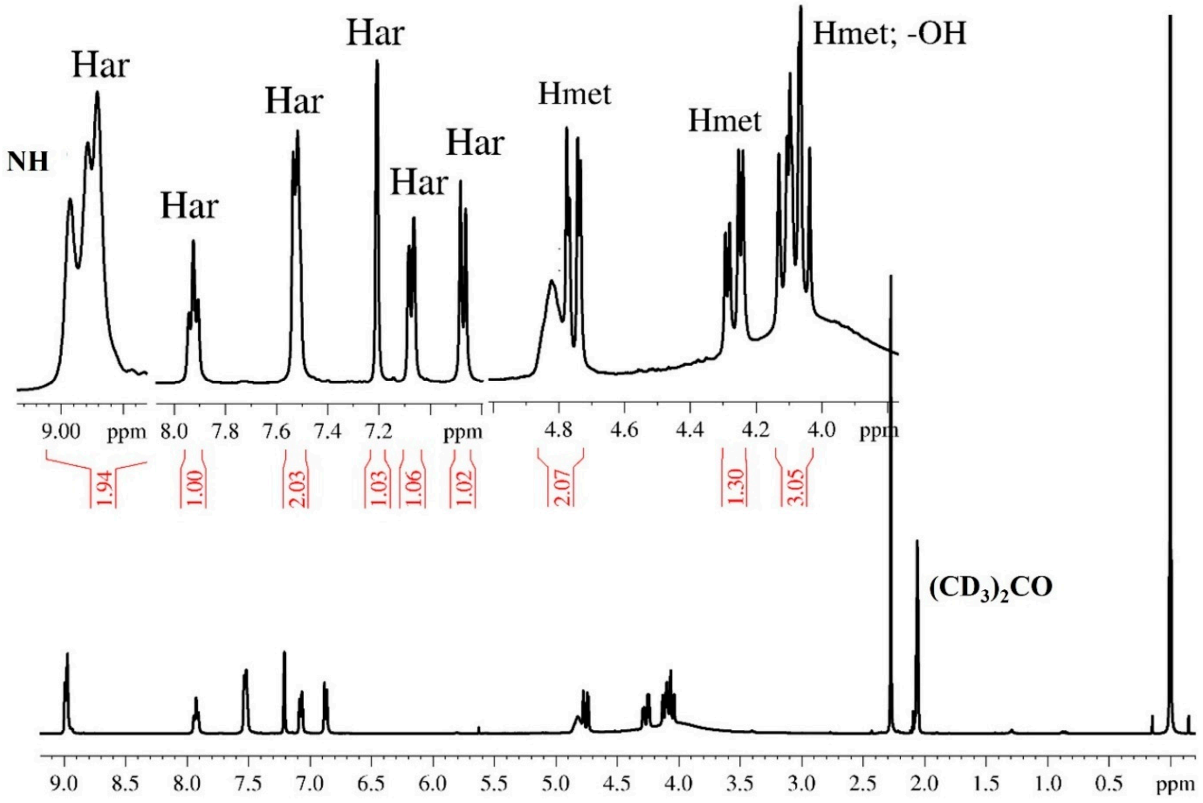

Figure S14. ^{1}H NMR (400 MHz, TMS= 0.00 ppm) of compound **(3)** in $(CD_3)_2CO$.

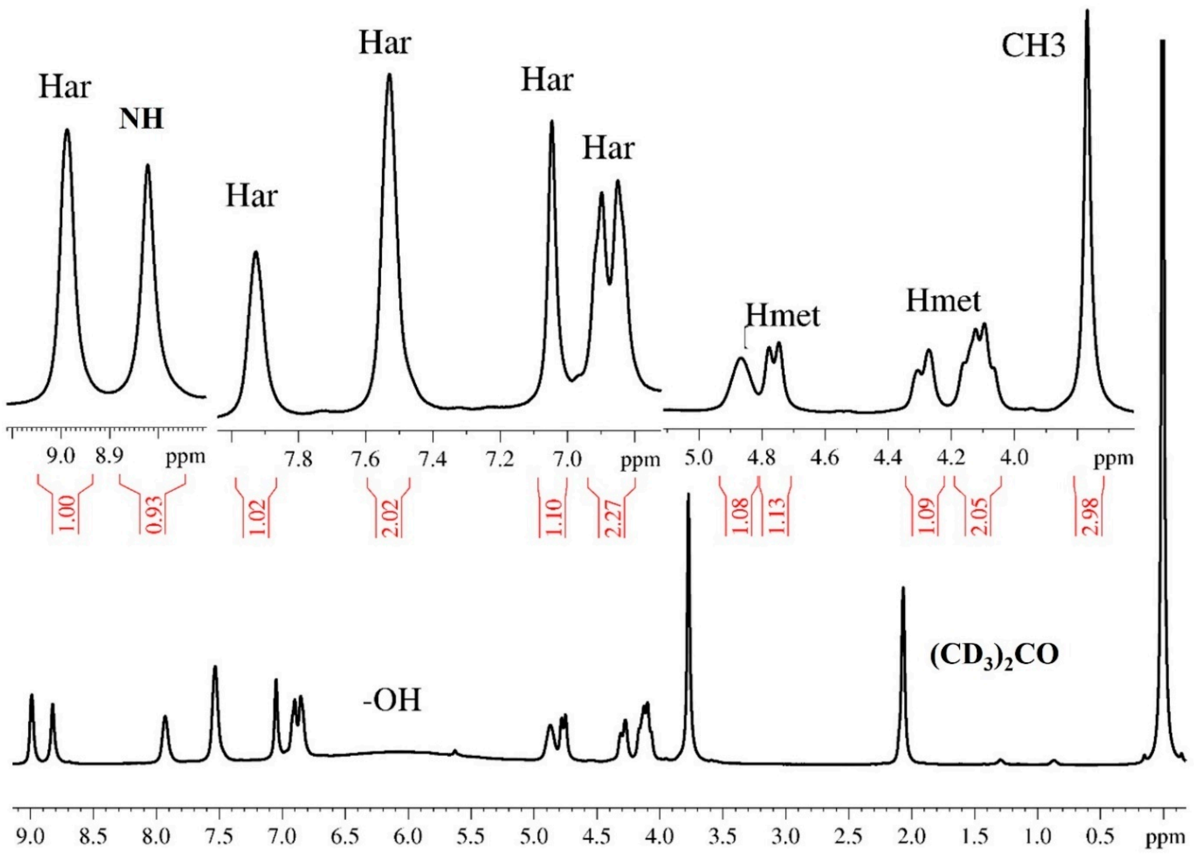

.

Figure S15. ^{1}H NMR (400 MHz, TMS= 0.00 ppm) of compound **(4)** in $(CD_3)_2CO$.

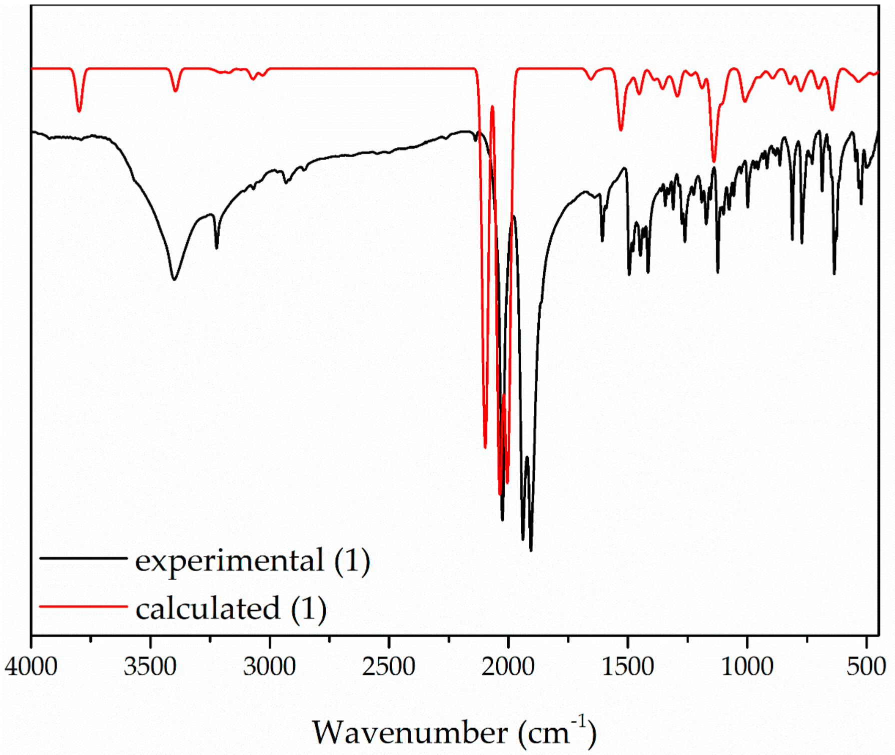

Figure S16. Comparison between experimental (black) and calculated (red) infrared spectra for compound **(1)**.

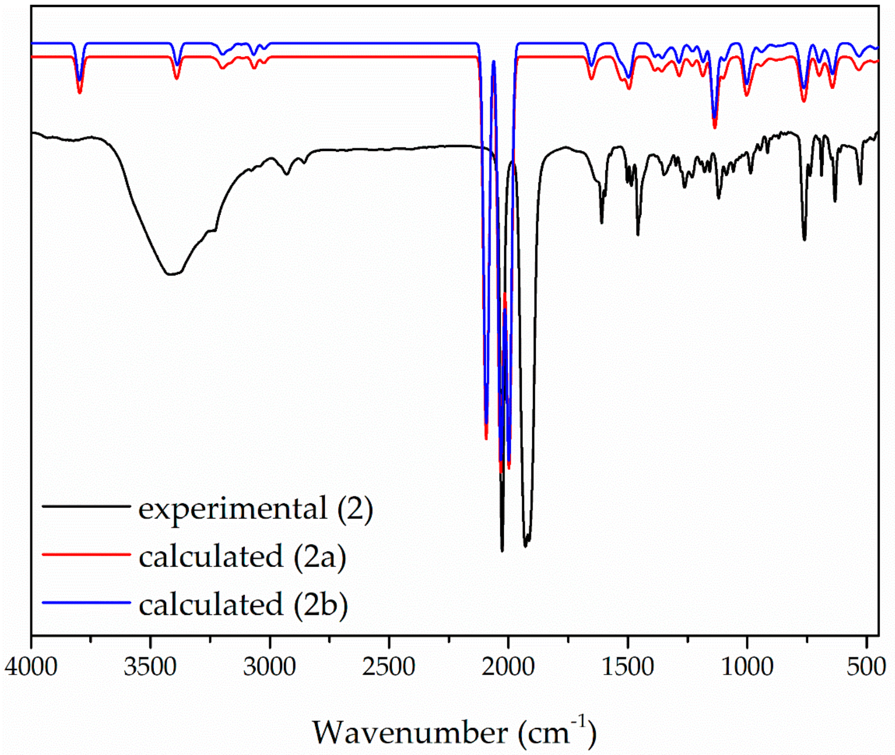

Figure S17. Comparison between experimental (black) and calculated (blue and red) infrared spectra for compounds **(2a)** and **(2b)**.

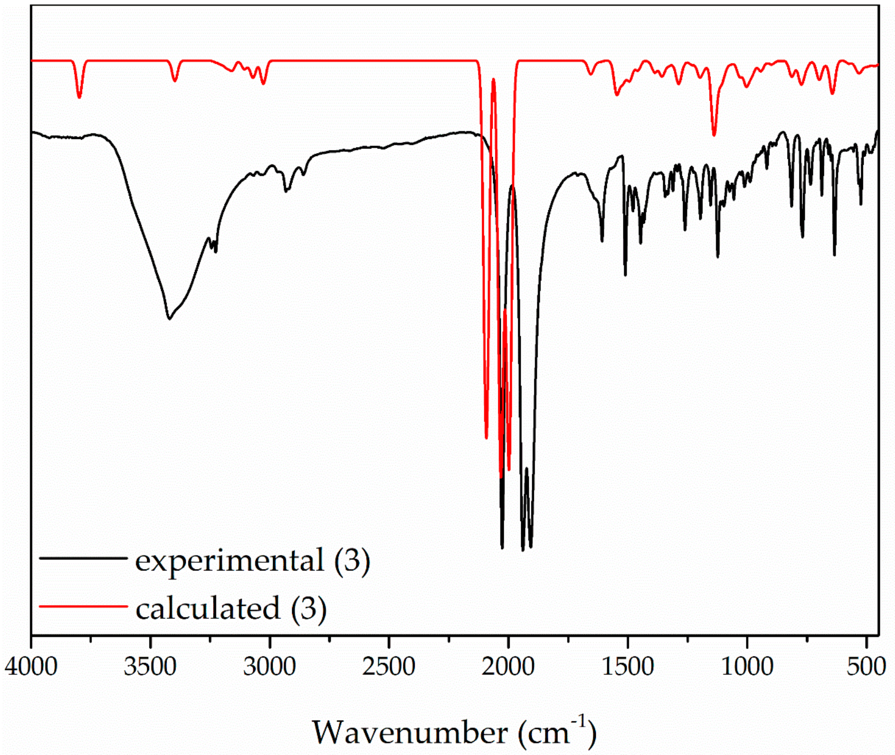

Figure S18. Comparison between experimental (black) and calculated (red) infrared spectra for compound **(3)**.

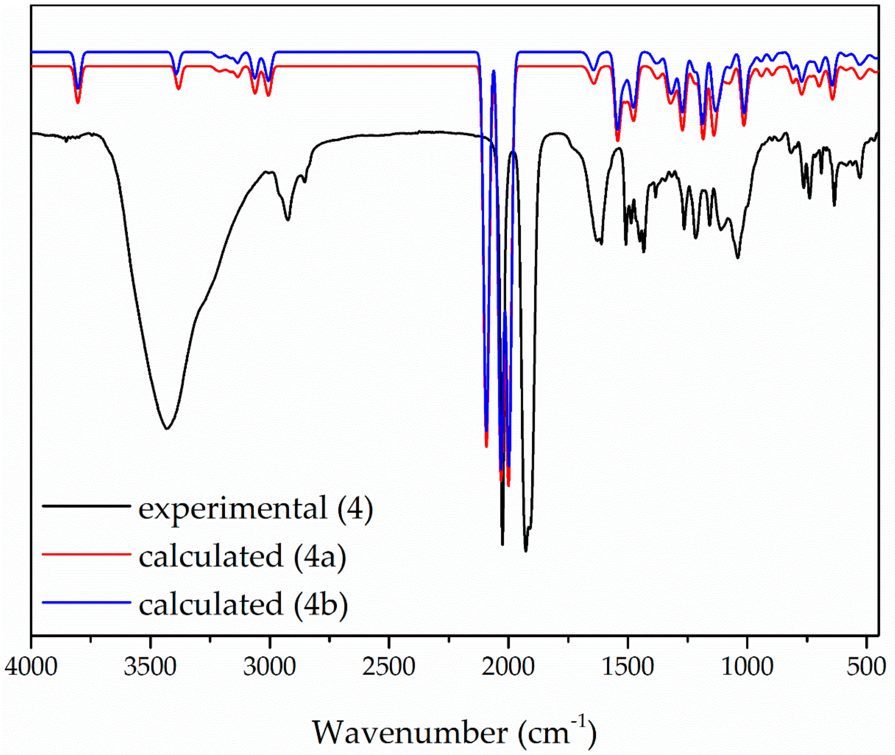

Figure S19. Comparison between experimental (black) and calculated (blue and red) infrared spectra for compounds **(4a)** and **(4b)**.

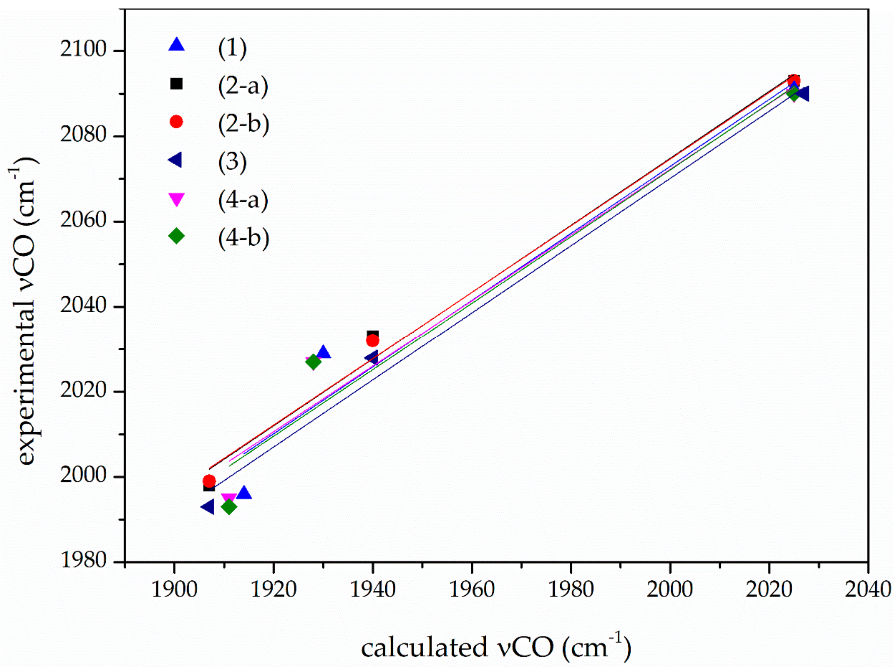

Figure S20. Comparison between experimental and calculated CO stretching values of compounds **(1)**-**(4)**.

Table S9. Data for the TD-DFT excitations using B3LYP/def2-TZVP for Br and Mn, and B3LYP/def2-TZVP(-f) for the other atoms.

			**(2a)**		
**State**	**Energy (nm)**		f_{osc}	**Transition**[1]	**attribution**
	**exp**	**calc**			
			**(1)**		
S_2		381	0.02577	H-1 → L+3 (30.5%)	Br-Mn-CO → Mn-CO
				H-1 → L (15.3%)	Br-Mn-CO → π* pyridyl
S_7		326	0.01974	H → L (56.0%)	Br-Mn-CO → π* pyridyl
S_{11}		294	0.01869	H-3 L → L (34.0%)	Mn-CO → π* pyridyl
S_{22}		261	0.04458	H-2 → L+1 (49.3%)	π phenol → π* phenol + Mn-CO
			**(2a)**		
S_2		382	0.02699	H-1 → L+3 (21.5%)	Br-Mn-CO → Mn-CO
				H-1 → L+2 (16.1%)	Br-Mn-CO → N_{aliph}-Mn-CO + π* phenol
				H-1 → L (15.8)	Br-Mn-CO → π* pyridyl
S_7		326	0.02201	H → L (56.9%)	Br-Mn-CO → π* pyridyl
S_{11}		294	0.01900	H-2 → L (30.4%)	π phenol Mn-CO → π* pyridyl
S_{26}		250	0.06357	H-3 → L+2 (31.2%)	π phenol + Mn-CO → N_{aliph}-Mn-CO + π* phenol
				H-2 → L +2 (19.3%)	π phenol + Mn-CO → N_{aliph}-Mn-CO + π* phenol
			**(3)**		
S_2		381	0.02603	H-1 → L (16.0%)	Br-Mn-CO → π* pyridyl
				H-1 → L+2 (19.4%)	Br-Mn-CO → Mn-CO + π* phenol
				H-1 → L+3 (19.6%)	Br-Mn-CO → Mn-CO
S_7		327	0.01918	H → L (53.0%)	Br-Mn-CO → π* pyridyl
S_{11}		294	0.01621	H-3 → L (22.1%)	Br-Mn-CO → π* pyridyl
				H-1 → L+5 (15.6%)	Br-Mn-CO → Mn-CO + π* phenol
S_{22}		260	0.07463	H-2 → L+2 (80.5%)	π phenol → Mn-CO + π* phenol
			**(4a)**		
S_2		382	0.02526	H-2 → L+3 (22.7%)	Br-Mn-CO → Mn-CO
				H-2 → L+2 (16.6%)	Br-Mn-CO → Mn-CO + π* phenol
				H-2 → L (16.0%)	Br-Mn-CO → π* pyridyl
S_7		326	0.02048	H-1 → L (54.0%)	Br-Mn-CO → π* pyridyl
S_{12}		294	0.01467	H-3 → L (26.8%)	Mn-CO → π* pyridyl
				H-2 → L+5 (20.8%)	Br-Mn-CO → Mn-CO
S_{16}		280	0.07966	H → L+2 (87.7%)	π phenol → Mn-CO + π* phenol

[1] Transitions with high percentage contributions (>15%) are shown in parenthesis.

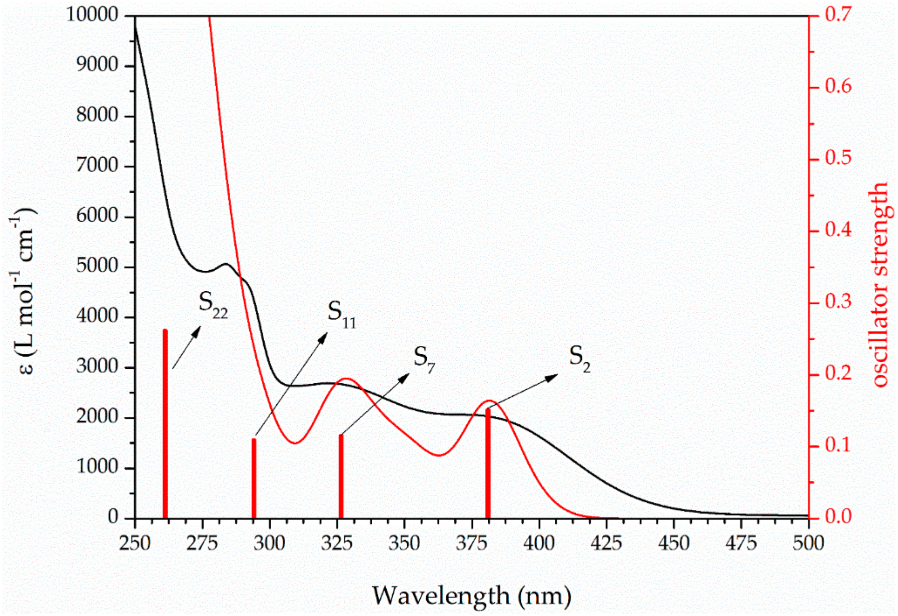

Figure S21. Comparison between experimental (black) and calculated (red) electronic spectra for compound **(1)**.

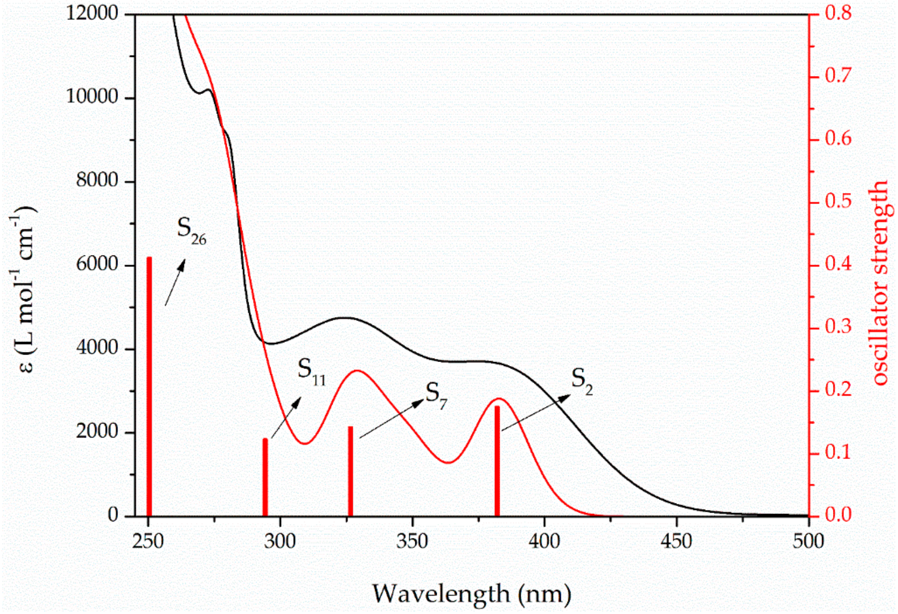

Figure S22. Comparison between experimental (black) and calculated (red) electronic spectra for compound **(2a)**.

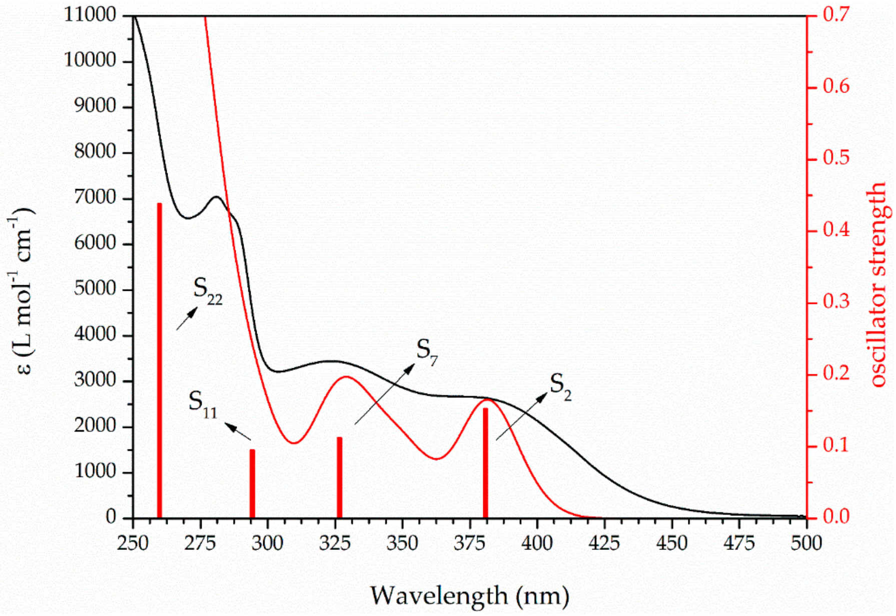

Figure S23. Comparison between experimental (black) and calculated (red) electronic spectra for compound **(3)**.

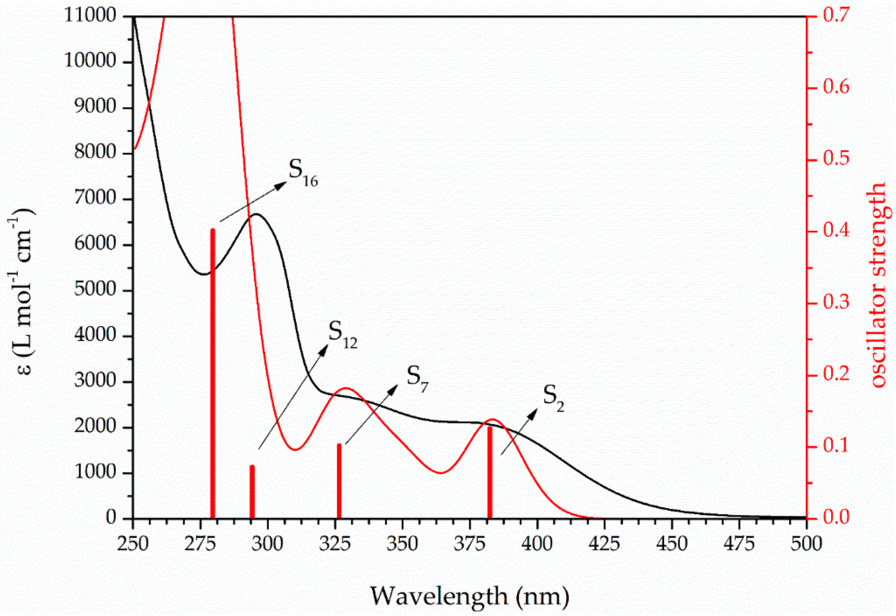

Figure S24. Comparison between experimental (black) and calculated (red) electronic spectra for compound **(4a)**.

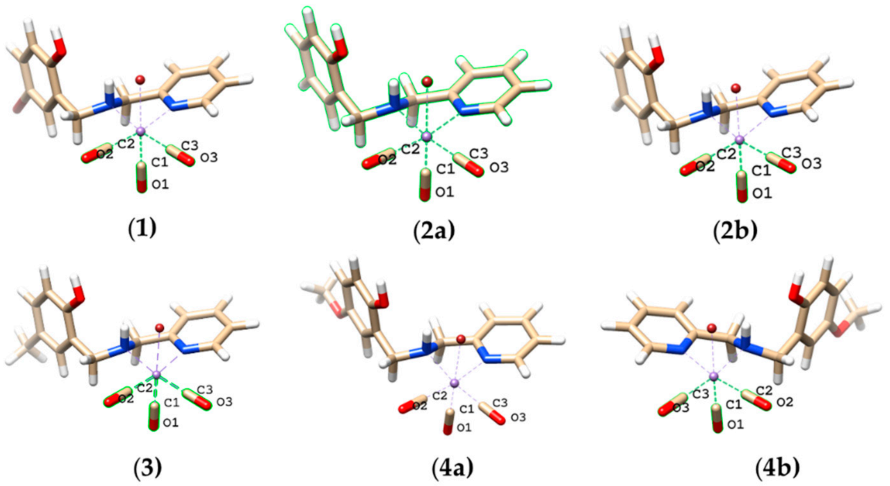

Figure S25. Fragments utilized for energy decomposition analysis.

Table S10. The most relevant density flow channel (Δρ) with their respective energies (ΔEoi) and charge transfer estimation (Δq) values for compounds synthetized in this study.

	**1-CO**		**2-CO**		**3-CO**	
Δρ	Δq	ΔE_{oi}	Δq	ΔE_{oi}	Δq	ΔE_{oi}
			**(1)**			
1	0.65	-40.42	0.61	-37.74	0.62	-38.31
2	0.56	-29.28	0.52	-24.62	0.52	-24.89
3	0.46	-23.70	0.48	-22.99	0.47	-23.20
4	0.10	-1.92	0.10	-2.04	0.10	-2.02
			**(2a)**			
1	0.66	-40.75	0.62	-38.16	0.62	-38.11
2	0.56	-29.41	0.52	-24.62	0.52	-24.94
3	0.46	-23.68	0.48	-23.07	0.47	-23.26
4	0.10	-1.90	0.10	-2.07	0.10	-2.05
			**(2b)**			
1	0.65	-40.25	0.61	-37.78	0.62	-38.05
2	0.56	-29.36	0.52	-24.53	0.52	-25.04
3	0.46	-23.72	0.48	-22.93	0.47	-23.33
4	0.10	-1.93	0.10	-2.04	0.10	-2.01
			**(3)**			
1	0.66	-40.64	0.61	-37.84	0.62	-38.18
2	0.56	-29.40	0.52	-24.66	0.52	-24.93
3	0.46	-23.70	0.48	-23.09	0.47	-23.20
4	0.10	-1.92	0.10	-2.04	0.10	-2.06
			**(4a)**			
1	0.65	-40.22	0.61	-37.74	0.62	-37.96
2	0.56	-29.31	0.52	-24.35	0.52	-25.00
3	0.46	-23.75	0.48	-22.82	0.47	-23.30
4	0.10	-1.91	0.10	-2.06	0.10	-2.00
			**(4b)**			
1	0.66	-40.94	0.62	-37.95	0.62	-38.05
2	0.56	-29.42	0.52	-24.46	0.52	-25.02
3	0.46	-23.67	0.48	-22.94	0.47	-23.31
4	0.10	-1.91	0.10	-2.11	0.10	-2.04

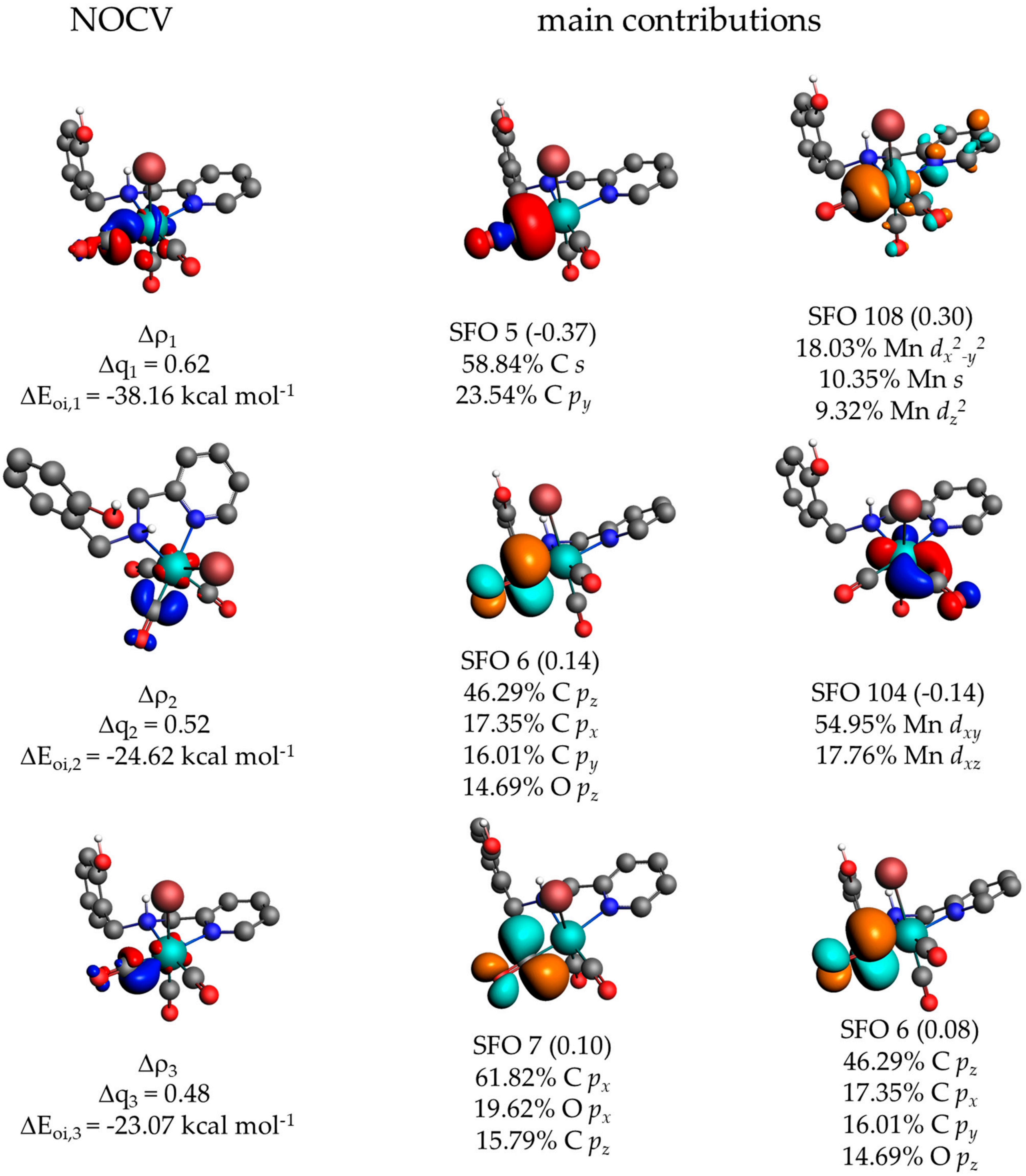

Figure S26. Plot of the most relevant density flow channel (Δρ) with contributing more than 2.0 kcal.mol^{-1} with their respective energies (ΔE_{oi}) and charge transfer estimation (Δq) values for fragment 2-CO (*trans* to pyridyl) of compound **(2a)** and the associated orbitals of the fragments with the greatest contribution for each density flow channel. The direction of the charge flow is red to blue. Hydrogens attached to carbon are omitted for clarity. The isovalue for NOCV is 0.005 and for the SFO is 0.05.

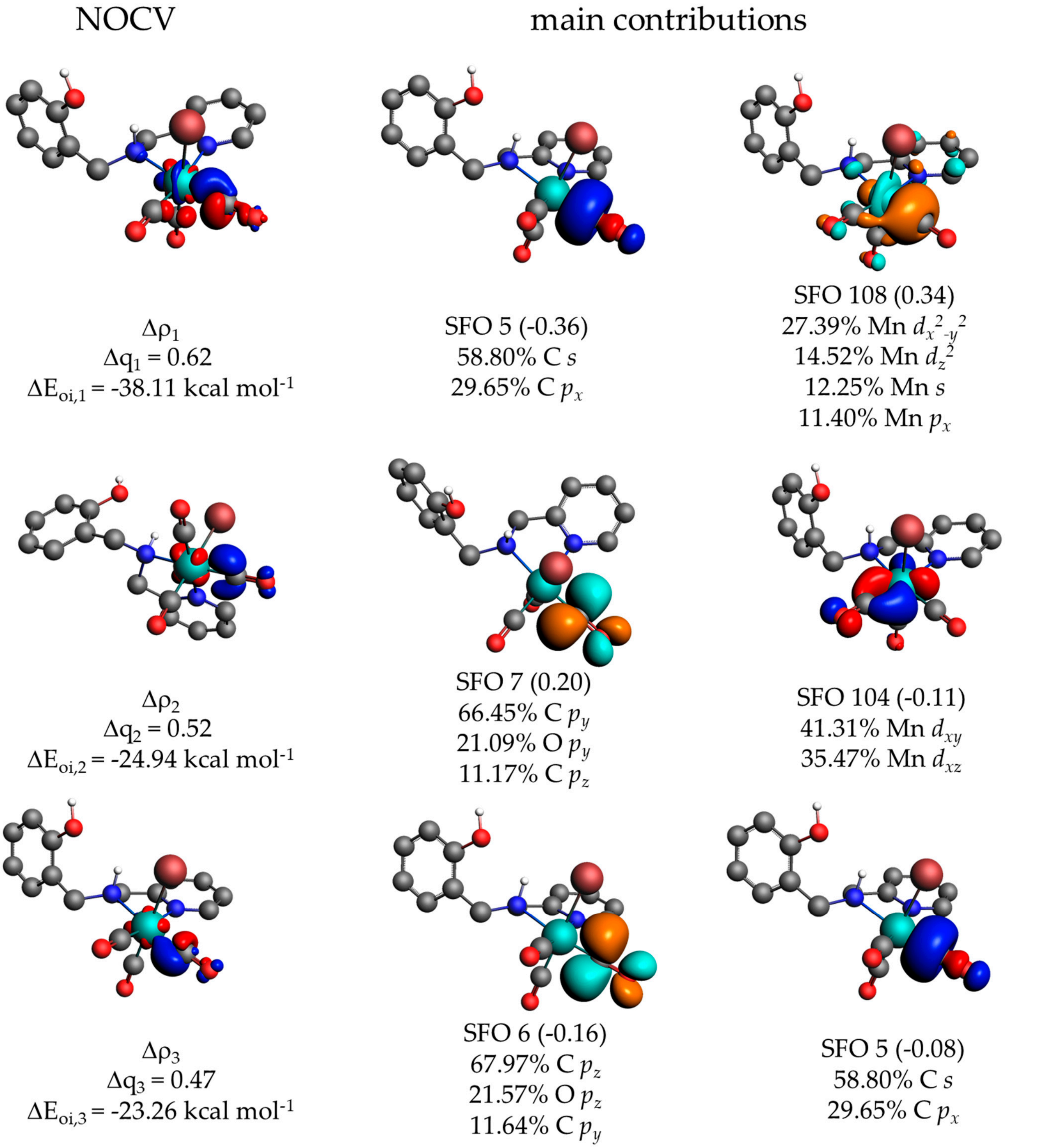

Figure S27. Plot of the most relevant density flow channel (Δρ) with contributing more than 2.0 kcal.mol^{-1} with their respective energies (ΔE_{oi}) and charge transfer estimation (Δq) values for fragment 3-CO (*trans* to amine) of compound **(2a)** and the associated orbitals of the fragments with the greatest contribution for each density flow channel. The direction of the charge flow is red to blue. Hydrogens attached to carbon are omitted for clarity. The isovalue for NOCV is 0.005 and for the SFO is 0.05.

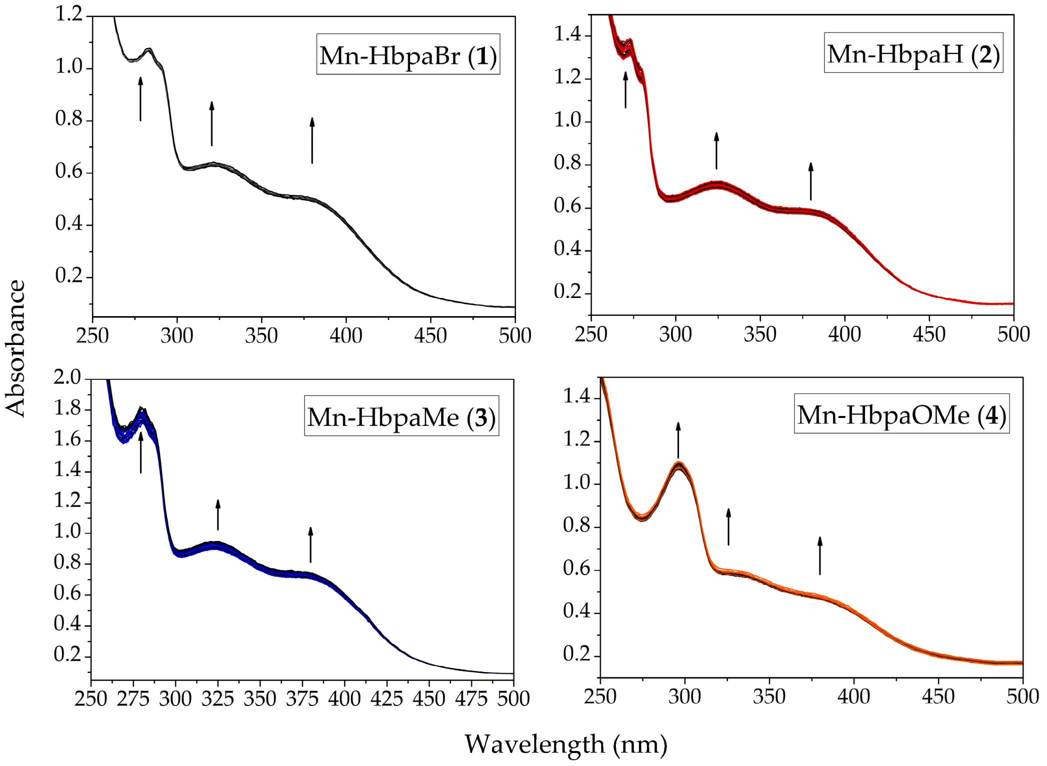

Figure S28. 24h timelapse in UV-Vis region of compounds **(1)-(4)** in dichloromethane. Spectra were collected in a 1h time interval. Sealed quartz cuvettes were kept in the dark during the whole experiment. [**(1)**] = 2.13×10^{-4}, [**(2)**] = 2.58×10^{-4}, [**(3)**] = **2.**99×10^{-4} and [**(4)**] = 1.89×10^{-4} mol L^{-1}.

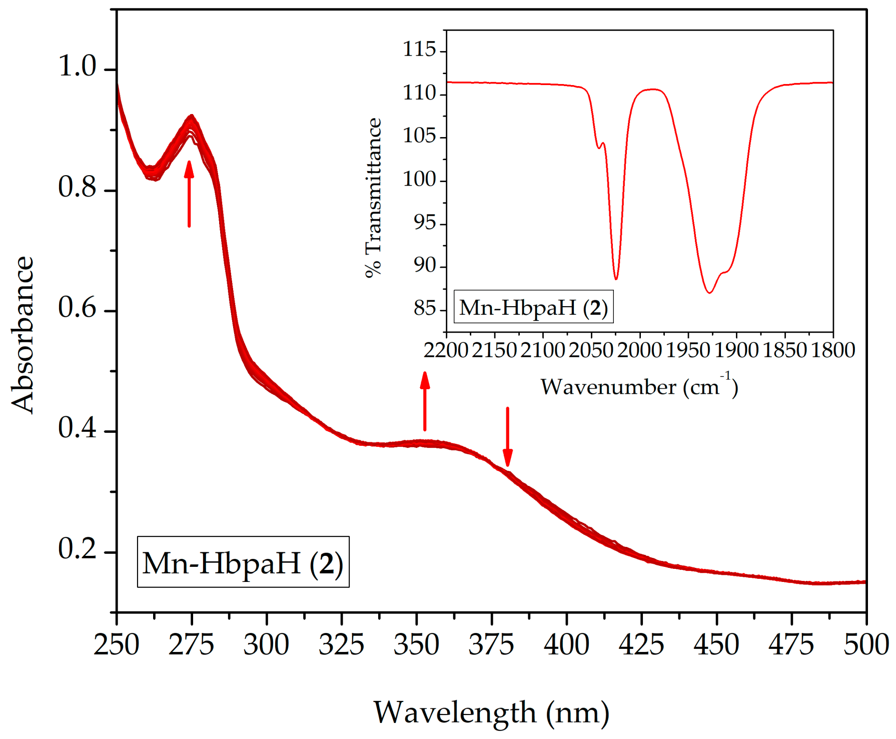

Figure S29. Spectral changes of **(2)** followed by UV-Vis spectroscopy. Spectra were collected in a 1h time interval in CH_3CN. Sealed quartz curvettes were kept in the dark during the whole experiment. [**(2)**]= 1.43×10^{-4} mol L^{-1}. Inset: IR spectra of **(2)** (KBr pellets) performed with performed with 100 μL of stock solution (1.00×10^{-3} mol L^{-1}) after 24 h in the dark.

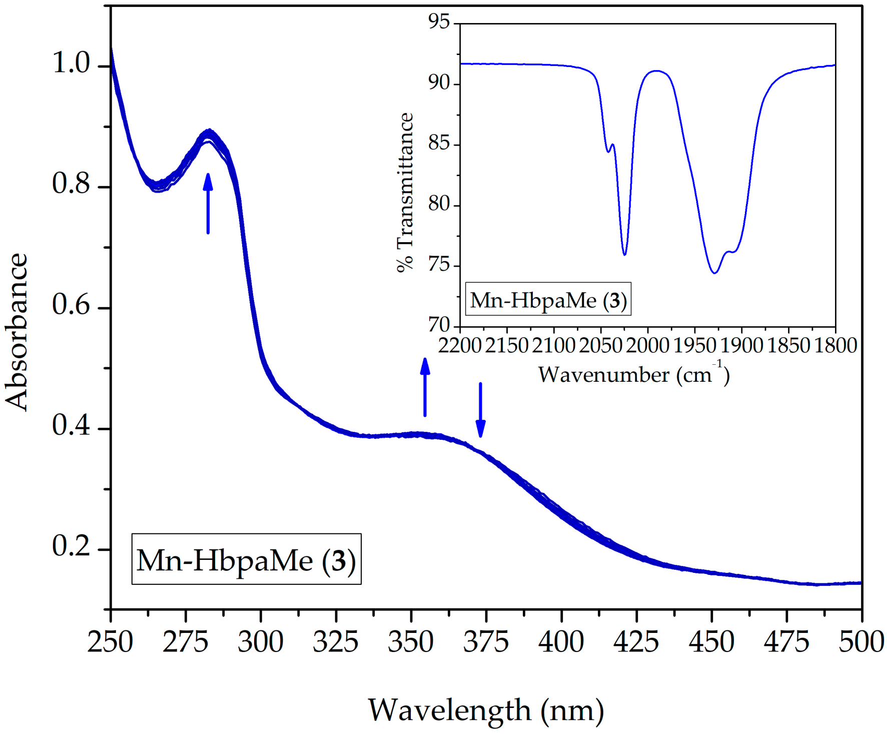

Figure S30. Spectral changes of **(3)** followed by UV-Vis spectroscopy. Spectra were collected in a 1h time interval in CH_3CN. Sealed quartz curvettes were kept in the dark during the whole experiment. [**(3)**]= 1.49×10^{-4} mol L^{-1}. Inset: IR spectra of **(3)** (KBr pellets) with performed with 100 μL of stock solution (1.00×10^{-3} mol L^{-1}) after 24 h in the dark.

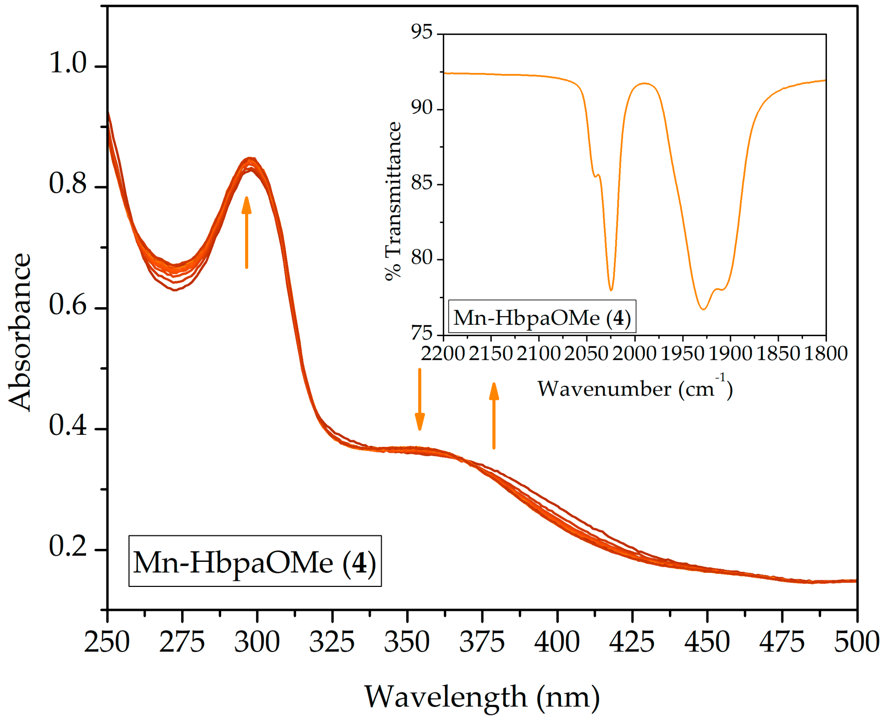

Figure S31. Spectral changes of **(3)** followed by UV-Vis spectroscopy. Spectra were collected in a 1h time interval in CH_3CN. Sealed quartz curvettes were kept in the dark during the whole experiment. [**(4)**]= 1.43×10^{-4} molL^{-1}. Inset: IR spectra of **(4)** (KBr pellets) with performed with 100 μL of stock solution (1.00×10^{-3} mol.L^{-1}) after 24 h in the dark.

Table S11. Molar conductivity variation of compounds **(1)-(4)** in acetonitrile and dichloromethane over 24h. Solutions of 1.00×10^{-3} mol L^{-1} of compounds were kept in the dark.

Compound	CH_2Cl_2 (μS cm^2 mol^{-1})		CH_3CN (μS cm^2 mol^{-1})	
	0h	24h	0h	24h
**(1)**	5.89	5.88	37.38	51.09
**(2)**	1.92	4.85	42.43	46.54
**(3)**	3.22	3.26	44.89	56.69
**(4)**	3.27	3.34	44.41	53.03

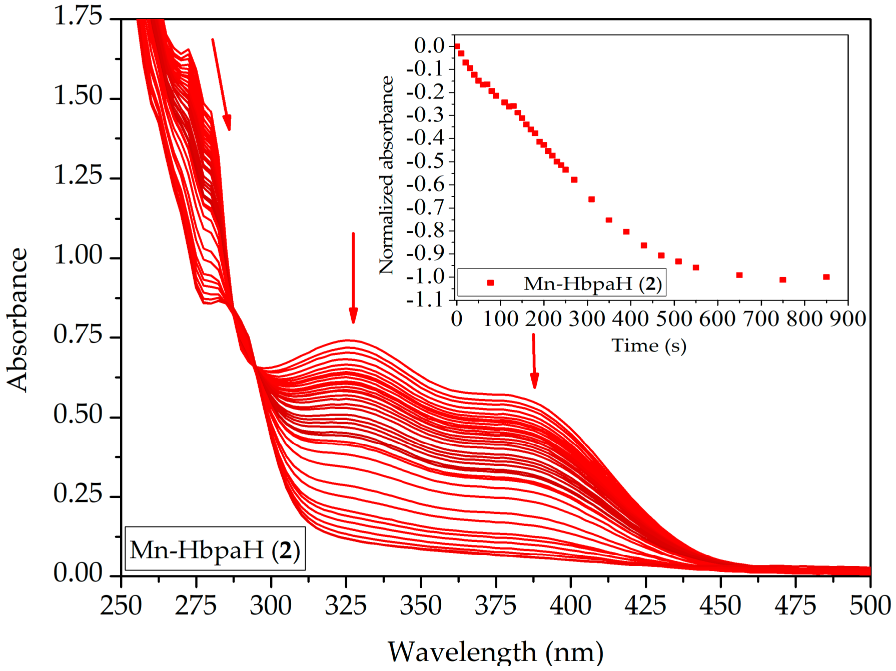

Figure S32. Changes in UV-Vis spectrum of **(2)** in dichloromethane (2.53×10^{-4} mol L^{-1}) during UV light irradiation (λ_{em}=395±5 nm). Inset: Normalized absorbance decay of **(2)** at 379 nm as a function of time (s) during light exposure.

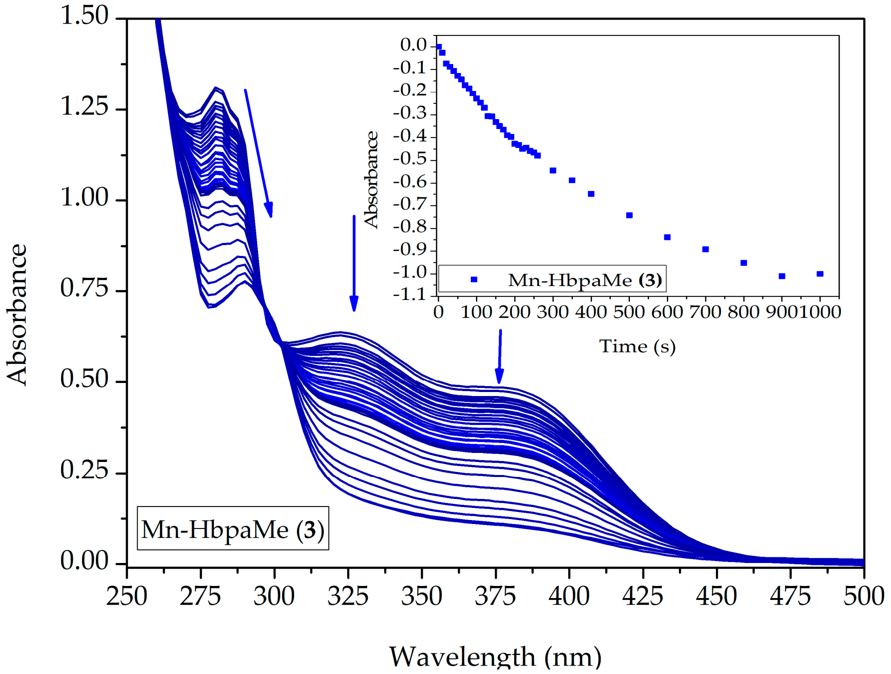

Figure S33. Changes in UV-Vis spectrum of **(3)** in dichloromethane (2.21×10^{-4} mol L^{-1}) during UV light irradiation (λ_{em}=395±5 nm). Inset: Normalized absorbance decay of **(3)** at 379 nm as a function of time (s) during light exposure.

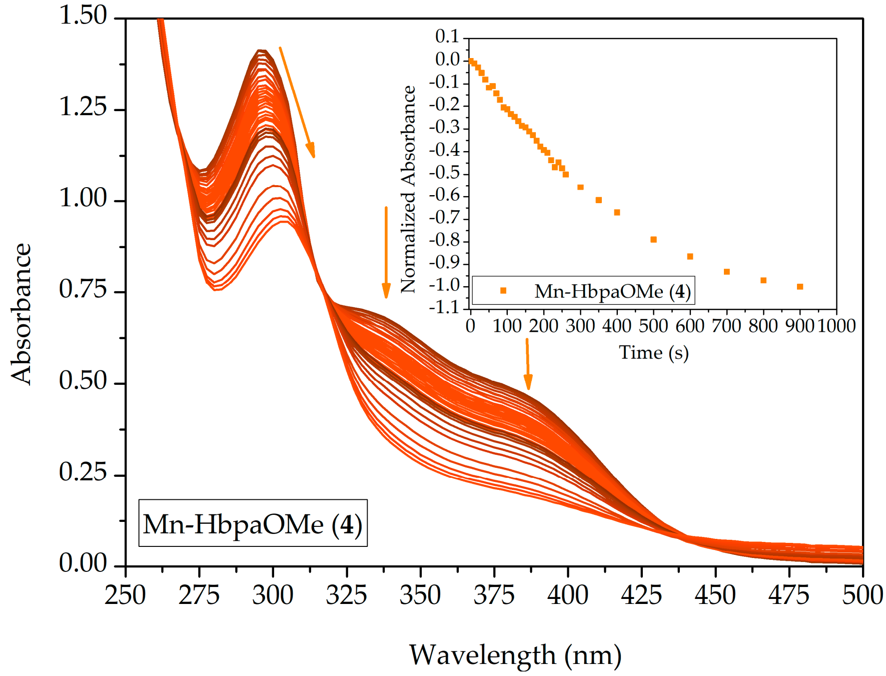

Figure S34. Changes in UV-Vis spectrum of **(4)** in dichloromethane (2.38×10^{-4} mol L^{-1}) during UV light irradiation (λ_{em}=395±5 nm). Inset: Normalized absorbance decay of **(4)** at 379 nm as a function of time (s) during light exposure.

Equations used to determine k_{CO}, $t_{1/2}$ and k_{CO}^{old}

$$A = \frac{A_t - A_i}{A_i - A_\infty}$$

The normalized absorbance (A) is equal to the absorbance on a given time (A_t), minus the absorbance on time = 0s (A_i), divided by the absorbance at the initial time minus the absorbance at the final time (A_∞). Using a simple exponential decay law, the decomposition rate of the initial specie arises as t_1:

$$A = A_1 \times \exp\left(\frac{-t}{t_1}\right) + A_0$$

A_1 and A_0 are adjustment parameters originated from the exponential fit and t is the respective time associated with the normalized absorbance A. CO release rate k_{CO}^{new} is described as the inverse of t_1:

$$-k_{CO}^{new} = -\frac{1}{t_1}$$

The half-life time was also deduced by this method, where the obtained parameters from Equations 1, 2 and 3, results in Equation 4:

$$t_{1/2} = -t_1 \times \ln\left(\frac{\frac{A_\infty}{2} - A_0}{A_1}\right)$$

CO release rate was also determined by the old method, using a pseudo-first-order linearization, where:

$$\ln(A) = at + b$$

$$k_{CO}^{old} = -a$$

k_{CO}^{old} is defined as the negative slope of the linear equation (-a).

Cartesian coordinates of optimized structure

Coordinates for **(1)** optimized in vacuum.

Mn	2.28577626053446	2.72542687562736	1.97080299442334
Br	0.47889328470751	3.50705240688061	3.59710505288622
C	1.30540207837969	3.40456467030369	0.59571049866006
O	0.69105536688047	3.83850691884537	-0.26750073988780
C	3.10878100000541	4.33863244546639	2.18606715434078
O	3.62649311265491	5.34861364645917	2.33038223542176
C	3.55792400852256	2.11998394896441	0.84692108438381
O	4.36100236862198	1.74281330108608	0.11409034400977
N	3.17248479169272	1.84968136260157	3.69656615834354
H	2.46899355683935	2.10727673792865	4.39111121822017
C	3.16066059771823	0.39065714428999	3.51505867295860
H	3.30649325034977	-0.14665025678189	4.45504064048401
H	3.99559602062068	0.12639139861251	2.85843870985835
C	1.86979571273289	-0.01779226864118	2.87207937658820
N	1.32053913625453	0.88782049124884	2.04788943211960
C	0.16366001676722	0.59121415106685	1.44537204022369
H	-0.26214026101901	1.35900733271053	0.81695869604363
C	-0.47823696376114	-0.62126195685169	1.62144572473454
H	-1.41182867104660	-0.81075836889771	1.11075365046031
C	0.08953256202596	-1.56196476394277	2.46799944301130
H	-0.39133511939804	-2.51663445959729	2.63645767556245
C	1.27816847121502	-1.24942411603805	3.10870733245835
H	1.74552033968282	-1.94542421404163	3.79235144762048
C	4.48216960242260	2.36718187018011	4.15427564471328
H	4.33753130947988	3.42430119887327	4.37307138820703
H	5.18538837281347	2.29117158266718	3.32517930542623
C	5.04759348183673	1.66556790423484	5.36097222734324
C	4.38452416730796	1.72868442158126	6.59158196335186
C	4.90434681971814	1.08687100857165	7.70855422171915
H	4.38364968728275	1.14356461553738	8.65786720535798
C	6.09344664241427	0.37410669048481	7.61604151692858
H	6.49832207467151	-0.12239803279405	8.48641453272907
C	6.75098252946262	0.30991583890225	6.39880515922972
C	6.23308070809623	0.94687808517284	5.28056320791104
H	6.75738733535292	0.89207034968007	4.33578300671539
Br	8.38576543279999	-0.66149341467182	6.24973319405336
O	3.21790865312146	2.43744623106732	6.61756931873614
H	2.84560826223800	2.45349022321305	7.50689626465258

Coordinates for **(2a)** optimized in vacuum.

Mn	7.06804776747994	6.66223539901797	19.80711781464138
Br	7.05486080986418	5.37512057795269	22.01705130623710
C	8.87684375010664	6.46633853316009	19.74759685637364
O	10.01473795738387	6.34145333051068	19.71951903741103
C	6.85753302937164	5.06863124645141	18.94799213004407
O	6.72457590897808	4.06950947658844	18.40660190536307
C	7.03134503393153	7.57404469453387	18.25277441240356
O	7.01599721990376	8.13235796066462	17.24621495869014
N	4.99737937851641	6.94634839181381	20.20052175764193
H	4.94685964032910	6.49562301808894	21.11596552926031
C	4.76541741225544	8.38896940099092	20.36009581490900
H	3.81487160878010	8.60607644994609	20.85293217223899
H	4.72132892587683	8.83579403434089	19.36157823815165
C	5.90363337633524	8.99197443483098	21.12554299356771
N	7.09038072623922	8.38376748626411	20.97475890364168
C	8.14808457094464	8.86486597150739	21.63815080073999
H	9.07692061937427	8.32772159917734	21.51789197613383
C	8.07252599410546	9.98073671366497	22.45225490734284
H	8.95806953588568	10.33012536826919	22.96417115694157
C	6.84962327386654	10.61815966312266	22.60177265261657
H	6.75311647027233	11.48906692748740	23.23685454191355
C	5.74748661046657	10.10764812033129	21.93459845847974
H	4.77060493581867	10.56023910866114	22.03941615218157
C	3.99611202211703	19.32250313588042	6.29610668771543
H	4.20569459524035	5.22758985018942	19.35721917115981
H	4.16798986873118	6.63776546906425	18.30165104402419
C	2.56410214874451	6.55746576553279	19.70955875005056
C	2.08347738274835	6.13329050401364	20.95362626430314
C	0.76520415116669	6.36602090194420	21.32576640270217
H	0.41159192620170	6.03023732708627	22.29449138118233
C	-0.09332793485304	7.02250271632454	20.45237667600649
H	-1.12031731804418	7.19752505209830	20.74612876263618
C	0.36305394813843	7.44962869072297	19.21303561937316
H	-0.30340798744658	7.95798186847552	18.52944220903185
C	1.68646113792301	7.21583056851936	18.85661230208217
H	2.05033521876852	7.54545807685446	17.89041749639791
O	2.98000927561177	5.49782952917763	21.76623411229381
H	2.55894900886590	5.21735408490395	22.58693619594994

Coordinates for **(2b)** optimized in vacuum.

Mn	3.64421097275516	5.91898677180935	14.26945900545366
Br	3.70916738527286	7.96049804793842	15.81561324254156
C	5.23709002750858	5.35842192686492	14.94821648555144
O	6.24121252122443	5.00431475427525	15.37028828995946
C	2.76044594543717	5.02371098622969	15.59300083863948
O	2.20867821880280	4.46770725840614	16.42683775563239
C	3.54843381584989	4.52534086794926	13.13251215810740
O	3.48314418440310	3.63099237885050	12.41095639087339
N	1.90071913488161	6.84023077393440	13.47315896518290
H	1.86963958076931	7.66534228872518	14.07505175064936
C	2.19124297360904	7.25668721530128	12.09304174731805
H	1.49443611312381	8.01681292806378	11.73267271493104
H	2.07166067809995	6.37926554533055	11.44900500261770
C	3.60330039150668	7.74691045724655	11.99459833940418
N	4.47414727887879	7.18929847968218	12.84960388466230
C	5.75478440429123	7.57414264387998	12.80474373639588
H	6.41889283109726	7.12432439553188	13.52738245896349
C	6.22099507632298	8.51014263181872	11.89942889893056
H	7.26638032778717	8.78488268499345	11.90485503125410
C	5.32350270252408	9.08781311211421	11.01368713977966
H	5.65130748109147	9.83267927910301	10.30041617399612
C	3.99323573223942	8.70411682939196	11.06987531207811
H	3.25664792433988	9.14253155262130	10.41005445665342
C	0.59622062597490	6.14292646491881	13.58563925642737
H	0.45890738216712	5.90594974157626	14.63923786595727
H	0.66709702154207	5.20327387381247	13.03729825355001
C	-0.57320166175467	6.94581571600582	13.08109817784212
C	-0.93425177383854	8.12711166099993	13.73953066301211
C	-2.00568986799901	8.89197417023707	13.29576776152594
H	-2.27205980834850	9.80288707281325	13.82039216004947
C	-2.73227916499733	8.48174824727106	12.18454271123873
H	-3.56799323097630	9.07966538503707	11.84432848795586
C	-2.39045500181477	7.31339711988026	11.51708693761208
H	-2.95624209258134	6.99048542287612	10.65354360729968
C	-1.31403241330502	6.55882593390604	11.97072869092045
H	-1.04275446373646	5.64406200334228	11.45634455921340
O	-0.17269565596733	8.47290087098002	14.81961481182533
H	-0.48993259618155	9.29228650628133	15.21632127599406

Coordinates for **(3)** optimized in vacuum.

Mn	9.05424081895463	5.45242322034445	11.44681139087923
Br	10.86936498650688	6.25370946998817	9.83743781606064
N	8.21727164453150	4.52062528247524	9.72831581500457
H	8.91358247219936	4.78748686489327	9.03056843892924
C	9.98935957113159	6.19343363862277	12.82181805184627
O	10.57315932071192	6.66878063619721	13.68478864843247
C	8.18593325706560	7.03331390435072	11.18048824387452
O	6.97953664981883	4.44485987138293	13.29006637335276
C	7.78242166076035	4.83250909499552	12.56191982637821
O	7.63917242542558	8.02277197592988	11.00400996418133
C	8.27604526390141	3.06593897520479	9.93119734871412
H	8.16241475605569	2.51235590835706	8.99647286075203
H	7.43989040805799	2.78241249004468	10.57825927306338
C	9.56852601877264	2.70962278822889	10.60125741774515
N	10.07527975592806	3.64419484491949	11.42045261589305
C	11.22992140761824	3.39326404275042	12.04747802206409
H	11.62044487394399	4.18381590518432	12.67062053993624
C	11.91240795403276	2.19908080846229	11.90178733702612
H	12.84231833957924	2.04686576260601	12.43126905862626
C	11.38933935576458	1.22866627086388	11.05995086279784
H	11.90305185655483	0.28743767815848	10.91426288099079
C	10.20340686468801	1.49429815508455	10.39382948989818
H	9.77105973925218	0.77430443421470	9.71205265860980
C	6.89239705241371	4.98459861012539	9.25227754065836
H	7.00453051507875	6.04268777625296	9.01846166731525
H	6.18837931399683	4.89987865976060	10.08008498708205
C	6.35352537466622	4.24790427207558	8.05509144542937
C	7.03679163432204	4.28756688515741	6.83535019813858
C	6.53386236227530	3.61992303704562	5.72854177691917
H	7.07144217951089	3.65231727267781	4.78713017206525
C	5.34024257930388	2.91372198886433	5.82763324620828
H	4.95803778691543	2.40025377127272	4.95356880774172
C	4.63261925387230	2.85733885445039	7.02388704116534
C	5.16808160383199	3.52887584202984	8.12351668177940
H	4.63840706096267	3.50096237405047	9.07005893042659
C	3.32290903436254	2.12321472449258	7.13170631953391
H	3.16972695655781	1.45959823317612	6.28028225861295
H	2.48117258656432	2.82072490564919	7.16233892678118
H	3.27670759588644	1.52083559931691	8.04125282216001
O	8.20664456461682	4.99881891595704	6.81095708258063
H	8.58269714360561	5.00176625438506	5.92337716034501

Coordinates for **(4a)** optimized in vacuum.

Mn	7.72864145799990	9.00467002054779	0.89729107511982
Br	5.99344982629163	10.67554726961463	0.04327245727406
C	6.64204513311859	7.66452080305129	0.32084393959984
O	5.94713456742778	6.83218313862070	-0.04793332913199
C	8.51788750174178	9.06817415377737	-0.74707554558918
O	9.00330890579509	9.11041835854581	-1.78231645918783
C	8.95746055050080	7.85734185891206	1.55061510839251
O	9.73615850061428	7.11493005254850	1.95901935780418
N	8.71946660847831	10.73550622281733	1.63754376259254
H	8.06711105256776	11.43815119192450	1.28352656266783
C	8.65692280253036	10.71555306579031	3.10677905597698
H	8.81789975495777	11.70477895606031	3.54156393466842
H	9.46621367732516	10.07178204089390	3.46679370700196
C	7.34518474932533	10.15019714545139	3.55657703131111
N	6.78523193398434	9.24965587777177	2.73432677176132
C	5.61925330566135	8.69667214576827	3.08835819870892
H	5.18581216706423	7.99690345521477	2.38989343832003
C	4.97555193660748	9.00510319892184	4.27312259327417
H	4.03420993855552	8.52936706616515	4.50953490837291
C	5.55059779294662	9.93800849234002	5.12347133702968
H	5.06717852954459	10.21416084758021	6.05145037705401
C	6.75153256992368	10.52196763823084	4.75380445728525
H	7.22754824538870	11.26617216787641	5.37804777380926
C	10.07340762832377	11.08418491892538	1.14264548738070
H	10.01978941851145	11.08688257085977	0.05553790749467
H	10.75928778286619	10.29233789221451	1.44419561463585
C	10.56830525748150	12.41889027824509	1.63468067848146
C	9.90349714158940	13.58627342432762	1.23539752400928
C	10.33640813442615	14.82142763668575	1.68293537713627
H	9.82128915299925	15.72193402684054	1.36778059785259
C	11.43560443256087	14.92215841150253	2.53473064582536
H	11.75304335937411	15.89923393915342	2.86662688343496
C	12.10153876691114	13.77103304479794	2.93890103181181
C	11.65661161016549	12.52858472935896	2.48245579500768
H	12.18792329731492	11.64144065253367	2.80419687240250
C	13.68127223289020	14.98797639588089	4.24472258659141
H	14.00905041046778	15.62798019926144	3.41987963700024
H	14.53447041282658	14.74840550030069	4.87490080062842
H	12.93111725601867	15.51947512709632	4.83857946484150
O	8.82729278866903	13.41563543996076	0.40372956593230
H	8.41759643618443	14.26211195693921	0.19367295467443
O	13.18363797206764	13.74702168668988	3.77105106074267

Coordinates for **(4b)** optimized in vacuum.

Mn	7.67939628730904	6.15208122187992	7.24232186502902
Br	8.86239739773204	3.92227144047295	6.81088500412389
C	8.93822147695003	7.00369427015093	6.24134358326769
O	9.73034403252906	7.53918801992773	5.61080639070418
C	8.72326826572904	6.34383191768113	8.72697692561409
O	9.37945577549369	6.46076844392804	9.65688493811078
C	6.77874082398050	7.67647157733555	7.57056259741573
O	6.20385092094720	8.64863849632531	7.79270004612966
N	6.21779703000576	4.95462297959888	8.20728392936407
H	6.63876406827512	4.03353536704804	8.07309264554825
C	4.97157071030448	5.03353124061545	7.43043407052670
H	4.45642150816164	5.95673784432270	7.71484361850683
H	4.29281048209578	4.20731799553313	7.65307770452699
C	5.28572727357200	5.07831675267567	5.96622508071710
N	6.46347116151706	5.63155438850545	5.63883621435884
C	6.79850339829561	5.71082063042477	4.34566898047427
H	7.76655856292589	6.13440432624611	4.12386589428602
C	5.96960242274110	5.26530017613021	3.33193628815259
H	6.28762518728740	5.35533499668029	2.30281737665184
C	4.74927489247148	4.69714848449014	3.66691867627726
H	4.08053434373784	4.32917517711904	2.89972809706786
C	4.40882365131221	4.59524718976263	5.00610624615127
H	3.47552025132025	4.14271978683357	5.31322133472061
C	5.99339474645680	5.14843526192482	9.65988634685290
H	5.70617203060791	6.18721318555173	9.82182504587387
H	6.95366977121895	4.98605628561099	10.14676330155108
C	4.95607016930381	4.23192166125589	10.25049172014277
C	5.17529967224291	2.84837721852727	10.26098684925502
C	4.22581422502106	1.99836463675024	10.79901735295971
H	4.40020966543898	0.92819369646692	10.80610343174989
C	3.04525239608942	2.50486733471487	11.34130026713087
H	2.32413597222313	1.81858566264816	11.75915140066978
C	2.81856257207252	3.87612153349928	11.33768404852950
C	3.77930442220937	4.72510817335933	10.78549846585678
H	3.58885494451506	5.79118200136471	10.79068304225757
C	0.71216442951576	3.66452402822176	12.43238278397133
H	0.29155695464155	2.95985983858452	11.70825223969916
H	-0.06966269799398	4.33872806558762	12.77382486120414
H	1.11120457389601	3.11009554905486	13.28726234563258
O	6.35491819115209	2.41498329772011	9.71133560710306
H	6.42856555600056	1.45632064027774	9.77470725722539
O	1.70545848069355	4.48555020519123	11.84000012460851
